# Boosting genome editing efficiency in human cells and plants with novel LbCas12a variants

**DOI:** 10.1186/s13059-023-02929-6

**Published:** 2023-04-30

**Authors:** Liyang Zhang, Gen Li, Yingxiao Zhang, Yanhao Cheng, Nathaniel Roberts, Steve E. Glenn, Diane DeZwaan-McCabe, H. Tomas Rube, Jeff Manthey, Gary Coleman, Christopher A. Vakulskas, Yiping Qi

**Affiliations:** 1 Integrated DNA Technologies, Coralville, IA 52241 USA; 2 Current Address: Aera Therapeutics, 50 Northern Ave, Boston, MA 02210 USA; 3 Department of Plant Science and Landscape Architecture, University of Maryland, College Park, MD 20742 USA; 4 Current Address: Syngenta, 9 Davis Dr, Research Triangle, NC 27709 USA; 5 Department of Applied Mathematics, University of California-Merced, Merced, CA 95343 USA; 6 Institute for Bioscience and Biotechnology Research, University of Maryland, Rockville, MD 20850 USA

**Keywords:** Protein evolution, LbCas12a-RVQ, LbCas12a-RV, LbCas12a-RRV, Genome editing, Human cells, Rice, Tomato, Poplar

## Abstract

**Background:**

Cas12a (formerly known as Cpf1), the class II type V CRISPR nuclease, has been widely used for genome editing in mammalian cells and plants due to its distinct characteristics from Cas9. Despite being one of the most robust Cas12a nucleases, LbCas12a in general is less efficient than SpCas9 for genome editing in human cells, animals, and plants.

**Results:**

To improve the editing efficiency of LbCas12a, we conduct saturation mutagenesis in *E. coli* and identify 1977 positive point mutations of LbCas12a. We selectively assess the editing efficiency of 56 LbCas12a variants in human cells, identifying an optimal LbCas12a variant (RVQ: G146R/R182V/E795Q) with the most robust editing activity. We further test LbCas12a-RV, LbCas12a-RRV, and LbCas12a-RVQ in plants and find LbCas12a-RV has robust editing activity in rice and tomato protoplasts. Interestingly, LbCas12a-RRV, resulting from the stacking of RV and D156R, displays improved editing efficiency in stably transformed rice and poplar plants, leading to up to 100% editing efficiency in *T*_0_ plants of both plant species. Moreover, this high-efficiency editing occurs even at the non-canonical TTV PAM sites.

**Conclusions:**

Our results demonstrate that LbCas12a-RVQ is a powerful tool for genome editing in human cells while LbCas12a-RRV confers robust genome editing in plants. Our study reveals the tremendous potential of these LbCas12a variants for advancing precision genome editing applications across a wide range of organisms.

**Supplementary Information:**

The online version contains supplementary material available at 10.1186/s13059-023-02929-6.

## Background

The CRISPR-Cas9 system has revolutionized the field of precise genome engineering in a broad range of species, from mammalian cells to plants. Despite the wide application of Cas9, Cas12a (formerly known as Cpf1) [1–3], a class II type V CRISPR nuclease, has features that offer advantages over Cas9. These features include the TTTV protospacer adjacent motif (PAM) requirement in contrast to SpCas9’s NGG PAM requirement, simplified DNA targeting by crRNA while Cas9 requires both tracrRNA and crRNA (or sgRNA when combined), self-processing of the crRNA array due to Cas12a’s intrinsic RNase activity, and creation of staggered ends after DNA cleavage as opposed to blunt ends generated by Cas9. To expand the utility of Cas12a, researchers have engineered Cas12a to enhance editing efficiency (enAsCas12a, opAsCas12a, AsCas12a Ultra, and AsCas12a-Plus) [4–7], temperature tolerance (LbCas12a-D156R) [4, 8], and relaxed PAM requirements (reviewed in ref. [2]). Although there have been advances in AsCas12a editing activity through protein engineering, few of them have sufficient editing activity for genome editing in plants [2]. Since LbCas12a has shown enhanced editing performance compared to other Cas12a proteins in plants, it is imperative to engineer improved LbCas12a variants to boost CRISPR-Cas12a based genome editing in plants [9].

We recently engineered an AsCas12a Ultra (M537R/F870L) variant that resulted in significant improvement of genome editing in human cells [5]. To engineer novel LbCas12a variants, we first tried to mimic the point mutations from AsCas12a Ultra (M537R/F870L) to LbCas12a based on protein sequence alignment. However, the LbCas12a (N527R/E795L) variant failed to show improved editing efficiency in human cells. As a consequence, we performed an unbiased high throughput screen in *E. coli* of mutants that spanned the entire coding sequence of LbCas12a and discovered a number of point mutations that improved LbCas12a’s editing activity in *E. coli*. After thorough validation of selected mutations in human cells, a LbCas12a variant with three sta**c**ked point mutations (G146R/R182V/E795Q), named LbCas12a-RVQ, showed the highest editing efficiency of all the variants. However, LbCas12a-RV showed robust editing efficiency in rice and tomato protoplasts. Furthermore, LbCas12a-RRV (addition of D156R to LbCas12a-RV) showed even greater editing efficiency in transgenic rice and poplar, even at the non-canonical TTV PAM sites. Taken together, we report LbCas12a-RVQ and LbCas12a-RRV as novel LbCas12a variants that show enhanced genome editing in in human cells and plants, respectively, compared to wild-type (WT) LbCas12a and LbCas12a-D156R (also known as ttLbCas12a [8]). These engineered LbCas12a variants should facilitate robust genome editing in eukaryotic organisms.

## Results

### Directed evolution of LbCas12a with enhanced activity

To enhance the on-target activity of LbCas12a, we first attempted to mimic the mutations of AsCas12a Ultra (AsCas12a: M537R/F870L) in LbCas12a based on sequence alignment (Additional file 1: Fig S1A) [5]. However, incorporating both mutations in LbCas12a (i.e., N527R/E795L) did not increase the on-target activity when delivered as RNP (Additional file 1: Fig S1B). Specifically, we found E795L enhanced the editing efficiency at HPRT 38,330 site using 0.4 μM RNP, but outweighed by the detrimental effect of N527R (Additional file 1: Fig S1B). These results indicate the impact of point mutations on closely related Cas12a orthologs at aligned positions can be highly variable. As a result, previously identified beneficial mutations from AsCas12a Ultra cannot simply be used to improve LbCas12a function. We therefore proceeded to conduct a de novo screen to identify mutations in LbCas12a that improve intrinsic enzymatic activity.

**Fig. 1:**
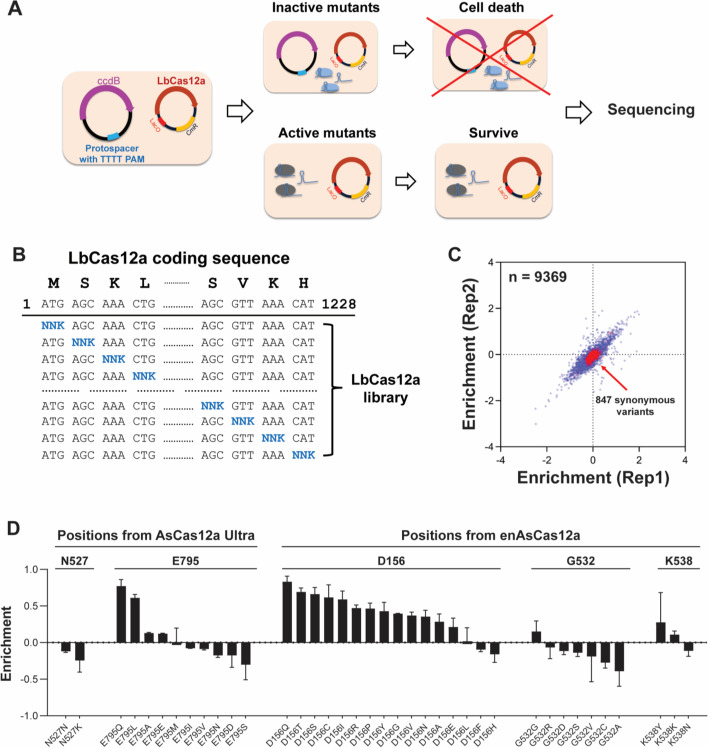
High-throughput characterization of DNA cleavage activities of LbCas12a point mutations in *E. coli*. **A** Schematic representation of bacterium-based selection assay to isolate LbCas12a mutants with enhanced activity. *E.coli* BW25141:DE3 cells containing an inducible *ccdB* expression plasmid were transformed with LbCas12a variant library, which was programmed to cleave the reporter plasmid through a target site with TTTT PAM sequence. Active LbCas12a mutants with enhanced activity enable the clearance of reporter plasmid, thus avoiding the cell death upon the induction of *ccdB* expression with arabinose. LbCas12a plasmids from survived cells were extracted and used for subsequent round of selection. Four rounds of sequential selection were performed. Round 3 and 4 libraries were sequenced and used to calculate the enrichment of LbCas12a variants. **B** Design of a saturation mutagenesis library for LbCas12a. Every codon of LbCas12a was randomized with NNK degenerate primers by nicking mutagenesis [10]. Importantly, each member of plasmid library only contains one codon change at a time, which was verified by Sanger sequencing of 24 individual plasmids from the library. **C** Enrichment scores for ~ 9000 LbCas12a point mutations over the last round of selection (round 4). Synonymous variants without changes on the protein sequences were colored in red. Variants with a minimal of 50 counts were included in this analysis. **D** Enrichment scores for 5 selected positions of LbCas12a that have been evaluated prior to this study

To accomplish this, we designed an unbiased screen that systematically evaluates the effect of all single point mutations of LbCas12a on DNA cleavage in *E. coli* (Fig. 1A, B). We first established an *E. coli*-based activity assay for LbCas12a, where the degree of cell survival upon *ccdB* (which encodes a toxin) induction is directly linked to the cleavage activity of LbCas12a (Fig. 1A). We next generated a saturation mutagenesis library of LbCas12a spanning the entire coding sequence, with most plasmid clones in the library only containing one codon change (Fig. 1B) [10]. This library was then iteratively selected for four rounds and the enrichment of LbCas12a variants in the last round of selection was quantified by next generation sequencing (NGS). This strategy allowed us to measure the phenotype of over 9000 point mutations with high reproducibility (Fig. 1C). As expected, nearly all synonymous mutations (847) displayed no significant enrichment, as their scores tightly clustered around 0. There were 1977 point mutants with phenotype scores (i.e., natural logarithm of relative enrichment) greater than zero over three biological replicates (Additional file 2: Table S1).

The effect of point mutations on enzyme activity can depend on the experimental system and conditions. In the context of mutation screens for CRISPR enzymes in *E. coli*, false positives have been reported in the literatures [11–13] where positive responses in bacteria failed to translate in human cells. To evaluate the reliability of our high-throughput data generated in *E. coli*, we first checked the phenotype scores of mutations known to affect the intrinsic cleavage activity of LbCas12a. For those two positions (N527/E795) taken from AsCas12a Ultra, N527 suffered from significant reductions in enrichment scores, indicating mutations at this position are detrimental to DNA cleavage in *E. coli* (Fig. 1D). While the enrichment score of N527R is not available in this analysis, a lysine substitution at this site (i.e., N527K) showed measurable reduction of cleavage activity of LbCas12a in *E. coli*, suggesting a positive charged side chain at position 527 for LbCas12a is detrimental to function. On the other hand, the E795L mutation was enriched upon selection (Fig. 1D), which is consistent with our empirical observations when the corresponding LbCas12a variants were delivered as RNP in human cells (Additional file 1: Fig S1B).

Previous research with AsCas12a showed that both E174R and S542R mutations significantly enhanced on-target activity [14]. Moreover, K548R mutants were also altered in PAM preference with little effect on overall activity [14]. Therefore, we attempted to introduce similar mutations from enAsCas12a (E174R/S542R/K548R) into LbCas12a to generate enLbCas12a (D156R/G532R/K538R). However, only D156R exhibited any beneficial effect, whereas the other two mutations are detrimental to editing [15]. This observation is faithfully recapitulated by our *E. coli* screen (Fig. 1D). For position D156, nearly all amino acid changes were enriched over the selection process, suggesting the removal of negative charge at the side chain of position 156 is the underlying feature to improve on-target activity. In contrast to the D156 site, most point mutations with measurable enrichment scores at positions G532 or K538 are detrimental, including the G532R introduced into enLbCas12a. In addition, we found significant data dropouts at both positions, further indicating that both positions are not amendable for mutagenesis (Fig. 1D). Taken together, we provided examples where the enrichment scores of 5 different mutation sites correlated well with the known effects on the intrinsic activity of LbCas12a. While we cannot rule out the absence of false positives, we are confident that the mutations represent true positive hits with enhanced activity in our dataset.

### LbCas12a-RVQ is the optimal nuclease with robust activity in human cells

**Fig. 2:**
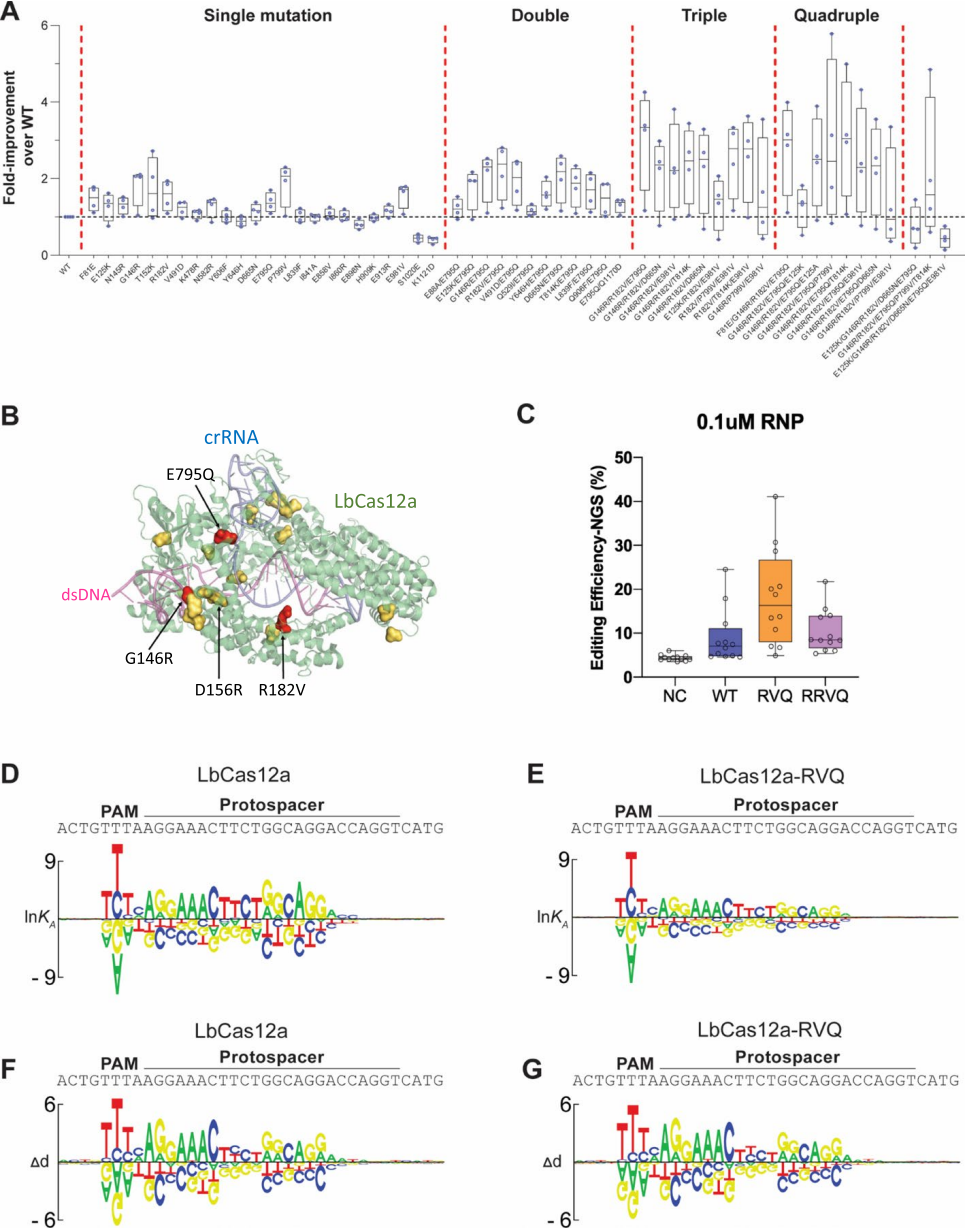
LbCas12a-RVQ is a novel variant with robust editing efficiency in human cells. **A** The normalized editing efficiencies of 56 LbCas12a variants in human HEK293 cells over four target sites when delivered as RNP. See Additional file 1: Fig. S2A for the raw editing efficiency. **B** Mapping the validated positive hits with enhanced activity on the existing LbCas12a structure. Red: residues used in LbCas12a-RVQ constructs. Gold: other residues with improved activity but not included in the LbCas12a-RVQ. **C** Editing efficiency of LbCas12a-WT, LbCas12a-RVQ and LbCas12a-RRVQ in HEK293 cells over additional 11 target sites at a low RNP dosage (0.1 μM). NC indicates negative control. One-way analysis of variance (ANOVA) with Tukey’s multiple comparison test (*p* < 0.05) were analyzed using GraphPad Prism 9. **D**, **E** The DNA binding specificities of WT LbCas12a (**D**) and LbCas12a-RVQ (**E**) measured by Spec-seq over the same target site and presented as motif logo for visualization. **F**, **G** The DNA cleavage specificities of WT LbCas12a (**F**) and LbCas12a-RVQ (**G**) measured by SEAM-seq

To further validate the editing performance of some mutations in human cells, 1977 positive mutation were ranked by the phenotype (i.e., enrichment score; Additional file 2: Table S1) and then prioritized over residues on the DNA/R-Loop binding interface, which ended up with 24 point mutations. We characterized the on-target editing efficiency of 24 novel LbCas12a point mutations in human cells using RNP delivery. Purified LbCas12a proteins were programmed with 4 different synthetic crRNAs to edit the human *HPRT* gene in HEK293 cells, and the efficiency was measured by T7EI assay 2 days post-delivery (Additional file 1: Fig S2A). The relative activity of each mutant over WT LbCas12a was shown in Fig. 2A. Overall, we found 16 of the 24 mutations enhanced the on-target editing efficiency of LbCas12a. Mapping the validated hits on the existing crystal structure of the LbCas12a-crRNA-dsDNA complex (PDB: 5XUS [16]) revealed that most hits were in proximity to dsDNA or DNA/RNA duplexes (Fig. 2B). We then combined these validated hits in various double and triple mutation combinations and identified a specific variant with 3 mutations (RVQ: G146R/R182V/E795Q) that performed the best overall (Fig. 2A, B). We also generated and tested some higher-order quadruple and quintuple mutants which however did not show improved editing efficiency; Rather, editing efficiency was drastically reduced with some combinations (Fig. 2A). For example, combining more positive hits on RVQ, such as RVQVK (RVQ + P799V + T814K), reduced the overall efficiency of LbCas12a when delivered as RNP (Fig. 2A). Overall, our analysis identified RVQ as a top-performing LbCas12a variant for robust genome editing in human cells.

We further expanded the evaluation of LbCas12a-RVQ over an additional 11 sites by targeting a variety of therapeutic relevant targets. Cas12a-RNPs were intentionally delivered to HEK293 cells at low dosage (0.1 μM) via nucleofection, to better reveal the activity differences between these nucleases. As the baseline, WT LbCas12a showed a median editing efficiency at 7.4%. while LbCas12a-RVQ significantly enhanced the editing efficiency (median efficiency: 18.8%) (Fig. 2C). However, combining RVQ with D156R (RRVQ) compromised the overall efficiency (Fig. 2C). We thus concluded that the RVQ variant is a robust LbCas12a nuclease for human genome editing via RNP delivery.

We next characterized the intrinsic sequence specificity of LbCas12a-RVQ in vitro by SEAM/Spec-seq [16]. The DNA cleavage and binding specificities of LbCas12a RNPs (WT or RVQ) were measured over a library with sequences with up to 4 mismatches to the crRNA (Additional file 1: Fig S2B). Highly consistent measurements of both binding and cleavage specificities were obtained from two biological replicates (Additional file 1: Fig S2C). Biophysical models that describe the quantitative penalties of mismatches at each position of the target site were generated by non-linear regression and presented as motif logos for visualization (Fig. 2D–G). In general, the overall profiles of binding and cleavage specificities are highly similar between WT and RVQ, where the first 18-bp of crRNA mediates most sequence specificities. Incorporating RVQ mutations did not alter any base preference at each position, including the PAM region. As the trade-off for the enhanced activity, we observed slightly reduced binding and cleavage specificities of RVQ over the entire target site, which is analogous to the difference between WT AsCas12a and AsCas12a Ultra under the identical experimental condition [5]. As the mutations in the AsCas12a Ultra marginally affected the off-target profile in human cells [5], we reasoned that LbCas12a-RVQ largely maintained the intrinsic sequence specificity of WT LbCas12a, but with enhanced activity.

### LbCas12a-RV confers robust singular and multiplexed editing in rice and tomato protoplasts

Previous studies have shown that the editing efficiency of Cas12a varied from human cells to plant species and in general Cas12a nucleases are sensitive to temperature [3, 17–19]. Although various engineered Cas12a proteins have been reported to possess enhanced temperature tolerance and relaxed PAM requirements [2, 20], the editing efficiency is still relatively low in most plant species. There is a clear need to enhance the editing activity of Cas12a in plants at low temperatures that are more relevant to plant tissue culture. Therefore, we evaluated the editing efficiency of our engineered LbCas12a variants in the protoplasts of rice (a monocot crop) and tomato (a dicot crop) at both 32 °C and 25 °C.

**Fig. 3:**
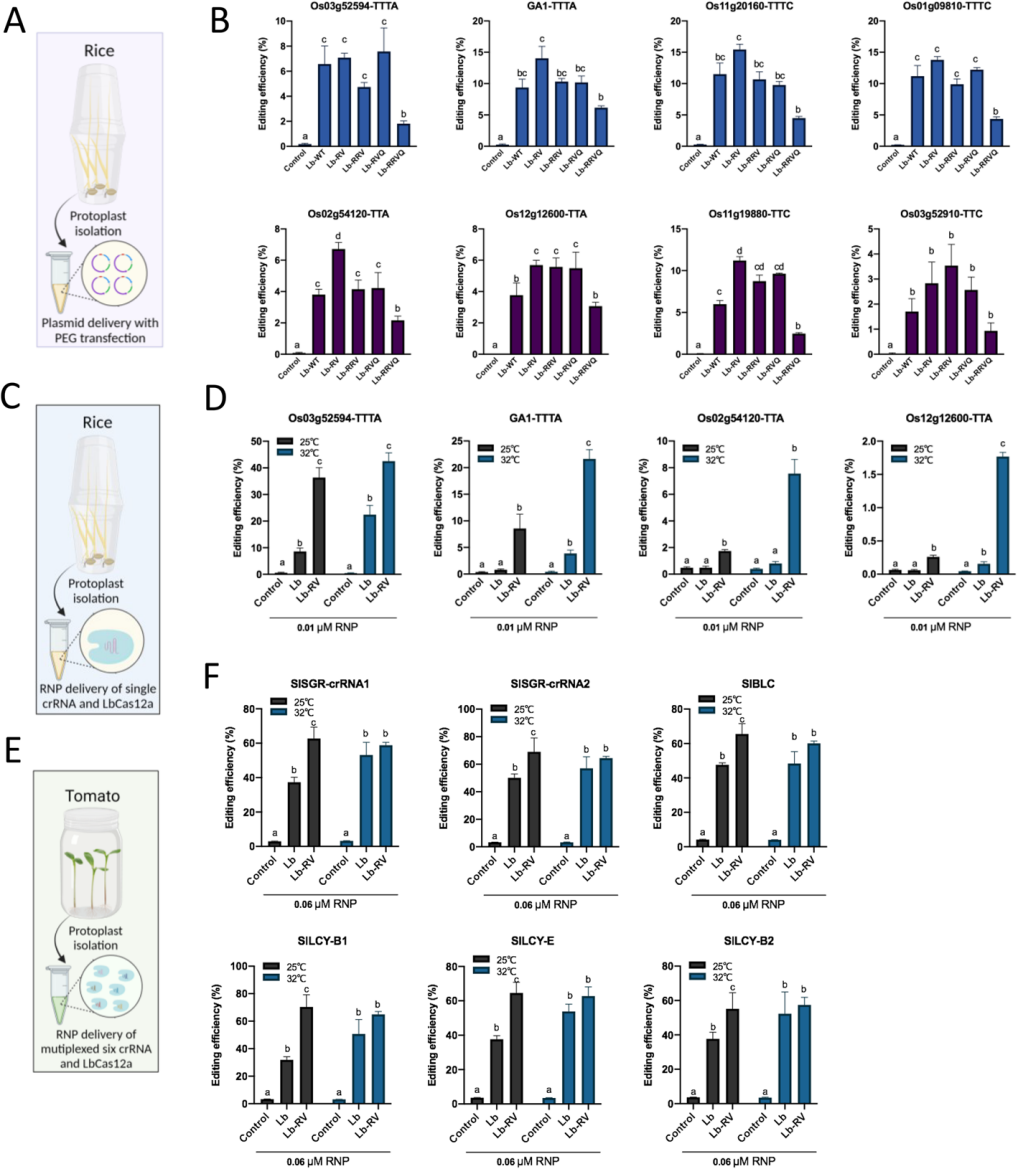
Enhanced editing efficiency of LbCas12a-RV in rice and tomato protoplasts using plasmid and RNP delivery. **A** Diagram of the plasmid expression system of Cas12a and crRNA. **B** Editing efficiency of LbCas12a variants at four TTTV target sites and four VTTV target sites in rice protoplasts. **C** Diagram of RNP delivery in rice protoplasts. Rice protoplasts were transfected with 0.01 μM RNP and incubated in two temperature conditions (25 °C and 32 °C) for 2 days. **D** Editing efficiency of LbCas12a and LbCas12a-RV at two TTTV target sites and two VTTV target sites in rice protoplasts with RNP delivery. **E** Diagram of RNP delivery in tomato protoplasts for multiplexed genome editing. Tomato protoplasts were transfected with 0.06 μM RNP (0.01 μM RNP per target) and incubated in two temperature conditions (25 °C and 32 °C). **F** Editing efficiency of LbCas12a and LbCas12a-RV at six target sites in tomato protoplasts with RNP delivery. The editing efficiency was measured by NGS. One-way analysis of variance (ANOVA) with Tukey’s multiple comparison test (*p* < 0.05) were analyzed using GraphPad Prism 9

Besides LbCas12a-RVQ and LbCas12a-RRVQ, we included another two new variants LbCas12a-RV (G146R/R182V) and LbCas12a-RRV (G146R/D156R/R182V) in the rice protoplast assay (Fig. 3A). LbCas12a-RV enabled highest editing efficiency at the 6 out of 8 target sites, while LbCas12a-RRV was comparable with LbCas12a-RVQ at all target sites (Fig. 3B). LbCas12a-RRVQ showed compromised editing efficiency as similarly observed in human cells (Figs. 2C and 3B). We further compared LbCas12a-RV with WT LbCas12a in rice protoplasts by editing four target sites with RNP delivery (Fig. 3C). At low RNP dosage (0.001 μM), LbCas12a-RV showed significantly higher editing efficiency than WT LbCas12a at all four target sites at 32 °C (Additional file 1: Fig S3A). The same trend was observed at two sites (Os03g52594-TTTA and GA1-TTTA) when performing the editing at a lower temperature (25 °C), although the efficiency was globally reduced for both WT-LbCas12a and LbCas12a-RV (Additional file 1: Fig S3A). As expected, increasing the dosage from 0.001 μM to 0.01 μM enhanced the editing efficiency for all the nucleases (Fig. 3D and Additional file 1: Fig S3A). At this higher concentration, LbCas12a-RV showed significantly higher editing efficiency than WT LbCas12a in rice protoplasts across all four target sites at both 25 °C and 32 °C (Fig. 3D).

We next evaluated LbCas12a variants in tomato protoplasts by simultaneously targeting 6 sites with RNP delivery (Fig. 3E). To keep the same RNP concentration for each target site in tomato protoplasts as in rice protoplasts, 0.006 μM and 0.06 μM RNP were used. At 0.006 μM RNP concentration, LbCas12a-RV resulted in significantly higher editing than WT LbCas12a at 5 out of 6 sites at 25 °C and at all 6 sites at 32 °C (Additional file 1: Fig. S3B). At this low RNP concentration, LbCas12a-RV displayed significantly higher editing efficiency at 32 °C than at 25 °C (Additional file 1: Fig. S3B). At 0.06 μM RNP concentration, genome editing efficiencies by WT LbCas12a and LbCas12a-RV were both high at 32 °C, about 60% at all target sites (Fig. 3F). At 25 °C, LbCas12a-RV sustained the same high editing efficiencies of 32 °C at all 6 sites (Fig. 3F). By contract, WT LbCas12a’s editing efficiencies were all dropped significantly at this relatively low temperature (Fig. 3F). Taken together, genome editing by LbCas12a-RV appears to be less sensitive to temperature than WT LbCas12a at all target sites in rice and tomato protoplasts with the higher RNP concentration (Fig. 3D, F) and at most target sites with the lower RNP concentration (Additional file 1: Fig S3). Thus, the LbCas12a-RV variant is robust for plant genome editing at variable temperatures. To further verify the editing efficiency of the new LbCas12a variants, they were subsequently tested in transgenic rice and poplar.

### LbCas12a-RRV outperforms other variants in stable transgenic rice

**Fig. 4:**
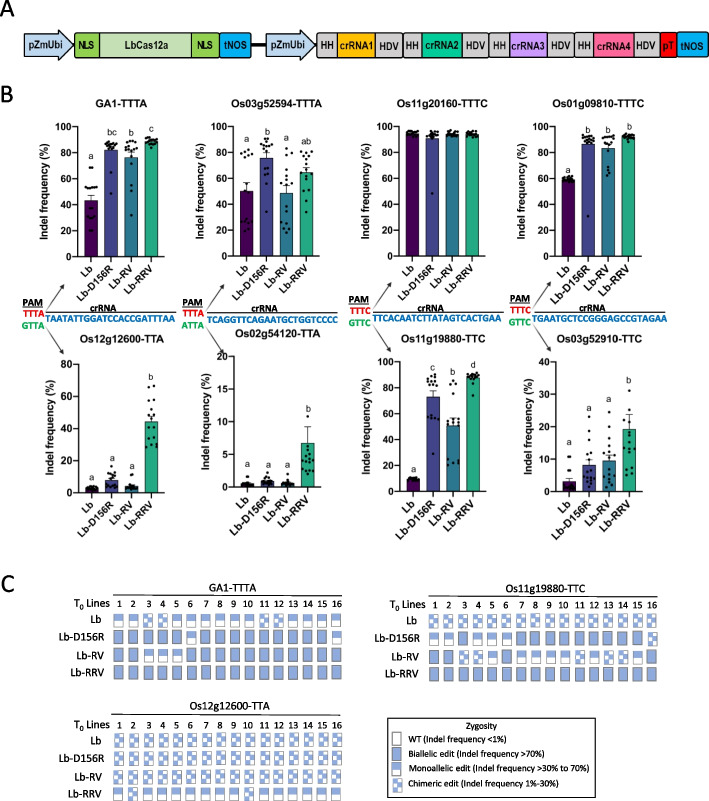
Robust genome editing by LbCas12a-RRV in rice *T*_0_ transgenic plants. **A** Diagram of multiplexing four crRNAs to simultaneously target eight sites using a dual ZmUbi promoter and tandem HH-crRNA-HDV system. **B** Indel frequencies of eight target sites in rice *T*_0_ plants with each dot representing one *T*_0_ plant. **C** Genotypes of *T*_0_ plants at GA1-TTTA, Os11g19880-TTC and Os12g12600-TTA sites. Wild type (WT) denoted as empty rectangles, biallelic edit denoted as fully filled rectangles, monoallelic edit denoted as half-filled rectangles, chimeric edit denoted as doted rectangles. One-way analysis of variance (ANOVA) with Tukey’s multiple comparison test (*p* < 0.05) were analyzed using GraphPad Prism 9. Bars without assigned letters indicate no significant differences among four LbCas12a nucleases

*Agrobacterium*-mediated stable transformation of CRISPR reagents is the primary delivery method for obtaining genome-edited plants. To evaluate the performance of our LbCas12a variants in transformed plants generated by this common method, we first compared the editing efficiency of WT, D156R, RV, and RRV versions of LbCas12a in rice *T*_0_ plants using a multiplexed Cas12a/crRNA expression system (Fig. 4A) [21]. Four target sites were selected with each being duplicated in the rice genome with the identical protospacers but with different PAMs: canonical TTTV vs non-canonical VTTV PAMs (Fig. 4B). In total, eight target sites were targeted simultaneously by four crRNAs (Fig. 4A, B). Of the 4 canonical TTTV sites, 2 sties (GA1-TTTA and Os01g09810-TTTC) showed significantly greater indel frequencies with all three variants and one site (GA1-TTTA) showed significant difference between LbCas12a-RRV and LbCas12a-RV (Fig. 4B). LbCas12a-RRV generated greater biallelic editing compared to the other variants with 100% of the tested lines for the GA1-TTTA site showing biallelic editing (Fig. 4C and Additional file 1: Fig S4). LbCas12a-RRV consistently showed higher indel frequencies compared to the other variants and WT LbCas12a at the four non-canonical VTTV PAM sites (Fig. 4B, C and Additional file 1: Fig S4B, D). Impressively, LbCas12a-RRV achieved 100% biallelic editing at 4 out of 8 target sites (GA-TTTA, Os11g19880-TTC, Os11g20160-TTTC, and Os01g09810-TTTC), whereas other Cas12a variants only generated 100% biallelic editing at one site, Os11g20160-TTTC (Fig. 4C and Additional file 1: Fig S4C, E). These results suggest that LbCas12a-RRV is a promising and robust Cas12a nuclease for generating genome edited rice plants not only at canonical TTTV PAM sites but also non-canonical VTTV PAM sites.

### LbCas12a-RRV outperforms other variants in stable transgenic poplar

**Fig. 5:**
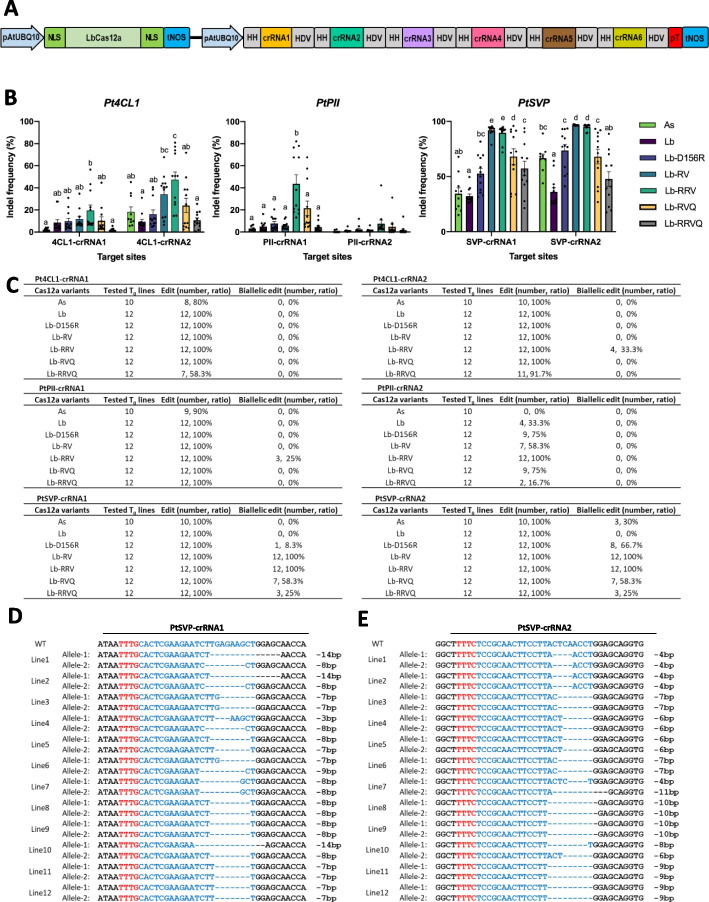
Robust genome editing by LbCas12a-RRV in poplar *T*_0_ transgenic plants. **A** Diagram of multiplexing six crRNAs to simultaneously target six sites using a dual pAtUBQ10 promoter and tandem HH-crRNA-HDV system. **B** Indel frequencies of six target sites in poplar *T*_0_ plants with each dot representing one *T*_0_ plant. **C** Editing efficiencies at six target sites. **D** Genotypes of 12 *T*_0_ plants at SVP-crRNA1 by LbCas12a-RRV. **E** Genotypes of 12 *T*_0_ plants at SVP-crRNA2 by LbCas12a-RRV. One-way analysis of variance (ANOVA) with Tukey’s multiple comparison test (*p* < 0.05) were analyzed using GraphPad Prism 9

Poplar is a perennial tree that also serves as an important bioenergy and biomaterials feedstock in foundational and translational research. Biallelic genome editing in the first generation is necessary for functional analysis of the target genes in trees like poplar. To further evaluate the performance of LbCas12a-RRV, we used *Agrobacterium* to deliver LbCas12a/crRNAs into poplar. Six sites were simultaneously targeted using a multiplexed expression system (Fig. 5A). Since AsCas12a was previously reported to show higher editing efficiency than LbCas12a in poplar [22], AsCas12a was also included for comparison with LbCas12a variants. Based on the editing efficiency of WT-LbCas12a, there are 2 high-activity (SVP-crRNA1 and SVP-crRNA2) and 4 low-activity sites (4CL1-crRNA1, 4CL1-crRNA2, PII-crRNA1, and PII-crRNA2) (Fig. 5B). At the 2 high-activity sites in *PtSVP* (SVP-crRNA1 and SVP-crRNA2), LbCas12a-RV and LbCas12a-RRV showed 100% biallelic editing efficiency, significantly better than LbCas12a-D156R, LbCas12a-RVQ, LbCas12a-RRVQ, LbCas12a, and AsCas12a (Fig. 5B, C). At the 4 medium-to-low activity sites in *Pt4CL1* and *PtPII*, LbCas12a-RRV showed significantly higher editing activity than all other Cas12a proteins tested (Fig. 5B). Although biallelic edits were detected for each of the LbCas12a variants, they were more frequent for LbCas12a-RRV (4 of 6 sites) than LbCas12a-D156R (2 of 6 sites) and LbCas12a-RVQ (2 of 6 sites) (Fig. 5C). Sequencing analysis of the two *PtSVP* target sites in T_0_ lines showed that LbCas12a-RRV resulted in 100% biallelic editing with deletions ranging from 3 to 14 bp and with homozygous mutation rates of 50% and 83.3%, respectively (Fig. 5D, E). Therefore, LbCas12a-RRV is more robust and efficient for poplar genome editing than other LbCas12a variants and AsCas12a. It is advantageous to use LbCas12a-RRV over other Cas12a nucleases/variants in poplar due to its high frequency biallelic and homozygous editing.

## Discussion

In this study, we first attempted to enhance editing activity of LbCas12a by introducing similar point mutations as those in AsCas12a Ultra, which however did not yield a LbCas12a with improved nuclease activity. This reinforced the challenge of improving one Cas12a ortholog’s editing activity by mimicking positive mutations from another Cas12a ortholog. Similar results were also observed when engineering enLbCas12a by creating the same point mutations from the engineered enAsCas12a [14]. As a consequence, we adopted a directed evolution platform that has been successfully used for SpCas9 [23] and AsCas12a [4]. In combination with a massive parallel saturation mutagenesis library, our screen efficiently covered the entire coding sequence of LbCas12a, which enabled the isolation of novel point mutations that enhanced the on-target nuclease activity of LbCas12a when delivered as RNP. Although the current workflow uses multiple rounds of selection to enrich for positive hits, further improvement can be made to simplify the process to a single-round of selection, with the potential to rapidly establish a complete sequence-to-function map for CRISPR enzymes using the powerful bacterium-based screening system.

By combining three positive mutations, based on our screen in *E. coli*, we created an LbCas12a-RVQ variant that showed more robust editing in human cells than WT LbCas12a. To further boost editing efficiency of LbCas12a-RVQ, D156R was combined with LbCas12a-RVQ to generate LbCas12a-RRVQ, which however led to compromised editing efficiency in both human cells (Fig. 2C) and plants (Figs. 3B and 5B). Interestingly, stripping of the “Q” from LbCas12a-RVQ (i.e., LbCas12a-RV) resulted in more robust editing in rice protoplasts (Fig. 3B). Addition of D156R to LbCas12a-RV resulted in LbCas12a-RRV, which showed robust editing efficiency in transgenic rice and poplar plants, outperforming other LbCas12a variants (Figs. 4B and 5B). As demonstrated previously, Cas12a nucleases of higher nuclease activity such as AsCas12a Ultra and enAsCas12a also have elevated off-target activity [5, 14]. Added affinity to the Cas12a nuclease could compensate for the deficit of a poor target and ultimately enable the editing at sub-optimal PAM sites. Our data in plants seem to support this model. For example, while LbCas12a-D156R and LbCas12a-RV could achieve high levels of genome editing at the canonical TTTV PAM sites in stable transgenic rice plants, it is LbCas12a-RRV variants that consistently showed higher editing activity across all four low-activity target sites with non-canonical VTTV PAMs (Fig. 4B). LbCas12a-RRV’s superior editing performance was further validated at the low-activity and canonical TTTV PAM sites in poplar (Fig. 5). Together, we demonstrated LbCas12-RVQ and LbCas12-RRV as next-generation Cas12a nucleases for efficient genome editing in human cells and in plants, respectively. Previously, FnCas12a and Mb2Cas12a were demonstrated to be able to target VTTV PAM sites [21, 24]. LbCas12a is the most predominantly used Cas12a ortholog for genome editing in many organisms, but its editing activity at the non-canonical VTTV PAM sites is low [25]. With significantly improved activity at the non-canonical TTV PAM sites, LbCas12a-RRV developed in this study would have promising applications in many organisms due to its broadened targeting scope and elevated editing activity.

Improved LbCas12a variants would potentially confer elevated editing activity at some off-target sites with high sequence similarity to the protospacers. We examined the off-target effects mediated by two independent protospacers in rice. Eight *T*_0_ lines were analyzed per Cas12a construct for the detection of edits at predicted top off-target sites. For the protospacer that simultaneously targets the GA1-TTTA site (Additional file 1: Fig S5A) and the Os12g12600-TTA site (Additional file 1: Fig S5B), LbCas12a variants (D156R, RV and RRV) indeed showed higher off-target mutation rates at most of the off-target sites when compared to WT LbCas12a (Additional file 1: Fig S5E and S5F). By contrast, for the protospacer that simultaneously targets Os11g20160-TTTC and Os11g19880-GTTC sites (Additional file 1: Fig S5C and S5D), no off-target mutations (expect at the OT7 site) were detected at all top 6 off-target sites by all four LbCas12a proteins tested (Additional file 1: Fig S5G and S5H). These data suggest enhanced promiscuous binding to DNA by the high-activity LbCas12a variants could contribute to off-target effects in a protospacer-dependent manner. However, with careful design of highly specific protospacers, such protospacer-dependent off-targeting effects can be eliminated, as demonstrated by the genome-wide off-target analyses of LbCas12a in plants [25–27].

Our near-saturation screen revealed a large pool of point mutations with strong effects that enhanced the efficiency of LbCas12a (Additional file 2: Table S1) and further demonstrated that combining selected mutations can be sufficient to generate a potent nuclease, such as LbCas12a-RVQ. This trend appears to hold true for other type V nucleases, such as Cas12i2 [28–30], CasΦ/Cas12j2 [31, 32], and Cas14 [33]. It is intriguing to ask why the natural evolution of these nucleases in bacteria did not reach supreme DNA binding affinity. Perhaps, nucleases with excess DNA binding affinity are detrimental or impact fitness of their native hosts whose genome sizes are substantially smaller than humans and other higher eukaryotes. This is best demonstrated by the characterization of SpCas9 in *E.coli*, where the overexpression of dCas9 severely affected cell growth due to uncontrolled or excessive DNA binding [34, 35]. Therefore, we speculate that in the native host environment most CRISPR nucleases do not evolve their full editing potential without specialized mechanism to regulate dosage effects or activity [36]. Hence, our protein engineering workflow provided a unique opportunity to improve the CRISPR system in a high-throughput and unbiased manner. Although we primarily focused on mutations to enhance the DNA binding affinity of LbCas12a, more mutations that act through distinct mechanisms are likely to be present in our data (Additional file 2: Table S1) and remain to be fully characterized and utilized. We anticipate that our engineered LbCas12a-RVQ, LbCas12a-RV, LbCas12a-RRV, and future variants from this study will help advance genome editing in eukaryotic cells.

## Conclusions

Through saturation mutagenesis-based protein evolution in *E. coli*, we identified 1977 positive point mutations that may enhance LbCas12a nuclease activity. Harnessing some of these mutations, we developed higher-order RVQ, RV, and RRV variants of LbCas12a for efficient genome editing in human cells and plants. Impressively, LbCas12a-RRV achieved up to 100% editing efficiency in *T*_0_ plants of rice and poplar, even at certain non-canonical TTV PAM sites. Hence, we have engineered novel LbCas12a variants that confer high-efficiency genome editing in a wide range of eukaryotic organisms.

## Methods

### LbCas12a protein expression and purification

The LbCas12a recombinant proteins were produced as previously described [5]. Briefly, DNA sequences encoding the C′-terminal 6 × His-tagged LbCas12a proteins were cloned into pET28a vector. Transformed *E. coli* BL21 (DE3) cells (EMD Millipore) were grown in TB medium with 50 μg/mL Kanamycin until OD_600_ reaches 0.6–0.8. Cells were chilled at 4 °C for 30 min, and the protein expression was induced using 1 mM IPTG for 12–16 h at 4 °C. Cells were harvested by centrifugation (4000 × g, 20 min, 4 °C) and resuspended in the lysis buffer (20 mM NaPO_4_, pH 6.8, 0.5 M NaCl, 15 mM imidazole, 10 mM CaCl_2_, and 10% glycerol) supplemented with protease inhibitor cocktail (Sigma: 11,873,580,001), DNaseI and lysozyme. The resuspended cells were lysed by passing through Avestin Emulsiflex C3 three times at 15,000 psi, 4 °C. The cell lysate was centrifuged at 14,000 × g for 40 min, and the soluble fraction was sequentially purified by Nickle affinity (HisTrap HP, 5 mL, Cytiva) and cation exchange chromatography (HiTrap Heparin, 5 mL, Cytiva). Purified protein was concentrated (Amicon centrifugal filter, 10 kDa) and dialyzed against storage buffer overnight (20 mM TrisHCl, pH 7.4, 0.3 M NaCl, 0.1 mM EDTA, 50% Glycerol, and 1 mM DTT). The protein concentration was determined by Nanodrop using an extinction coefficient at 167,780 M^−1^ cm^−1^.

### Human cell editing by RNP nucleofection

Human HEK293 cells (ATCC) were maintained in DMEM media (ATCC) supplemented with 10% Fetal Bovine Serum (FBS, ThermoFisher). To assemble RNP, purified LbCas12a protein was diluted in 1X PBS and incubated with synthetic crRNA at a molar ratio of 1:1.5 for 10 min. To deliver RNPs into HEK293 cells, 2 μL RNPs were mixed with 200,000 cells resuspended in 20 μL SF buffer (Lonza) and electroporated with Lonza 4D Nucleofector using DS-150–96 program. After nucleofection, cells were immediately resuspended with 100 μL pre-warmed DMEM media. Thirty to fifty microliters of resuspended cells were transferred to 100 μL DMEM + 10% FBS in a 96-well cell culture plate and incubated at 37 °C with 5% CO_2_ for 48 h.

Genome editing was assessed by T7EI assay or amplicon sequencing. The T7EI assay was performed as previous described [5]. Briefly, cells were washed with 1X PBS and lysed by 50 μL QuickExtract solution (Lucigen). The HPRT genomic region was PCR amplified, heat-denatured at 95 °C for 10 min, and gradually cooled down to 25 °C over 20 min to form heteroduplex containing mismatches. The heteroduplex was cleaved by T7 endonuclease I (NEB) in 1X CutSmart buffer for 1 h, and the cleavage reaction was resolved by capillary electrophoresis (Fragment Analyzer, Agilent). The percentage cleavage of full-length amplicon was measured and reported as the editing efficiency. For some experiments, amplicon sequencing was performed to determine the editing efficiency of Cas12a proteins. The target genomic region was amplified, barcoded using rhAmpSeq master mix (IDT) following the recommended protocol. PCR products were purified with Ampure XP beads (Beckman Coulter), and pooled for sequencing on an Illumina MiSeq instrument with 2 × 151 bp cycles. The sequencing data was processed by rhAmpSeq data analysis suite (IDT) to determine the editing efficiency.

### Spec-seq/SEAM-seq

The Spec-seq/SEAM-seq was performed as previously described [5]. DNA libraries were ordered as IDT Ultramer and pooled at equal molar ratio. The second strand was synthesized by Klenow extension using Spec-R primer and purified by Qiagen MinElute column. For Spec-seq, the nuclease-inactivated LbCas12a-RNP (dLbCas12a, 200 nM) was incubated with dsDNA library (50 nM) in 1X reaction buffer (20 mM HEPES, pH 7.5, 150 mM KCl, 10% glycerol, 5 mM MgCl_2_, and 1 mM DTT) at 37 °C for 1 h. The binding reaction was resolved by a 4–20% native PAGE using 1X Tris–Glycine supplement with 5 mM MgCl_2_ as the buffer system. The protein-bound and unbound fractions were excised and eletroeluted in 1X Tris–Glycine buffer to recover each DNA fraction. For SEAM-seq, the library was cleaved by WT Cas12a nuclease in 1X reaction buffer for 1 h and quenched with 50 mM EDTA. A mock treatment was performed as negative control by incubating library with 1X binding buffer. At the end of reaction, the remaining DNA was purified by MinElute columns. All fractions were PCR amplified with Spec-seq-F and IDT-i7 primers to prepare sequencing libraries. The final library pool was sequenced on an Illumina NextSeq instrument with 82 cycles. The sequencing data was processed as previously described [5].

### Directed evolution of LbCas12a in *E. coli*

The saturation mutagenesis library was prepared according to Wrenbeck EE et al. [10]. Primers with NNK randomized codon were ordered as Oligo pool (IDT) with 25-nt flanking region. The bacterial assay to determine the DNA cleavage activity of LbCas12a variants was performed as previously described [5]. *E. coli* BW25141 (DE3) strain was first transformed with a reporter plasmid containing P_BAD_-controlled *ccdB* toxin. Plasmid encoding LbCas12a under the control of T7 promoter was delivered into the reporter strain by electroporation and programmed to cleave the reporter plasmid. Electroporated cells were recovered by SOB media and incubated at 30 °C for 1.5 h prior to the induction of LbCas12a expression with 1 mM IPTG for 1 h. To select high-activity LbCas12a variants, cells were plated on LB-Chloramphenicol plates supplemented with 2% (w/v) arabinose and incubated at 37 °C overnight. The survived cells were collected and maxi-prepped to extract the LbCas12a expression plasmids. The LbCas12a coding sequence was amplified using LbCas12a-F/LbCas12a-R primers and then sequenced on Illumina NextSeq instrument as a Nextera library.

### Plant material and growth condition

Rice (*Oryza sativa* L) Japonica cv. Kitaake was used in this study. Fourteen-day-old seedlings grown on ½ MS medium in dark at 28 °C were used for protoplast isolation. Rice calli induced from sterile mature healthy seeds on N6D medium at 32 °C under light were used for stable transformation. *Populus alba x tremula* clone 717-1B4 grown on ½ LS medium at 25 °C in 16 h/8 h (light/dark) condition was used for stable transformation. *Solanum lycopersicum* cv. Micro-Tom was grown on ½ MS medium at 25 °C under dark condition for 1 week followed by one week of 16 h light/8 h dark condition at 25 °C. Fourteen-day-old seedlings were used for protoplast isolation.

### T-DNA vector construction

All T-DNA vectors used for single crRNA expression were constructed as previously described [37]. All T-DNA vectors for multiplexing crRNAs are constructed based on the user manual as described in Zhang et al. [21]. Briefly, multiple crRNAs were assembled into recipient vector using the Golden Gate reactions to construct the crRNAs expression vector. Then, crRNAs expression vector and Cas12a vector were assembled into destination vector pYPQ202 (Addgene ID86198) for poplar, pMDC32 for tomato, and pYPQ203 (Addgene ID86207) for rice to make the T-DNA vectors. The Gateway compatible vectors for LbCas12a variants are available at Addgene (pYPQ230-D156R: Addgene ID188542; pYPQ230-RV: Addgene ID188544; pYPQ230-RRV: Addgene ID188543; pYPQ230-RVQ: Addgene ID 200,187; pYPQ230-RRVQ: Addgene ID 200,188). All T-DNA vectors are summarized in Additional file 2: Table S2, and all the oligos used in this study are listed in Additional file 2: Table S3.

### Rice protoplast transformation

Rice protoplast isolation and PEG-mediated transformation were performed as previously described [21]. For RNP delivery, 20 μl assembled RNP mixture with different concentrations was used for PEG-mediated protoplast transfection as previously described [38]. Protoplast transformed with equal amount of water instead of plasmid was used as wide type control for all protoplast transformation experiments.

### Tomato protoplast transformation

Tomato protoplast isolation and transformation was performed as previously described [39]. Briefly, cotyledons of 14-day old tomato seedlings were used for protoplast isolation. Thirty micrograms of plasmid DNA was transfected into 200 μl protoplast by PEG-mediated transformation. Protoplast transformed with equal amount of water instead of plasmid was used as wide type control for all protoplast transformation experiments. After 48 h incubation, transformed protoplasts were lysed and PCR amplicons were generated using Phire Plant Direct PCR Mix (ThermoFisher).

### Rice stable transformation

Agrobacterium-mediated transformation was performed as previously [21]. Regenerated shoots were cultured at 29 °C in 16 h/8 h (light/dark) condition. Young leaves of *T*_0_ plants were used for DNA extraction using a CTAB method.

### Poplar stable transformation

*Agrobacterium*-mediated transformation was performed in poplar as described [40]. Regenerated shoots from selection medium containing hygromycin were transferred to rooting medium. Rooted plants were propagated and cultured at 25 °C in 16 h/8 h (light/dark) condition. Young leaves were mixed with dilution buffer from Phire Plant Direct PCR Kit (ThermoFisher) to be further used for genotyping.

### Next-generation sequencing

To detect genome editing, barcoded PCR amplicons from protoplast samples and *T*_0_ plants were used for next-generation sequencing (Genewiz, USA). Sequencing data was analyzed using HiTom tool [41] and CRISPRMatch [42].

### Off-target analysis

To analyze the off-target effects of LbCas12a variants, three top potential off-target sites of GA1-TTTA, Os12g12600-TTA, Os11g20160-TTTC, and Os11g19880-TTC were selected from CRISPR-P v2.0 (http://crispr.hzau.edu.cn/CRISPR2/). Eight *T*_0_ plants of each LbCas12a variant were used for PCR reactions using Phire Plant Direct PCR Mix (ThermoFisher). The PCR amplicons were purified and used for next-generation sequencing (Genewiz, USA). Sequencing data was analyzed using HiTom tool [41].

## Supplementary Information

Additional file 1: Figure S1. Double mutations on LbCas12atransferred from AsCas12a Ultra reduced editing efficiency in HEK293 cells. Figure S2. Editing efficiency of 24 novel LbCas12a mutant proteins in HEK293 cells using the T7EI assay. Figure S3. Targeted mutagenesis by LbCas12a variants in rice and tomato protoplasts using low-concentration RNP delivery. A, single crRNA was delivered using 0.001 µM RNP in rice protoplast; B, multiplex 6 crRNAs were delivered using 0.006 µM RNP in tomato protoplast. Figure S4. Genotypes of T0 plants at 5 target sites of LbCas12a variants. Figure S5. Off-target analysis of LbCas12 variants in T0 rice plants.As per journal requirements, every additional file must have a corresponding caption. In this regard, please be informed that the caption was taken from the additional e-file itself. Please advise if the action taken is appropriate and amend if necessary.The captions look good!

Additional file 2: Table S1. Positive mutations identified in the LbCas12a saturation screen. Table S2. T-DNA vectors used in this study. Table S3. Oligos used in this study.

Additional file 3. Review history.

## Data Availability

Sequencing raw data was deposited to SRA BioProject (accession number PRJNA854643) [43]. Genome editing vectors are available at Addgene.
