# Supplementary material for: Boosting genome editing efficiency in human cells and plants with novel LbCas12a variants
Source: Genome Biol. 2023 Apr 30;24:102. doi: 10.1186/s13059-023-02929-6 (PMC10150537; doi:10.1186/s13059-023-02929-6)
Supplement: Supplementary file 1 — Additional file 1: Figure S1. Double mutations on LbCas12atransferred from AsCas12a Ultra reduced editing efficiency in HEK293 cells. Figure S2. Editing efficiency of 24 novel LbCas12a mutant proteins in HEK293 cells using the T7EI assay. Figure S3. Targeted mutagenesis by LbCas12a variants in rice and tomato protoplasts using low-concentration RNP delivery. A, single crRNA was delivered using 0.001 µM RNP in rice protoplast; B, multiplex 6 crRNAs were delivered using 0.006 µM RNP in tomato protoplast. Figure S4. Genotypes of T0 plants at 5 target sites of LbCas12a variants. Figure S5. Off-target analysis of LbCas12 variants in T0 rice plants.As per journal requirements, every additional file must have a corresponding caption. In this regard, please be informed that the caption was taken from the additional e-file itself. Please advise if the action taken is appropriate and amend if necessary.The captions look good! [file 13059_2023_2929_MOESM1_ESM.pptx]

## Slide 1
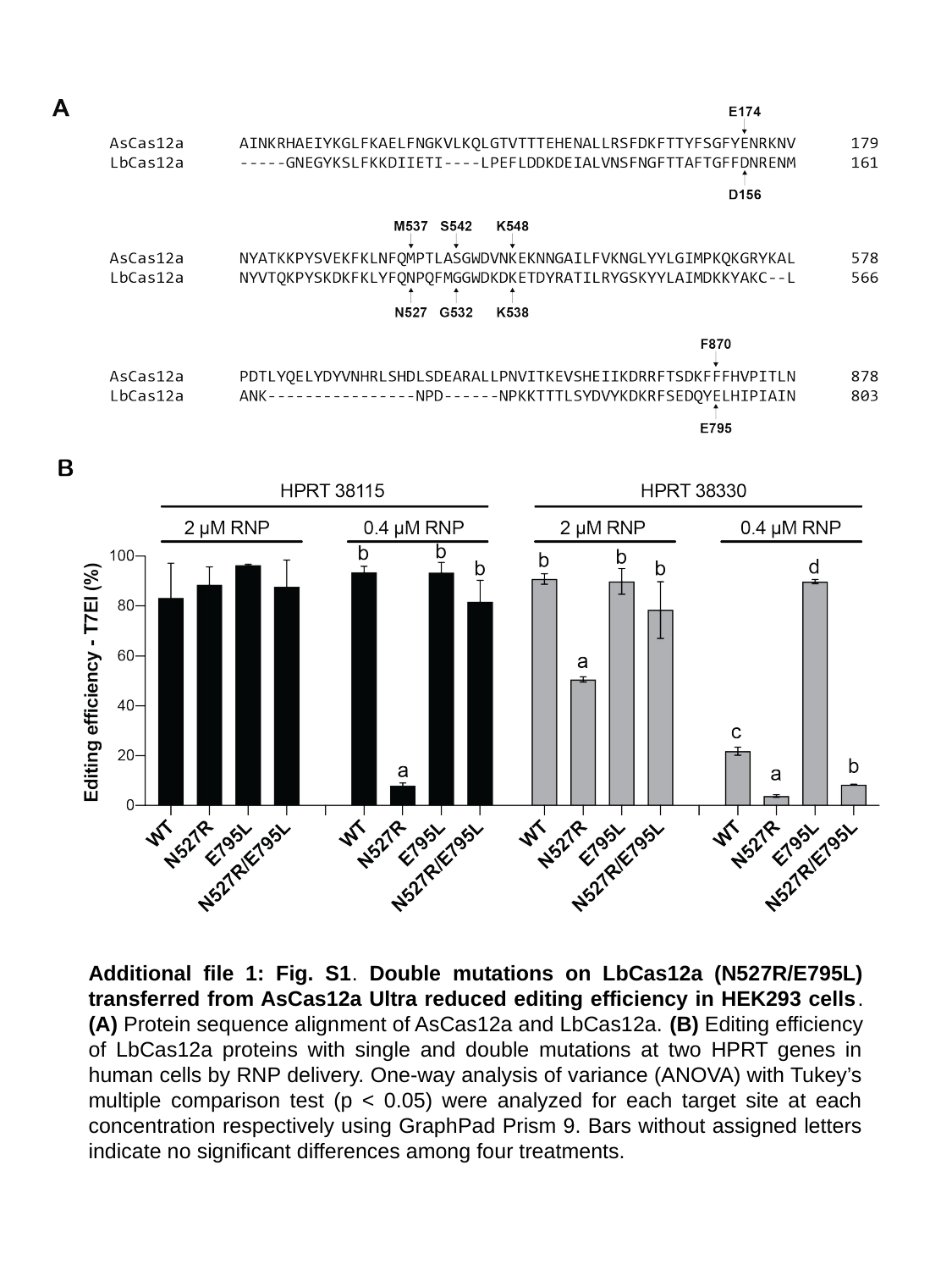

Additional file 1: Fig. S1. Double mutations on LbCas12a (N527R/E795L) transferred from AsCas12a Ultra reduced editing efficiency in HEK293 cells. (A) Protein sequence alignment of AsCas12a and LbCas12a. (B) Editing efficiency of LbCas12a proteins with single and double mutations at two HPRT genes in human cells by RNP delivery. One-way analysis of variance (ANOVA) with Tukey’s multiple comparison test (p < 0.05) were analyzed for each target site at each concentration respectively using GraphPad Prism 9. Bars without assigned letters indicate no significant differences among four treatments.

## Slide 2
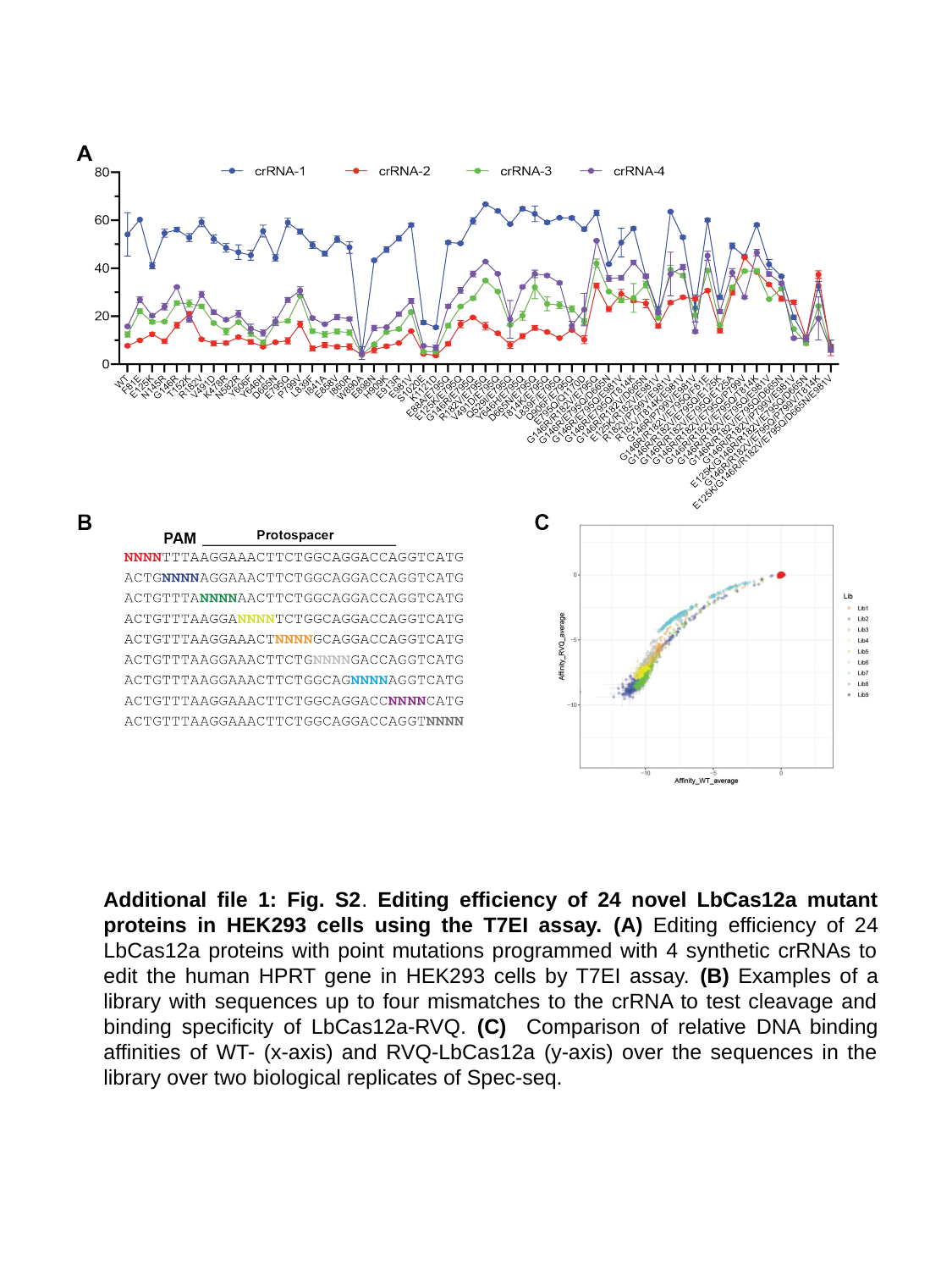

Additional file 1: Fig. S2. Editing efficiency of 24 novel LbCas12a mutant proteins in HEK293 cells using the T7EI assay. (A) Editing efficiency of 24 LbCas12a proteins with point mutations programmed with 4 synthetic crRNAs to edit the human HPRT gene in HEK293 cells by T7EI assay. (B) Examples of a library with sequences up to four mismatches to the crRNA to test cleavage and binding specificity of LbCas12a-RVQ. (C) Comparison of relative DNA binding affinities of WT- (x-axis) and RVQ-LbCas12a (y-axis) over the sequences in the library over two biological replicates of Spec-seq.

## Slide 3
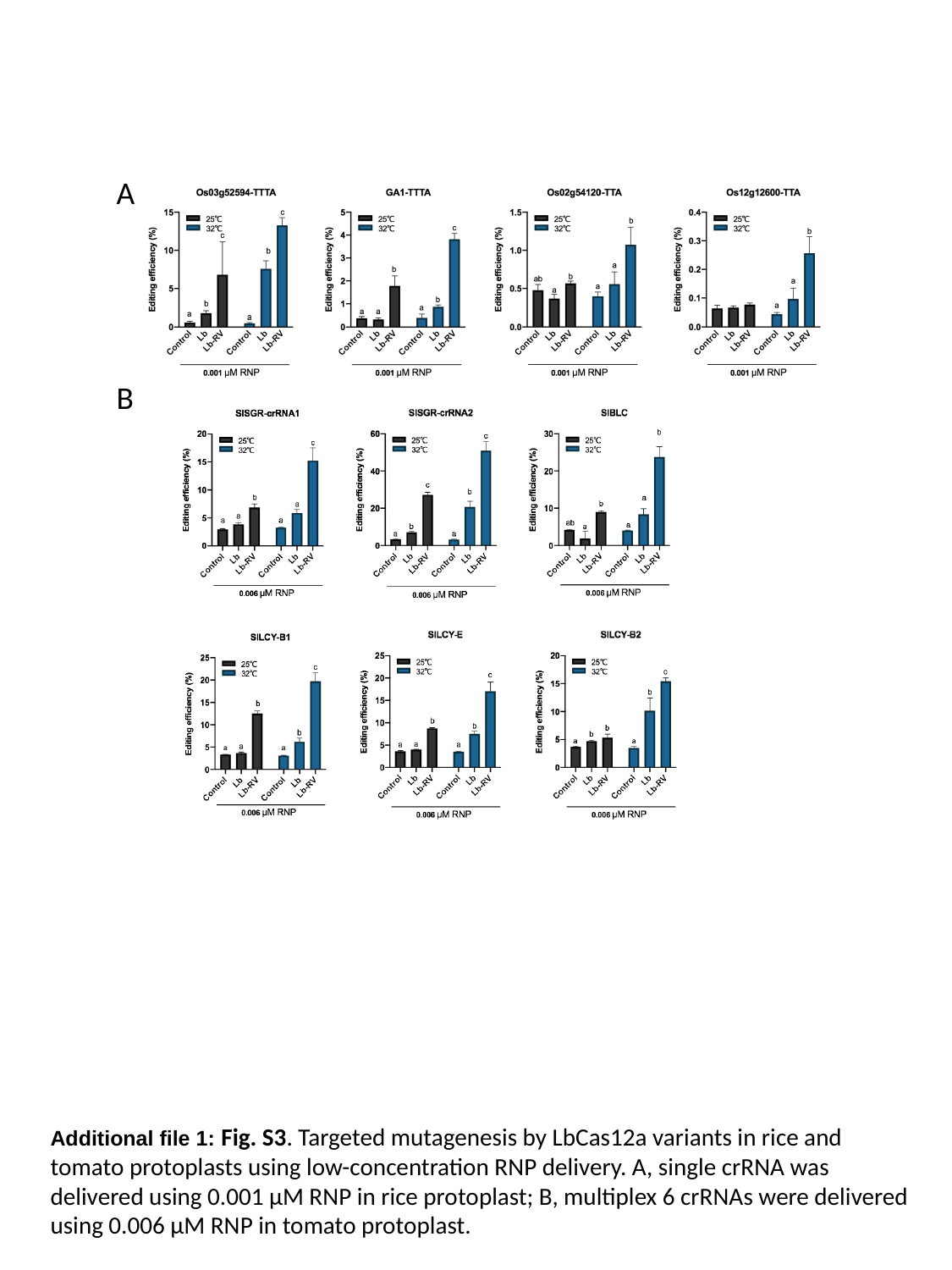

A
B
Additional file 1: Fig. S3. Targeted mutagenesis by LbCas12a variants in rice and tomato protoplasts using low-concentration RNP delivery. A, single crRNA was delivered using 0.001 µM RNP in rice protoplast; B, multiplex 6 crRNAs were delivered using 0.006 µM RNP in tomato protoplast.

## Slide 4
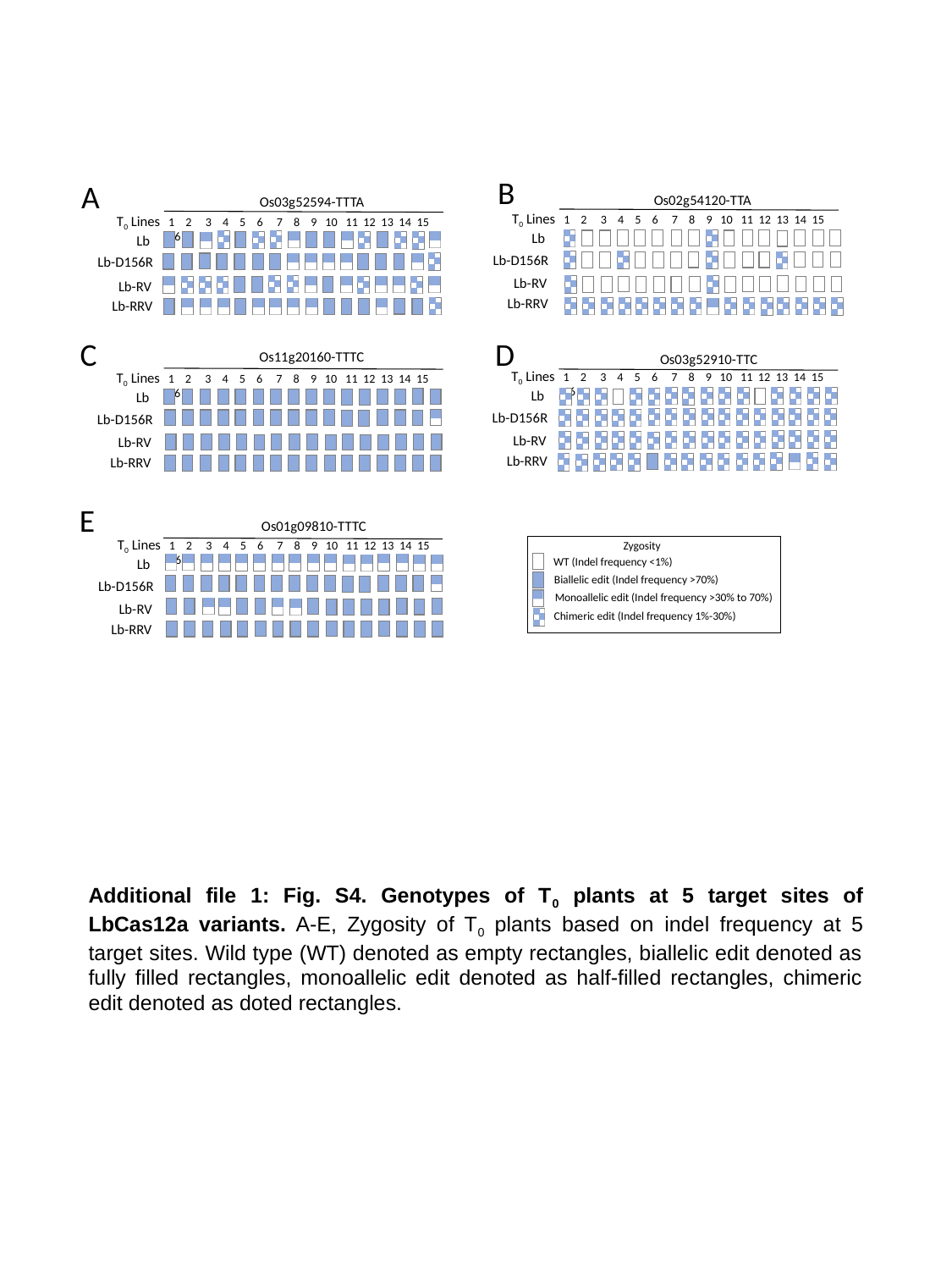

B
A
Os02g54120-TTA
Os03g52594-TTTA
T0 Lines
T0 Lines
1 2 3 4 5 6 7 8 9 10 11 12 13 14 15 16
1 2 3 4 5 6 7 8 9 10 11 12 13 14 15 16
Lb
Lb
Lb-D156R
Lb-D156R
Lb-RV
Lb-RV
Lb-RRV
Lb-RRV
C
D
Os11g20160-TTTC
Os03g52910-TTC
T0 Lines
T0 Lines
1 2 3 4 5 6 7 8 9 10 11 12 13 14 15 16
1 2 3 4 5 6 7 8 9 10 11 12 13 14 15 16
Lb
Lb
Lb-D156R
Lb-D156R
Lb-RV
Lb-RV
Lb-RRV
Lb-RRV
E
Os01g09810-TTTC
T0 Lines
1 2 3 4 5 6 7 8 9 10 11 12 13 14 15 16
Zygosity
WT (Indel frequency <1%)
Biallelic edit (Indel frequency >70%)
Chimeric edit (Indel frequency 1%-30%)
Monoallelic edit (Indel frequency >30% to 70%)
Lb
Lb-D156R
Lb-RV
Lb-RRV
Additional file 1: Fig. S4. Genotypes of T0 plants at 5 target sites of LbCas12a variants. A-E, Zygosity of T0 plants based on indel frequency at 5 target sites. Wild type (WT) denoted as empty rectangles, biallelic edit denoted as fully filled rectangles, monoallelic edit denoted as half-filled rectangles, chimeric edit denoted as doted rectangles.

## Slide 5
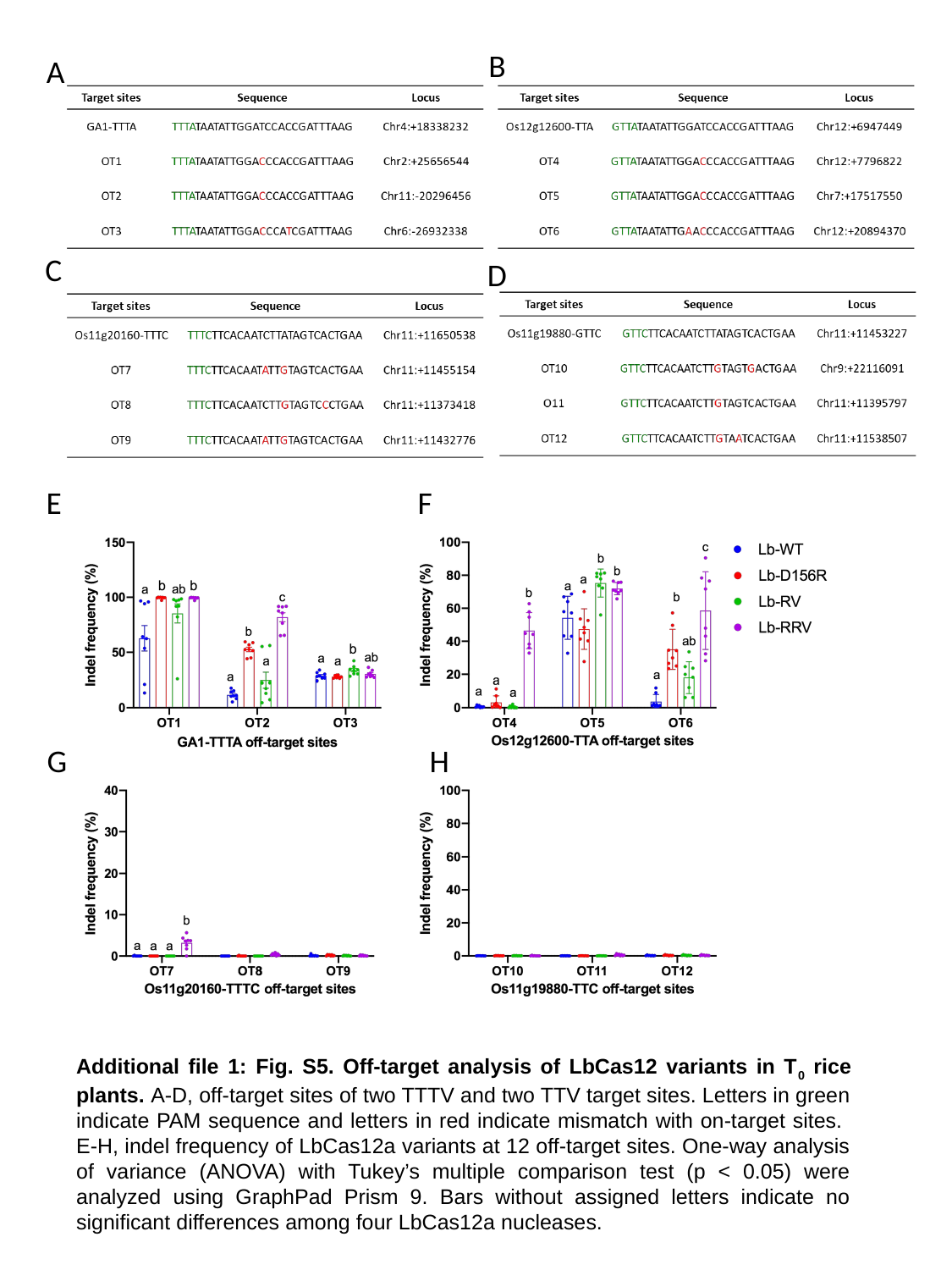

B
A
C
D
E
F
G
H
Additional file 1: Fig. S5. Off-target analysis of LbCas12 variants in T0 rice plants. A-D, off-target sites of two TTTV and two TTV target sites. Letters in green indicate PAM sequence and letters in red indicate mismatch with on-target sites. E-H, indel frequency of LbCas12a variants at 12 off-target sites. One-way analysis of variance (ANOVA) with Tukey’s multiple comparison test (p < 0.05) were analyzed using GraphPad Prism 9. Bars without assigned letters indicate no significant differences among four LbCas12a nucleases.
