# Supplementary material for: Boosting genome editing efficiency in human cells and plants with novel LbCas12a variants
Source: Genome Biol. 2023 Apr 30;24:102. doi: 10.1186/s13059-023-02929-6 (PMC10150537; doi:10.1186/s13059-023-02929-6)
Supplement: Supplementary file 2 — Additional file 2: Table S1. Positive mutations identified in the LbCas12a saturation screen. Table S2. T-DNA vectors used in this study. Table S3. Oligos used in this study. [file 13059_2023_2929_MOESM2_ESM.docx]

### Additional file 2: Table S1. Positive mutations identified in the LbCas12a saturation screen

| **Number** | **Mutation** | **Position** | **WT residue** | **Mutant residue** | **Average score** | **Standard deviation** |
| --- | --- | --- | --- | --- | --- | --- |
| **1** | K278D | 278 | K | D | 3.8 | 0.32 |
| **2** | E433P | 433 | E | P | 3.37 | 2.93 |
| **3** | V1209R | 1209 | V | R | 3.05 | 2.28 |
| **4** | K878A | 878 | K | A | 3.02 | 2.73 |
| **5** | A920P | 920 | A | P | 2.92 | 0.37 |
| **6** | G715R | 715 | G | R | 2.92 | 3.25 |
| **7** | N732L | 732 | N | L | 2.75 | 2.81 |
| **8** | I922R | 922 | I | R | 2.75 | 0.12 |
| **9** | S431T | 431 | S | T | 2.69 | 2.93 |
| **10** | F745T | 745 | F | T | 2.46 | 3.01 |
| **11** | N1081E | 1081 | N | E | 2.44 | 2.97 |
| **12** | V851F | 851 | V | F | 2.37 | 0.05 |
| **13** | F1052P | 1052 | F | P | 2.31 | 2.27 |
| **14** | S431G | 431 | S | G | 2.31 | 2.5 |
| **15** | D832R | 832 | D | R | 2.27 | 3.46 |
| **16** | N718Q | 718 | N | Q | 2.27 | 2.61 |
| **17** | A1222R | 1222 | A | R | 2.26 | 3.34 |
| **18** | V280R | 280 | V | R | 2.25 | 2.23 |
| **19** | K804I | 804 | K | I | 2.16 | 0.77 |
| **20** | R833V | 833 | R | V | 2.09 | 3.19 |
| **21** | M531S | 531 | M | S | 2.08 | 0.01 |
| **22** | A966P | 966 | A | P | 2.03 | 0.54 |
| **23** | E898N | 898 | E | N | 2.03 | 0.02 |
| **24** | L838D | 838 | L | D | 1.96 | 2.02 |
| **25** | S185Y | 185 | S | Y | 1.95 | 0.13 |
| **26** | G477T | 477 | G | T | 1.95 | 0.06 |
| **27** | D1023S | 1023 | D | S | 1.95 | 0.63 |
| **28** | H909M | 909 | H | M | 1.94 | 0.52 |
| **29** | A1169T | 1169 | A | T | 1.92 | 1.36 |
| **30** | N803F | 803 | N | F | 1.91 | 0.5 |
| **31** | Q906F | 906 | Q | F | 1.9 | 0.04 |
| **32** | E217Q | 217 | E | Q | 1.88 | 0.61 |
| **33** | L128E | 128 | L | E | 1.86 | 3.33 |
| **34** | H909K | 909 | H | K | 1.86 | 0.18 |
| **35** | G1009Q | 1009 | G | Q | 1.86 | 1.76 |
| **36** | L287A | 287 | L | A | 1.86 | 0.63 |
| **37** | I259S | 259 | I | S | 1.86 | 0.34 |
| **38** | E939A | 939 | E | A | 1.84 | 1.26 |
| **39** | K595P | 595 | K | P | 1.84 | 2.71 |
| **40** | K427Y | 427 | K | Y | 1.83 | 1.75 |
| **41** | G734L | 734 | G | L | 1.83 | 3.68 |
| **42** | G430L | 430 | G | L | 1.83 | 3.68 |
| **43** | K804F | 804 | K | F | 1.8 | 0.83 |
| **44** | K1227C | 1227 | K | C | 1.76 | 0.7 |
| **45** | K634T | 634 | K | T | 1.75 | 1.72 |
| **46** | H633P | 633 | H | P | 1.75 | 1.72 |
| **47** | Y57E | 57 | Y | E | 1.74 | 0.76 |
| **48** | R182V | 182 | R | V | 1.7 | 0.11 |
| **49** | A923R | 923 | A | R | 1.69 | 0.06 |
| **50** | R836A | 836 | R | A | 1.67 | 2.12 |
| **51** | N1082E | 1082 | N | E | 1.67 | 2.02 |
| **52** | A1194D | 1194 | A | D | 1.67 | 0.05 |
| **53** | S396D | 396 | S | D | 1.67 | 0.19 |
| **54** | K617S | 617 | K | S | 1.66 | 0.06 |
| **55** | Q613V | 613 | Q | V | 1.66 | 0.18 |
| **56** | E858V | 858 | E | V | 1.65 | 0 |
| **57** | N256R | 256 | N | R | 1.65 | 0.18 |
| **58** | K42E | 42 | K | E | 1.64 | 2.34 |
| **59** | I503E | 503 | I | E | 1.64 | 0.07 |
| **60** | G741L | 741 | G | L | 1.62 | 0.24 |
| **61** | I1021E | 1021 | I | E | 1.6 | 1.19 |
| **62** | G1103T | 1103 | G | T | 1.59 | 0.18 |
| **63** | T778P | 778 | T | P | 1.58 | 1.44 |
| **64** | R1138D | 1138 | R | D | 1.58 | 1.71 |
| **65** | V1083W | 1083 | V | W | 1.57 | 0.18 |
| **66** | K478R | 478 | K | R | 1.57 | 0.05 |
| **67** | P806D | 806 | P | D | 1.56 | 0.57 |
| **68** | K116I | 116 | K | I | 1.55 | 0.44 |
| **69** | L442F | 442 | L | F | 1.55 | 0.34 |
| **70** | L301F | 301 | L | F | 1.54 | 1.32 |
| **71** | A1046C | 1046 | A | C | 1.54 | 3.27 |
| **72** | F789N | 789 | F | N | 1.54 | 0.83 |
| **73** | D283M | 283 | D | M | 1.53 | 0.18 |
| **74** | F289D | 289 | F | D | 1.53 | 0.4 |
| **75** | F1084C | 1084 | F | C | 1.52 | 0.83 |
| **76** | N1178E | 1178 | N | E | 1.5 | 0.05 |
| **77** | G146R | 146 | G | R | 1.49 | 0.02 |
| **78** | R1165E | 1165 | R | E | 1.49 | 0.05 |
| **79** | A966N | 966 | A | N | 1.48 | 0.64 |
| **80** | T814G | 814 | T | G | 1.48 | 0.05 |
| **81** | L738Y | 738 | L | Y | 1.47 | 0.47 |
| **82** | G576R | 576 | G | R | 1.47 | 0.08 |
| **83** | A375W | 375 | A | W | 1.47 | 0.35 |
| **84** | R737V | 737 | R | V | 1.47 | 0.47 |
| **85** | N238F | 238 | N | F | 1.47 | 0.23 |
| **86** | V228T | 228 | V | T | 1.46 | 0.23 |
| **87** | V24H | 24 | V | H | 1.44 | 0.25 |
| **88** | S739K | 739 | S | K | 1.43 | 0.31 |
| **89** | Y11E | 11 | Y | E | 1.42 | 0.66 |
| **90** | S445N | 445 | S | N | 1.41 | 0.33 |
| **91** | K595R | 595 | K | R | 1.4 | 0.08 |
| **92** | T307W | 307 | T | W | 1.39 | 0.22 |
| **93** | W649A | 649 | W | A | 1.39 | 1.07 |
| **94** | N772R | 772 | N | R | 1.38 | 0.44 |
| **95** | V303W | 303 | V | W | 1.38 | 0.1 |
| **96** | K725H | 725 | K | H | 1.38 | 0.21 |
| **97** | S710I | 710 | S | I | 1.38 | 1.08 |
| **98** | Y583L | 583 | Y | L | 1.38 | 0.54 |
| **99** | S465D | 465 | S | D | 1.37 | 0.11 |
| **100** | I850S | 850 | I | S | 1.37 | 0.11 |
| **101** | E743T | 743 | E | T | 1.37 | 0 |
| **102** | N575P | 575 | N | P | 1.37 | 0.09 |
| **103** | A685D | 685 | A | D | 1.37 | 0.1 |
| **104** | S763H | 763 | S | H | 1.37 | 0.14 |
| **105** | E661W | 661 | E | W | 1.37 | 0.54 |
| **106** | A1022S | 1022 | A | S | 1.37 | 0.48 |
| **107** | S388G | 388 | S | G | 1.35 | 0.15 |
| **108** | Y290M | 290 | Y | M | 1.35 | 1.03 |
| **109** | E125R | 125 | E | R | 1.34 | 0.08 |
| **110** | Q1136A | 1136 | Q | A | 1.34 | 1.93 |
| **111** | T620M | 620 | T | M | 1.34 | 0.55 |
| **112** | E1202W | 1202 | E | W | 1.33 | 0.02 |
| **113** | R305T | 305 | R | T | 1.33 | 1.2 |
| **114** | K634I | 634 | K | I | 1.33 | 1.12 |
| **115** | V938G | 938 | V | G | 1.33 | 0.44 |
| **116** | N861I | 861 | N | I | 1.32 | 0.03 |
| **117** | C175D | 175 | C | D | 1.32 | 0.44 |
| **118** | K265V | 265 | K | V | 1.32 | 0.54 |
| **119** | A1022I | 1022 | A | I | 1.32 | 0.43 |
| **120** | K464L | 464 | K | L | 1.32 | 0.55 |
| **121** | S1024E | 1024 | S | E | 1.31 | 0.31 |
| **122** | N861V | 861 | N | V | 1.31 | 0.16 |
| **123** | P799S | 799 | P | S | 1.3 | 0.05 |
| **124** | I860R | 860 | I | R | 1.29 | 0.28 |
| **125** | S750V | 750 | S | V | 1.29 | 0.05 |
| **126** | F983W | 983 | F | W | 1.27 | 1.03 |
| **127** | D384W | 384 | D | W | 1.27 | 0.06 |
| **128** | C805F | 805 | C | F | 1.27 | 0.52 |
| **129** | S282W | 282 | S | W | 1.27 | 1.52 |
| **130** | V844N | 844 | V | N | 1.27 | 0.93 |
| **131** | N864S | 864 | N | S | 1.27 | 0.05 |
| **132** | E835A | 835 | E | A | 1.26 | 0.05 |
| **133** | A920T | 920 | A | T | 1.25 | 1.43 |
| **134** | F639C | 639 | F | C | 1.25 | 0.06 |
| **135** | K953S | 953 | K | S | 1.25 | 0.41 |
| **136** | D641I | 641 | D | I | 1.25 | 0.05 |
| **137** | A1122Q | 1122 | A | Q | 1.25 | 0.31 |
| **138** | L547H | 547 | L | H | 1.25 | 0.07 |
| **139** | P342L | 342 | P | L | 1.25 | 0.09 |
| **140** | F709K | 709 | F | K | 1.25 | 0.92 |
| **141** | K584E | 584 | K | E | 1.25 | 0.42 |
| **142** | Y262L | 262 | Y | L | 1.24 | 0.12 |
| **143** | D1207F | 1207 | D | F | 1.24 | 0.11 |
| **144** | M1134G | 1134 | M | G | 1.23 | 1.73 |
| **145** | V596K | 596 | V | K | 1.23 | 0.43 |
| **146** | G576M | 576 | G | M | 1.22 | 0.43 |
| **147** | L1133T | 1133 | L | T | 1.22 | 0.16 |
| **148** | K945L | 945 | K | L | 1.22 | 0.17 |
| **149** | M603Y | 603 | M | Y | 1.22 | 0.21 |
| **150** | I1211D | 1211 | I | D | 1.21 | 1.39 |
| **151** | S394Q | 394 | S | Q | 1.21 | 0.14 |
| **152** | E201I | 201 | E | I | 1.21 | 0.05 |
| **153** | S642M | 642 | S | M | 1.2 | 0.88 |
| **154** | M1137Q | 1137 | M | Q | 1.2 | 0.05 |
| **155** | Q888S | 888 | Q | S | 1.2 | 0.83 |
| **156** | R56V | 56 | R | V | 1.2 | 0.4 |
| **157** | L80D | 80 | L | D | 1.2 | 0.84 |
| **158** | S650A | 650 | S | A | 1.2 | 1.36 |
| **159** | L13C | 13 | L | C | 1.2 | 0.33 |
| **160** | Q1170D | 1170 | Q | D | 1.19 | 0.15 |
| **161** | F1198Q | 1198 | F | Q | 1.19 | 0.93 |
| **162** | F1198G | 1198 | F | G | 1.19 | 0.93 |
| **163** | F225R | 225 | F | R | 1.19 | 0.24 |
| **164** | K478W | 478 | K | W | 1.18 | 0.08 |
| **165** | I860S | 860 | I | S | 1.17 | 0.09 |
| **166** | E5Y | 5 | E | Y | 1.17 | 0.22 |
| **167** | A544T | 544 | A | T | 1.17 | 0.22 |
| **168** | N933E | 933 | N | E | 1.16 | 0.31 |
| **169** | A1129G | 1129 | A | G | 1.16 | 0.05 |
| **170** | H733K | 733 | H | K | 1.16 | 0.31 |
| **171** | D283R | 283 | D | R | 1.16 | 1.18 |
| **172** | E125S | 125 | E | S | 1.16 | 0.13 |
| **173** | N731H | 731 | N | H | 1.15 | 1.06 |
| **174** | A1129R | 1129 | A | R | 1.15 | 1.81 |
| **175** | R305D | 305 | R | D | 1.14 | 0.53 |
| **176** | K478F | 478 | K | F | 1.14 | 0.07 |
| **177** | D926Q | 926 | D | Q | 1.14 | 0.02 |
| **178** | Q231S | 231 | Q | S | 1.13 | 0.53 |
| **179** | L442C | 442 | L | C | 1.13 | 0.21 |
| **180** | L210Y | 210 | L | Y | 1.13 | 0.83 |
| **181** | S317M | 317 | S | M | 1.13 | 0.84 |
| **182** | F289Q | 289 | F | Q | 1.13 | 0.67 |
| **183** | R867Q | 867 | R | Q | 1.13 | 0.05 |
| **184** | E223L | 223 | E | L | 1.13 | 0.25 |
| **185** | E93S | 93 | E | S | 1.13 | 0.15 |
| **186** | V453R | 453 | V | R | 1.12 | 0.15 |
| **187** | L1091A | 1091 | L | A | 1.12 | 0.73 |
| **188** | L408D | 408 | L | D | 1.12 | 1.69 |
| **189** | A451K | 451 | A | K | 1.12 | 0.15 |
| **190** | G1196I | 1196 | G | I | 1.12 | 0.83 |
| **191** | K707Y | 707 | K | Y | 1.12 | 0.26 |
| **192** | F162H | 162 | F | H | 1.12 | 0.15 |
| **193** | H67G | 67 | H | G | 1.12 | 0.73 |
| **194** | E443N | 443 | E | N | 1.11 | 0.72 |
| **195** | K879D | 879 | K | D | 1.11 | 0.72 |
| **196** | Q529I | 529 | Q | I | 1.11 | 0.06 |
| **197** | H370G | 370 | H | G | 1.11 | 0.09 |
| **198** | K622P | 622 | K | P | 1.11 | 0.83 |
| **199** | D64Q | 64 | D | Q | 1.1 | 0.16 |
| **200** | F173T | 173 | F | T | 1.1 | 0.12 |
| **201** | C1090K | 1090 | C | K | 1.1 | 0.17 |
| **202** | I951S | 951 | I | S | 1.09 | 0.05 |
| **203** | V1147Y | 1147 | V | Y | 1.09 | 0.18 |
| **204** | T246V | 246 | T | V | 1.08 | 0.22 |
| **205** | D198Q | 198 | D | Q | 1.08 | 0.67 |
| **206** | K774P | 774 | K | P | 1.08 | 1.21 |
| **207** | Y11Q | 11 | Y | Q | 1.08 | 0.17 |
| **208** | T778G | 778 | T | G | 1.07 | 2.27 |
| **209** | G111Y | 111 | G | Y | 1.07 | 0.06 |
| **210** | K1210Y | 1210 | K | Y | 1.07 | 0.16 |
| **211** | E683I | 683 | E | I | 1.07 | 0.32 |
| **212** | R82E | 82 | R | E | 1.06 | 0.41 |
| **213** | E125K | 125 | E | K | 1.06 | 0.04145R |
| **214** | S713E | 713 | S | E | 1.06 | 0.08 |
| **215** | H759G | 759 | H | G | 1.06 | 0.6 |
| **216** | E90F | 90 | E | F | 1.06 | 0.06 |
| **217** | K478L | 478 | K | L | 1.06 | 0.1 |
| **218** | K478Y | 478 | K | Y | 1.06 | 0.07 |
| **219** | A766Y | 766 | A | Y | 1.06 | 0.6 |
| **220** | E125N | 125 | E | N | 1.05 | 0.04 |
| **221** | A1194V | 1194 | A | V | 1.05 | 0.06 |
| **222** | K391F | 391 | K | F | 1.05 | 0.27 |
| **223** | K464A | 464 | K | A | 1.05 | 0.05 |
| **224** | A923G | 923 | A | G | 1.05 | 0.24 |
| **225** | Q399V | 399 | Q | V | 1.04 | 0.7 |
| **226** | R1144H | 1144 | R | H | 1.04 | 0.29 |
| **227** | N1100M | 1100 | N | M | 1.04 | 0.7 |
| **228** | F789T | 789 | F | T | 1.04 | 0.39 |
| **229** | R35G | 35 | R | G | 1.04 | 0.28 |
| **230** | W890Q | 890 | W | Q | 1.04 | 0.7 |
| **231** | A21N | 21 | A | N | 1.04 | 0.03 |
| **232** | K448Y | 448 | K | Y | 1.03 | 0.7 |
| **233** | D461L | 461 | D | L | 1.03 | 0.59 |
| **234** | T87P | 87 | T | P | 1.03 | 0.02 |
| **235** | E433H | 433 | E | H | 1.03 | 0.7 |
| **236** | K457S | 457 | K | S | 1.03 | 1.68 |
| **237** | W602Q | 602 | W | Q | 1.03 | 0.4 |
| **238** | S394C | 394 | S | C | 1.03 | 0.15 |
| **239** | S485D | 485 | S | D | 1.02 | 0.29 |
| **240** | K804Y | 804 | K | Y | 1.02 | 0.89 |
| **241** | I196K | 196 | I | K | 1.02 | 0.05 |
| **242** | K1079H | 1079 | K | H | 1.02 | 0.7 |
| **243** | L876S | 876 | L | S | 1.02 | 1.24 |
| **244** | E755S | 755 | E | S | 1.02 | 0.05 |
| **245** | I259W | 259 | I | W | 1.02 | 0.7 |
| **246** | Y1059E | 1059 | Y | E | 1.01 | 0.39 |
| **247** | T660C | 660 | T | C | 1.01 | 0.17 |
| **248** | G1196C | 1196 | G | C | 1.01 | 1.41 |
| **249** | I860E | 860 | I | E | 1.01 | 0.31 |
| **250** | A239T | 239 | A | T | 1.01 | 0.18 |
| **251** | E125A | 125 | E | A | 1 | 0.06 |
| **252** | N861T | 861 | N | T | 0.99 | 0.12 |
| **253** | I976A | 976 | I | A | 0.99 | 0.52 |
| **254** | R1073H | 1073 | R | H | 0.99 | 0.1 |
| **255** | D329F | 329 | D | F | 0.99 | 0.54 |
| **256** | K568F | 568 | K | F | 0.99 | 0.16 |
| **257** | T1142Y | 1142 | T | Y | 0.99 | 0.05 |
| **258** | R482T | 482 | R | T | 0.98 | 0.43 |
| **259** | A21F | 21 | A | F | 0.98 | 0.62 |
| **260** | K897H | 897 | K | H | 0.98 | 0.05 |
| **261** | S1020D | 1020 | S | D | 0.98 | 0.01 |
| **262** | D437I | 437 | D | I | 0.98 | 1.5 |
| **263** | K945V | 945 | K | V | 0.98 | 0.12 |
| **264** | F1099S | 1099 | F | S | 0.98 | 0.15 |
| **265** | S1020E | 1020 | S | E | 0.98 | 0.12 |
| **266** | E433Q | 433 | E | Q | 0.98 | 0.52 |
| **267** | I138Q | 138 | I | Q | 0.98 | 0.21 |
| **268** | E313C | 313 | E | C | 0.98 | 0.63 |
| **269** | N1051G | 1051 | N | G | 0.97 | 0.05 |
| **270** | W1086A | 1086 | W | A | 0.97 | 0.05 |
| **271** | M626D | 626 | M | D | 0.97 | 0.06 |
| **272** | N327E | 327 | N | E | 0.97 | 0.18 |
| **273** | C1090M | 1090 | C | M | 0.97 | 0.05 |
| **274** | I893Q | 893 | I | Q | 0.97 | 0.05 |
| **275** | S462E | 462 | S | E | 0.97 | 1.02 |
| **276** | K374P | 374 | K | P | 0.97 | 0.22 |
| **277** | G291T | 291 | G | T | 0.97 | 0.12 |
| **278** | I850Q | 850 | I | Q | 0.97 | 0.19 |
| **279** | R508G | 508 | R | G | 0.97 | 0.07 |
| **280** | H733T | 733 | H | T | 0.97 | 1.24 |
| **281** | Q1197R | 1197 | Q | R | 0.96 | 0.09 |
| **282** | E95K | 95 | E | K | 0.96 | 0.02 |
| **283** | S296L | 296 | S | L | 0.96 | 0.3 |
| **284** | A801L | 801 | A | L | 0.96 | 0.18 |
| **285** | F466A | 466 | F | A | 0.96 | 1.48 |
| **286** | K269Y | 269 | K | Y | 0.96 | 0.19 |
| **287** | G576K | 576 | G | K | 0.95 | 0.3 |
| **288** | E880T | 880 | E | T | 0.95 | 0.6 |
| **289** | Q1223S | 1223 | Q | S | 0.95 | 0.1 |
| **290** | E247P | 247 | E | P | 0.95 | 0.29 |
| **291** | K774E | 774 | K | E | 0.95 | 0.86 |
| **292** | R1054F | 1054 | R | F | 0.95 | 0.38 |
| **293** | S1157M | 1157 | S | M | 0.95 | 0.01 |
| **294** | K568W | 568 | K | W | 0.95 | 0.06 |
| **295** | K1025G | 1025 | K | G | 0.94 | 0.14 |
| **296** | K478C | 478 | K | C | 0.94 | 0.1 |
| **297** | V410P | 410 | V | P | 0.94 | 0.3 |
| **298** | K1121D | 1121 | K | D | 0.94 | 0.1 |
| **299** | E285Y | 285 | E | Y | 0.94 | 0.17 |
| **300** | V428H | 428 | V | H | 0.94 | 1.52 |
| **301** | K478M | 478 | K | M | 0.93 | 0.11 |
| **302** | D1148M | 1148 | D | M | 0.93 | 0.44 |
| **303** | L914K | 914 | L | K | 0.93 | 0.18 |
| **304** | Y995L | 995 | Y | L | 0.93 | 0.05 |
| **305** | K881T | 881 | K | T | 0.93 | 3.38 |
| **306** | K120C | 120 | K | C | 0.93 | 0.54 |
| **307** | A1173D | 1173 | A | D | 0.93 | 0.11 |
| **308** | K1121E | 1121 | K | E | 0.92 | 0.08 |
| **309** | G846L | 846 | G | L | 0.92 | 0.44 |
| **310** | K478A | 478 | K | A | 0.92 | 0.08 |
| **311** | G968W | 968 | G | W | 0.92 | 0.54 |
| **312** | K1121C | 1121 | K | C | 0.92 | 0.27 |
| **313** | L140A | 140 | L | A | 0.92 | 0.44 |
| **314** | E947N | 947 | E | N | 0.92 | 0.54 |
| **315** | S934Q | 934 | S | Q | 0.92 | 0.44 |
| **316** | D771P | 771 | D | P | 0.92 | 1.06 |
| **317** | R935M | 935 | R | M | 0.92 | 0.44 |
| **318** | G624R | 624 | G | R | 0.92 | 0.55 |
| **319** | E939R | 939 | E | R | 0.92 | 0.54 |
| **320** | Y606F | 606 | Y | F | 0.92 | 0.07 |
| **321** | E88S | 88 | E | S | 0.92 | 0.04 |
| **322** | S599V | 599 | S | V | 0.92 | 0.43 |
| **323** | V441Q | 441 | V | Q | 0.92 | 1.42 |
| **324** | E1039T | 1039 | E | T | 0.92 | 0.14 |
| **325** | F473V | 473 | F | V | 0.92 | 0.06 |
| **326** | A604H | 604 | A | H | 0.92 | 0.43 |
| **327** | K326Y | 326 | K | Y | 0.92 | 0.55 |
| **328** | S855P | 855 | S | P | 0.92 | 0.54 |
| **329** | C632T | 632 | C | T | 0.92 | 0.55 |
| **330** | S686F | 686 | S | F | 0.91 | 1.41 |
| **331** | K478P | 478 | K | P | 0.91 | 0.04 |
| **332** | F597K | 597 | F | K | 0.91 | 0.56 |
| **333** | D156K | 156 | D | K | 0.91 | 0.01 |
| **334** | E852N | 852 | E | N | 0.91 | 0.67 |
| **335** | R82N | 82 | R | N | 0.91 | 0.1 |
| **336** | Q1170T | 1170 | Q | T | 0.91 | 1.25 |
| **337** | S68K | 68 | S | K | 0.91 | 0.04 |
| **338** | G740P | 740 | G | P | 0.91 | 1.52 |
| **339** | S642G | 642 | S | G | 0.91 | 0.24 |
| **340** | K1080T | 1080 | K | T | 0.91 | 3.08 |
| **341** | E683C | 683 | E | C | 0.91 | 0.43 |
| **342** | Y262P | 262 | Y | P | 0.91 | 0.44 |
| **343** | A556V | 556 | A | V | 0.9 | 1.13 |
| **344** | P1153I | 1153 | P | I | 0.9 | 0.05 |
| **345** | D495G | 495 | D | G | 0.9 | 0.05 |
| **346** | T152K | 152 | T | K | 0.9 | 0.03 |
| **347** | K269F | 269 | K | F | 0.9 | 0.1 |
| **348** | L1047K | 1047 | L | K | 0.89 | 0.1 |
| **349** | K1025P | 1025 | K | P | 0.89 | 0.06 |
| **350** | F474P | 474 | F | P | 0.89 | 0.19 |
| **351** | K561C | 561 | K | C | 0.89 | 0.55 |
| **352** | P799V | 799 | P | V | 0.89 | 0.02839F |
| **353** | K326G | 326 | K | G | 0.89 | 0.11 |
| **354** | A1194C | 1194 | A | C | 0.89 | 0.9 |
| **355** | N731G | 731 | N | G | 0.88 | 0.08 |
| **356** | I841G | 841 | I | G | 0.88 | 0.13 |
| **357** | P342F | 342 | P | F | 0.88 | 0.2 |
| **358** | I860T | 860 | I | T | 0.88 | 0.1 |
| **359** | Y646H | 646 | Y | H | 0.87 | 0.01 |
| **360** | P799Y | 799 | P | Y | 0.87 | 0.18 |
| **361** | S1024D | 1024 | S | D | 0.87 | 0.08 |
| **362** | K135P | 135 | K | P | 0.87 | 0.08 |
| **363** | T480D | 480 | T | D | 0.87 | 1.04 |
| **364** | L414G | 414 | L | G | 0.87 | 0.23 |
| **365** | A1057M | 1057 | A | M | 0.87 | 0.03 |
| **366** | I392D | 392 | I | D | 0.87 | 0.45 |
| **367** | K478D | 478 | K | D | 0.86 | 0.01 |
| **368** | F109R | 109 | F | R | 0.86 | 0.31 |
| **369** | P271S | 271 | P | S | 0.86 | 0.02 |
| **370** | N706F | 706 | N | F | 0.86 | 1.32 |
| **371** | A1169E | 1169 | A | E | 0.86 | 0.13 |
| **372** | D384L | 384 | D | L | 0.86 | 0.23 |
| **373** | D729C | 729 | D | C | 0.86 | 1.32 |
| **374** | S1152E | 1152 | S | E | 0.85 | 1.33 |
| **375** | F931A | 931 | F | A | 0.85 | 0.45 |
| **376** | K208S | 208 | K | S | 0.85 | 0.16 |
| **377** | D1203W | 1203 | D | W | 0.85 | 0.35 |
| **378** | K1015Y | 1015 | K | Y | 0.84 | 0.14 |
| **379** | H873W | 873 | H | W | 0.84 | 0.21 |
| **380** | R182D | 182 | R | D | 0.84 | 0.12 |
| **381** | P608N | 608 | P | N | 0.84 | 0.84 |
| **382** | E285W | 285 | E | W | 0.84 | 0.45 |
| **383** | F19D | 19 | F | D | 0.83 | 0.82 |
| **384** | K206A | 206 | K | A | 0.83 | 0.31 |
| **385** | K478Q | 478 | K | Q | 0.83 | 0.1 |
| **386** | E1202V | 1202 | E | V | 0.83 | 0.1 |
| **387** | G488T | 488 | G | T | 0.83 | 0.16 |
| **388** | S1024Y | 1024 | S | Y | 0.83 | 0.26 |
| **389** | I809C | 809 | I | C | 0.83 | 0.72 |
| **390** | D64T | 64 | D | T | 0.83 | 0.44 |
| **391** | N356W | 356 | N | W | 0.83 | 0.02 |
| **392** | D156Q | 156 | D | Q | 0.83 | 0.08 |
| **393** | I860Q | 860 | I | Q | 0.83 | 0.13 |
| **394** | L94Q | 94 | L | Q | 0.83 | 0.12 |
| **395** | K932Q | 932 | K | Q | 0.82 | 0.31 |
| **396** | N861M | 861 | N | M | 0.82 | 0.15 |
| **397** | E484L | 484 | E | L | 0.82 | 0.07 |
| **398** | E125V | 125 | E | V | 0.82 | 0.2 |
| **399** | G1069E | 1069 | G | E | 0.82 | 0.31 |
| **400** | F81E | 81 | F | E | 0.82 | 0.08 |
| **401** | K478S | 478 | K | S | 0.82 | 0.12 |
| **402** | I976Q | 976 | I | Q | 0.82 | 0.29 |
| **403** | S394L | 394 | S | L | 0.82 | 0.02 |
| **404** | E1220K | 1220 | E | K | 0.82 | 0.25 |
| **405** | G1143W | 1143 | G | W | 0.82 | 0.7 |
| **406** | A966C | 966 | A | C | 0.82 | 0.33 |
| **407** | A406R | 406 | A | R | 0.82 | 0.69 |
| **408** | K1079P | 1079 | K | P | 0.81 | 2.78 |
| **409** | K910C | 910 | K | C | 0.81 | 3.35 |
| **410** | I612R | 612 | I | R | 0.81 | 0.17 |
| **411** | N179F | 179 | N | F | 0.81 | 0.23 |
| **412** | E125C | 125 | E | C | 0.81 | 0.07 |
| **413** | G111Q | 111 | G | Q | 0.81 | 0.17 |
| **414** | L779W | 779 | L | W | 0.81 | 0.06 |
| **415** | D501V | 501 | D | V | 0.8 | 0.67 |
| **416** | L839F | 839 | L | F | 0.8 | 0.18 |
|  |  |  |  |  |  |  |
| **417** | F1198A | 1198 | F | A | 0.8 | 0.32 |
| **418** | L255W | 255 | L | W | 0.8 | 0.18 |
| **419** | E204S | 204 | E | S | 0.8 | 0.05 |
| **420** | F81T | 81 | F | T | 0.8 | 0.24 |
| **421** | E125T | 125 | E | T | 0.8 | 0.07 |
| **422** | S684L | 684 | S | L | 0.8 | 0.22 |
| **423** | K253H | 253 | K | H | 0.8 | 0.06 |
| **424** | R867H | 867 | R | H | 0.79 | 0.05 |
| **425** | E433W | 433 | E | W | 0.79 | 0.05 |
| **426** | S790F | 790 | S | F | 0.79 | 0.55 |
| **427** | G114Y | 114 | G | Y | 0.79 | 0.23 |
| **428** | P806M | 806 | P | M | 0.79 | 0.06 |
| **429** | A404N | 404 | A | N | 0.79 | 0.24 |
| **430** | Y294E | 294 | Y | E | 0.79 | 0.05 |
| **431** | Q264S | 264 | Q | S | 0.79 | 0.05 |
| **432** | S185L | 185 | S | L | 0.79 | 0.06 |
| **433** | N306C | 306 | N | C | 0.79 | 0.35 |
| **434** | Q399D | 399 | Q | D | 0.79 | 0.24 |
| **435** | Y426Q | 426 | Y | Q | 0.79 | 0.08 |
| **436** | L435I | 435 | L | I | 0.79 | 0.27 |
| **437** | N861L | 861 | N | L | 0.79 | 0.02 |
| **438** | V942I | 942 | V | I | 0.79 | 0.34 |
| **439** | E981V | 981 | E | V | 0.79 | 0.03 |
| **440** | K698C | 698 | K | C | 0.79 | 0.23 |
| **441** | R1138T | 1138 | R | T | 0.78 | 0.24 |
| **442** | L301C | 301 | L | C | 0.78 | 0.61 |
| **443** | N327Y | 327 | N | Y | 0.78 | 0.23 |
| **444** | L899K | 899 | L | K | 0.78 | 0.23 |
| **445** | G991T | 991 | G | T | 0.78 | 0.23 |
| **446** | V851H | 851 | V | H | 0.78 | 0.34 |
| **447** | G1103K | 1103 | G | K | 0.78 | 0.34 |
| **448** | G1009L | 1009 | G | L | 0.78 | 0.23 |
| **449** | S317P | 317 | S | P | 0.78 | 0.23 |
| **450** | S934A | 934 | S | A | 0.78 | 0.34 |
| **451** | E852K | 852 | E | K | 0.78 | 0.23 |
| **452** | A108Y | 108 | A | Y | 0.78 | 0.34 |
| **453** | I860K | 860 | I | K | 0.78 | 0.15 |
| **454** | E939V | 939 | E | V | 0.78 | 0.24 |
| **455** | W999T | 999 | W | T | 0.78 | 1.32 |
| **456** | D188M | 188 | D | M | 0.78 | 0.24 |
| **457** | S1152D | 1152 | S | D | 0.78 | 1.22 |
| **458** | G335N | 335 | G | N | 0.78 | 0.23 |
| **459** | R158A | 158 | R | A | 0.78 | 0.23 |
| **460** | S905Q | 905 | S | Q | 0.77 | 0.23 |
| **461** | N1186E | 1186 | N | E | 0.77 | 0.33 |
| **462** | K979I | 979 | K | I | 0.77 | 0.34 |
| **463** | H633I | 633 | H | I | 0.77 | 0.34 |
| **464** | E795Q | 795 | E | Q | 0.77 | 0.09 |
| **465** | V38E | 38 | V | E | 0.77 | 0.09 |
| **466** | Y616V | 616 | Y | V | 0.77 | 0.34 |
| **467** | I809N | 809 | I | N | 0.77 | 0.23 |
| **468** | G488K | 488 | G | K | 0.77 | 0.23 |
| **469** | A1194R | 1194 | A | R | 0.77 | 1.31 |
| **470** | A364W | 364 | A | W | 0.77 | 0.23 |
| **471** | K1199F | 1199 | K | F | 0.77 | 0.74 |
| **472** | F227N | 227 | F | N | 0.77 | 0.66 |
| **473** | N1166E | 1166 | N | E | 0.77 | 0.15 |
| **474** | V511H | 511 | V | H | 0.77 | 0.22 |
| **475** | E292N | 292 | E | N | 0.77 | 0.34 |
| **476** | V491D | 491 | V | D | 0.77 | 0.1 |
| **477** | R1071V | 1071 | R | V | 0.77 | 0.34 |
| **478** | G477P | 477 | G | P | 0.76 | 0.02 |
| **479** | K688D | 688 | K | D | 0.76 | 0.1 |
| **480** | A506L | 506 | A | L | 0.76 | 1.33 |
| **481** | K310I | 310 | K | I | 0.76 | 0.34 |
| **482** | S288Q | 288 | S | Q | 0.76 | 2.07 |
| **483** | S394V | 394 | S | V | 0.76 | 0.08 |
| **484** | V673W | 673 | V | W | 0.76 | 0.23 |
| **485** | D641Q | 641 | D | Q | 0.76 | 0.23 |
| **486** | F227A | 227 | F | A | 0.76 | 0.33 |
| **487** | Y646W | 646 | Y | W | 0.76 | 0.12 |
| **488** | T977I | 977 | T | I | 0.76 | 0.21 |
| **489** | D156M | 156 | D | M | 0.76 | 0.2 |
| **490** | A1022R | 1022 | A | R | 0.76 | 0.98 |
| **491** | D1207S | 1207 | D | S | 0.76 | 0.01 |
| **492** | E947R | 947 | E | R | 0.76 | 0.07 |
| **493** | I841A | 841 | I | A | 0.76 | 0.03 |
| **494** | F466R | 466 | F | R | 0.76 | 0.14 |
| **495** | K20R | 20 | K | R | 0.75 | 0.12 |
| **496** | Y1106Q | 1106 | Y | Q | 0.75 | 1.22 |
| **497** | N1100D | 1100 | N | D | 0.75 | 0.22 |
| **498** | T814K | 814 | T | K | 0.75 | 0.1 |
| **499** | S286Q | 286 | S | Q | 0.75 | 1.19 |
| **500** | T8A | 8 | T | A | 0.75 | 0.26 |
| **501** | E939K | 939 | E | K | 0.75 | 0.29 |
| **502** | A761Y | 761 | A | Y | 0.75 | 0.02 |
| **503** | Q1170F | 1170 | Q | F | 0.75 | 0.02 |
| **504** | Y974G | 974 | Y | G | 0.75 | 0.14 |
| **505** | A1129Q | 1129 | A | Q | 0.75 | 0.25 |
| **506** | D665N | 665 | D | N | 0.74 | 0.09 |
| **507** | D559A | 559 | D | A | 0.74 | 0.22 |
| **508** | K1025C | 1025 | K | C | 0.74 | 0.04 |
| **509** | K561I | 561 | K | I | 0.74 | 0.22 |
| **510** | E1044L | 1044 | E | L | 0.74 | 0.13 |
| **511** | M1128S | 1128 | M | S | 0.74 | 0.06 |
| **512** | I62A | 62 | I | A | 0.74 | 0.33 |
| **513** | R482D | 482 | R | D | 0.74 | 0.05 |
| **514** | K687G | 687 | K | G | 0.74 | 0.23 |
| **515** | K879L | 879 | K | L | 0.74 | 0.14 |
| **516** | L4N | 4 | L | N | 0.74 | 0.31 |
| **517** | K478G | 478 | K | G | 0.74 | 0.07 |
| **518** | E88A | 88 | E | A | 0.74 | 0.18 |
| **519** | R182L | 182 | R | L | 0.74 | 0.04 |
| **520** | R1165L | 1165 | R | L | 0.74 | 1.26 |
| **521** | V1154P | 1154 | V | P | 0.74 | 0.05 |
| **522** | A685M | 685 | A | M | 0.74 | 0.03 |
| **523** | L875H | 875 | L | H | 0.73 | 1.26 |
| **524** | G475I | 475 | G | I | 0.73 | 0.08 |
| **525** | N1081Q | 1081 | N | Q | 0.73 | 0.17 |
| **526** | F655D | 655 | F | D | 0.73 | 1.32 |
| **527** | K1015F | 1015 | K | F | 0.73 | 0.06 |
| **528** | E125M | 125 | E | M | 0.73 | 0.2 |
| **529** | F337T | 337 | F | T | 0.73 | 0.1 |
| **530** | Y549G | 549 | Y | G | 0.73 | 0.09 |
| **531** | R182P | 182 | R | P | 0.73 | 0.07 |
| **532** | S409N | 409 | S | N | 0.73 | 0.26 |
| **533** | K937V | 937 | K | V | 0.73 | 0.25 |
| **534** | I344G | 344 | I | G | 0.73 | 0.09 |
| **535** | K1015D | 1015 | K | D | 0.72 | 0.08 |
| **536** | T778R | 778 | T | R | 0.72 | 0.34 |
| **537** | F19E | 19 | F | E | 0.72 | 0.32 |
| **538** | N577E | 577 | N | E | 0.72 | 0.16 |
| **539** | K478V | 478 | K | V | 0.72 | 0.06 |
| **540** | T378G | 378 | T | G | 0.72 | 0.66 |
| **541** | K478T | 478 | K | T | 0.72 | 0.12 |
| **542** | D654M | 654 | D | M | 0.72 | 1.15 |
| **543** | R1033G | 1033 | R | G | 0.72 | 0.26 |
| **544** | E858Y | 858 | E | Y | 0.72 | 0.08 |
| **545** | I866R | 866 | I | R | 0.72 | 0.65 |
| **546** | E95S | 95 | E | S | 0.72 | 0.03 |
| **547** | K434T | 434 | K | T | 0.71 | 2.2 |
| **548** | F81D | 81 | F | D | 0.71 | 0.06 |
| **549** | M456L | 456 | M | L | 0.71 | 0.09 |
| **550** | S780V | 780 | S | V | 0.71 | 0.32 |
| **551** | I369D | 369 | I | D | 0.71 | 0.24 |
| **552** | V1083S | 1083 | V | S | 0.71 | 0.19 |
| **553** | L555I | 555 | L | I | 0.71 | 0.06 |
| **554** | K1096Q | 1096 | K | Q | 0.7 | 0.65 |
| **555** | R482Q | 482 | R | Q | 0.7 | 0.01 |
| **556** | A742W | 742 | A | W | 0.7 | 0.55 |
| **557** | K1121I | 1121 | K | I | 0.7 | 0.08 |
| **558** | S644R | 644 | S | R | 0.7 | 0.7 |
| **559** | K1096F | 1096 | K | F | 0.7 | 0.36 |
| **560** | N306E | 306 | N | E | 0.7 | 0.36 |
| **561** | N306G | 306 | N | G | 0.7 | 1.2 |
| **562** | Q401V | 401 | Q | V | 0.7 | 0.13 |
| **563** | G393P | 393 | G | P | 0.7 | 0.21 |
| **564** | F197H | 197 | F | H | 0.7 | 0.1 |
| **565** | K948E | 948 | K | E | 0.7 | 0.12 |
| **566** | T346Q | 346 | T | Q | 0.69 | 1.2 |
| **567** | N263W | 263 | N | W | 0.69 | 0.05 |
| **568** | D156T | 156 | D | T | 0.69 | 0.05 |
| **569** | G293P | 293 | G | P | 0.69 | 0.1 |
| **570** | D782M | 782 | D | M | 0.69 | 0.06 |
| **571** | F119K | 119 | F | K | 0.69 | 1.18 |
| **572** | K415A | 415 | K | A | 0.69 | 1.19 |
| **573** | R1138Q | 1138 | R | Q | 0.69 | 0.21 |
| **574** | K1101E | 1101 | K | E | 0.69 | 1.09 |
| **575** | Y1018R | 1018 | Y | R | 0.69 | 1.24 |
| **576** | N1105S | 1105 | N | S | 0.69 | 0.2 |
| **577** | A375Y | 375 | A | Y | 0.69 | 0.1 |
| **578** | S312Y | 312 | S | Y | 0.69 | 1.2 |
| **579** | R182Q | 182 | R | Q | 0.69 | 0.05 |
| **580** | L446K | 446 | L | K | 0.69 | 0.21 |
| **581** | I205E | 205 | I | E | 0.69 | 1.09 |
| **582** | V300K | 300 | V | K | 0.69 | 0.21 |
| **583** | G1103A | 1103 | G | A | 0.69 | 0.11 |
| **584** | V921M | 921 | V | M | 0.69 | 1.19 |
| **585** | K208A | 208 | K | A | 0.69 | 0.21 |
| **586** | K274G | 274 | K | G | 0.69 | 0.09 |
| **587** | W1063N | 1063 | W | N | 0.69 | 0.06 |
| **588** | E852T | 852 | E | T | 0.68 | 0.17 |
| **589** | S609D | 609 | S | D | 0.68 | 0.09 |
| **590** | T295V | 295 | T | V | 0.68 | 0.22 |
| **591** | T1019E | 1019 | T | E | 0.68 | 0.07 |
| **592** | L586T | 586 | L | T | 0.68 | 0.22 |
| **593** | M187D | 187 | M | D | 0.68 | 0.04 |
| **594** | Q567C | 567 | Q | C | 0.68 | 0.03 |
| **595** | E298A | 298 | E | A | 0.68 | 1.19 |
| **596** | E925R | 925 | E | R | 0.68 | 0.4 |
| **597** | P342E | 342 | P | E | 0.68 | 0.12 |
| **598** | Y1049I | 1049 | Y | I | 0.68 | 0.1 |
| **599** | G1183P | 1183 | G | P | 0.68 | 1.18 |
| **600** | I798C | 798 | I | C | 0.68 | 0.36 |
| **601** | L738R | 738 | L | R | 0.68 | 0.39 |
| **602** | K478I | 478 | K | I | 0.68 | 0.09 |
| **603** | F682A | 682 | F | A | 0.68 | 0.62 |
| **604** | S1024P | 1024 | S | P | 0.68 | 0.14 |
| **605** | D832S | 832 | D | S | 0.68 | 0.43 |
| **606** | K390S | 390 | K | S | 0.68 | 0.06 |
| **607** | F655N | 655 | F | N | 0.67 | 0.26 |
| **608** | P647Y | 647 | P | Y | 0.67 | 1.19 |
| **609** | L914T | 914 | L | T | 0.67 | 0.66 |
| **610** | S1157Q | 1157 | S | Q | 0.67 | 0.41 |
| **611** | N928V | 928 | N | V | 0.67 | 0.09 |
| **612** | F525N | 525 | F | N | 0.67 | 0.09 |
| **613** | E858L | 858 | E | L | 0.67 | 0.15 |
| **614** | N656F | 656 | N | F | 0.67 | 0.21 |
| **615** | K272W | 272 | K | W | 0.67 | 0.11 |
| **616** | K444G | 444 | K | G | 0.67 | 0.08 |
| **617** | G902M | 902 | G | M | 0.67 | 0.17 |
| **618** | I1195K | 1195 | I | K | 0.67 | 0.09 |
| **619** | S1029K | 1029 | S | K | 0.67 | 0.37 |
| **620** | D156S | 156 | D | S | 0.66 | 0.09 |
| **621** | L727S | 727 | L | S | 0.66 | 0.49 |
| **622** | Y562E | 562 | Y | E | 0.66 | 0.24 |
| **623** | F884T | 884 | F | T | 0.66 | 0.72 |
| **624** | T967C | 967 | T | C | 0.66 | 2.42 |
| **625** | V280T | 280 | V | T | 0.66 | 2.72 |
| **626** | N861F | 861 | N | F | 0.66 | 0.16 |
| **627** | S143L | 143 | S | L | 0.65 | 0.25 |
| **628** | S1020C | 1020 | S | C | 0.65 | 0.94 |
| **629** | S394W | 394 | S | W | 0.65 | 0.08 |
| **630** | D537C | 537 | D | C | 0.65 | 1.19 |
| **631** | G624C | 624 | G | C | 0.65 | 0.38 |
| **632** | F289A | 289 | F | A | 0.65 | 0.06 |
| **633** | K92R | 92 | K | R | 0.65 | 0.16 |
| **634** | V24Q | 24 | V | Q | 0.65 | 0.12 |
| **635** | I798S | 798 | I | S | 0.65 | 0.06 |
| **636** | N179Q | 179 | N | Q | 0.65 | 0.03 |
| **637** | I241D | 241 | I | D | 0.65 | 1.08 |
| **638** | E217G | 217 | E | G | 0.65 | 0.21 |
| **639** | R182C | 182 | R | C | 0.65 | 0.13 |
| **640** | N849R | 849 | N | R | 0.65 | 0.44 |
| **641** | R1138M | 1138 | R | M | 0.65 | 0.15 |
| **642** | N577W | 577 | N | W | 0.65 | 0.05 |
| **643** | K1025Y | 1025 | K | Y | 0.65 | 0.64 |
| **644** | T480V | 480 | T | V | 0.65 | 0.07 |
| **645** | I1195G | 1195 | I | G | 0.65 | 0.16 |
| **646** | M986C | 986 | M | C | 0.65 | 0.54 |
| **647** | V411Q | 411 | V | Q | 0.64 | 0.54 |
| **648** | L914G | 914 | L | G | 0.64 | 0.23 |
| **649** | K274T | 274 | K | T | 0.64 | 0.04 |
| **650** | V758W | 758 | V | W | 0.64 | 0.08 |
| **651** | F745N | 745 | F | N | 0.64 | 1.12 |
| **652** | L94H | 94 | L | H | 0.64 | 0.54 |
| **653** | S905C | 905 | S | C | 0.64 | 0.03 |
| **654** | K310D | 310 | K | D | 0.64 | 0.72 |
| **655** | S465Q | 465 | S | Q | 0.64 | 0.1 |
| **656** | F931V | 931 | F | V | 0.64 | 0.17 |
| **657** | P342W | 342 | P | W | 0.64 | 0.02 |
| **658** | P589Y | 589 | P | Y | 0.64 | 0.64 |
| **659** | K1155V | 1155 | K | V | 0.64 | 0.06 |
| **660** | P806H | 806 | P | H | 0.64 | 0.41 |
| **661** | K269S | 269 | K | S | 0.64 | 0.07 |
| **662** | I124N | 124 | I | N | 0.64 | 0.44 |
| **663** | E416P | 416 | E | P | 0.64 | 0.7 |
| **664** | P799C | 799 | P | C | 0.63 | 0.04 |
| **665** | V958M | 958 | V | M | 0.63 | 0.25 |
| **666** | N263L | 263 | N | L | 0.63 | 0.55 |
| **667** | F1045N | 1045 | F | N | 0.63 | 0.03 |
| **668** | K1015M | 1015 | K | M | 0.63 | 0.06 |
| **669** | N30Y | 30 | N | Y | 0.63 | 0.04 |
| **670** | L593T | 593 | L | T | 0.63 | 0.64 |
| **671** | E412C | 412 | E | C | 0.63 | 0.12 |
| **672** | E852W | 852 | E | W | 0.63 | 0.39 |
| **673** | P515C | 515 | P | C | 0.63 | 0.28 |
| **674** | K26C | 26 | K | C | 0.63 | 1.1 |
| **675** | A1201G | 1201 | A | G | 0.63 | 0.54 |
| **676** | S14V | 14 | S | V | 0.63 | 0.06 |
| **677** | V428Y | 428 | V | Y | 0.63 | 0.27 |
| **678** | K269A | 269 | K | A | 0.63 | 0.16 |
| **679** | E98H | 98 | E | H | 0.63 | 0.05 |
| **680** | N226T | 226 | N | T | 0.63 | 0.35 |
| **681** | I868L | 868 | I | L | 0.63 | 0.03 |
| **682** | N864L | 864 | N | L | 0.63 | 0.54 |
| **683** | E39I | 39 | E | I | 0.63 | 0.09 |
| **684** | G1159I | 1159 | G | I | 0.63 | 0.38 |
| **685** | M1134S | 1134 | M | S | 0.63 | 1.32 |
| **686** | Y616E | 616 | Y | E | 0.63 | 0.03 |
| **687** | V491N | 491 | V | N | 0.63 | 0.16 |
| **688** | E178I | 178 | E | I | 0.63 | 0.44 |
| **689** | I1034E | 1034 | I | E | 0.63 | 0.08 |
| **690** | T777L | 777 | T | L | 0.63 | 0.34 |
| **691** | L180E | 180 | L | E | 0.63 | 0.24 |
| **692** | T814L | 814 | T | L | 0.63 | 0.05 |
| **693** | V511S | 511 | V | S | 0.62 | 0.42 |
| **694** | L210K | 210 | L | K | 0.62 | 0.2 |
| **695** | Y469A | 469 | Y | A | 0.62 | 0.08 |
| **696** | F273P | 273 | F | P | 0.62 | 0.23 |
| **697** | S163D | 163 | S | D | 0.62 | 0.59 |
| **698** | G1196S | 1196 | G | S | 0.62 | 0.38 |
| **699** | N33W | 33 | N | W | 0.62 | 0.06 |
| **700** | K591H | 591 | K | H | 0.62 | 0.64 |
| **701** | K688S | 688 | K | S | 0.62 | 0.08 |
| **702** | I996M | 996 | I | M | 0.62 | 0.01 |
| **703** | V851R | 851 | V | R | 0.62 | 0.1 |
| **704** | E913Y | 913 | E | Y | 0.62 | 0.74 |
| **705** | K478N | 478 | K | N | 0.62 | 0.05 |
| **706** | D156C | 156 | D | C | 0.62 | 0.17 |
| **707** | K536R | 536 | K | R | 0.62 | 0.01 |
| **708** | K688P | 688 | K | P | 0.62 | 0.08 |
| **709** | V758Q | 758 | V | Q | 0.62 | 0.05 |
| **710** | E125Q | 125 | E | Q | 0.61 | 0.03 |
| **711** | S739I | 739 | S | I | 0.61 | 0.44 |
| **712** | R35A | 35 | R | A | 0.61 | 0.28 |
| **713** | D823M | 823 | D | M | 0.61 | 0.45 |
| **714** | T1019D | 1019 | T | D | 0.61 | 0.03 |
| **715** | P1153L | 1153 | P | L | 0.61 | 0.05 |
| **716** | K1025A | 1025 | K | A | 0.61 | 0.15 |
| **717** | E795L | 795 | E | L | 0.61 | 0.05 |
| **718** | F1043C | 1043 | F | C | 0.61 | 0.01 |
| **719** | V491H | 491 | V | H | 0.61 | 0.17 |
| **720** | E165N | 165 | E | N | 0.61 | 0.28 |
| **721** | P799K | 799 | P | K | 0.61 | 0.09 |
| **722** | D46R | 46 | D | R | 0.61 | 0.01 |
| **723** | A685Q | 685 | A | Q | 0.61 | 0.03 |
| **724** | Y781E | 781 | Y | E | 0.6 | 0.26 |
| **725** | N30F | 30 | N | F | 0.6 | 0.02 |
| **726** | N1070R | 1070 | N | R | 0.6 | 0.01 |
| **727** | P806E | 806 | P | E | 0.6 | 0.5 |
| **728** | E913N | 913 | E | N | 0.6 | 0.05 |
| **729** | Y1018Q | 1018 | Y | Q | 0.6 | 0.54 |
| **730** | L751C | 751 | L | C | 0.6 | 0.57 |
| **731** | G588Y | 588 | G | Y | 0.6 | 0.36 |
| **732** | L927D | 927 | L | D | 0.6 | 0.16 |
| **733** | K984A | 984 | K | A | 0.6 | 0.22 |
| **734** | I1141R | 1141 | I | R | 0.6 | 0.11 |
| **735** | R86F | 86 | R | F | 0.6 | 0.11 |
| **736** | G49C | 49 | G | C | 0.6 | 0.19 |
| **737** | E207P | 207 | E | P | 0.59 | 0.34 |
| **738** | K1121P | 1121 | K | P | 0.59 | 0.15 |
| **739** | A1212G | 1212 | A | G | 0.59 | 0.09 |
| **740** | E479C | 479 | E | C | 0.59 | 0 |
| **741** | G49T | 49 | G | T | 0.59 | 0.45 |
| **742** | Y381M | 381 | Y | M | 0.59 | 0.07 |
| **743** | D156I | 156 | D | I | 0.59 | 0.11 |
| **744** | K421S | 421 | K | S | 0.59 | 0.18 |
| **745** | L308C | 308 | L | C | 0.59 | 0.03 |
| **746** | K1177Q | 1177 | K | Q | 0.59 | 0.1 |
| **747** | Y854R | 854 | Y | R | 0.59 | 0.12 |
| **748** | S394F | 394 | S | F | 0.59 | 0.93 |
| **749** | K272G | 272 | K | G | 0.59 | 0.08 |
| **750** | V357E | 357 | V | E | 0.59 | 0.15 |
| **751** | E39Q | 39 | E | Q | 0.59 | 0.15 |
| **752** | D654K | 654 | D | K | 0.59 | 0.33 |
| **753** | K1121V | 1121 | K | V | 0.59 | 0.02 |
| **754** | H759F | 759 | H | F | 0.59 | 0.29 |
| **755** | Y516G | 516 | Y | G | 0.59 | 0.41 |
| **756** | S117T | 117 | S | T | 0.59 | 0.05 |
| **757** | L756K | 756 | L | K | 0.59 | 0.05 |
| **758** | A685L | 685 | A | L | 0.58 | 0.2 |
| **759** | A652V | 652 | A | V | 0.58 | 0.69 |
| **760** | K568P | 568 | K | P | 0.58 | 0.11 |
| **761** | F474T | 474 | F | T | 0.58 | 0.07 |
| **762** | D729Y | 729 | D | Y | 0.58 | 0.52 |
| **763** | I860G | 860 | I | G | 0.58 | 0.16 |
| **764** | L400D | 400 | L | D | 0.58 | 0.05 |
| **765** | G1143I | 1143 | G | I | 0.58 | 0.05 |
| **766** | E898A | 898 | E | A | 0.58 | 0.05 |
| **767** | D194H | 194 | D | H | 0.58 | 0.93 |
| **768** | G1143R | 1143 | G | R | 0.58 | 0.02 |
| **769** | K1205Y | 1205 | K | Y | 0.58 | 0.05 |
| **770** | T307G | 307 | T | G | 0.58 | 0.06 |
| **771** | A970K | 970 | A | K | 0.58 | 0.93 |
| **772** | V1089Q | 1089 | V | Q | 0.58 | 0.05 |
| **773** | F273A | 273 | F | A | 0.58 | 0.07 |
| **774** | V842T | 842 | V | T | 0.58 | 0.4 |
| **775** | F702K | 702 | F | K | 0.58 | 0.06 |
| **776** | A406K | 406 | A | K | 0.58 | 0.25 |
| **777** | S212K | 212 | S | K | 0.58 | 0.05 |
| **778** | S143A | 143 | S | A | 0.58 | 0 |
| **779** | K514H | 514 | K | H | 0.58 | 0.07 |
| **780** | H759W | 759 | H | W | 0.58 | 0.26 |
| **781** | A1046R | 1046 | A | R | 0.58 | 0.06 |
| **782** | S431W | 431 | S | W | 0.58 | 0.05 |
| **783** | R174F | 174 | R | F | 0.58 | 0.93 |
| **784** | K116E | 116 | K | E | 0.58 | 0.05 |
| **785** | E223R | 223 | E | R | 0.58 | 0.93 |
| **786** | F1043G | 1043 | F | G | 0.58 | 0.06 |
| **787** | L856Y | 856 | L | Y | 0.58 | 0.05 |
| **788** | K267S | 267 | K | S | 0.58 | 0.05 |
| **789** | K522L | 522 | K | L | 0.58 | 0.91 |
| **790** | A172H | 172 | A | H | 0.58 | 0.05 |
| **791** | N263E | 263 | N | E | 0.57 | 0.05 |
| **792** | F931R | 931 | F | R | 0.57 | 0.05 |
| **793** | K910E | 910 | K | E | 0.57 | 0.33 |
| **794** | D708L | 708 | D | L | 0.57 | 0.06 |
| **795** | E674M | 674 | E | M | 0.57 | 0.19 |
| **796** | I860H | 860 | I | H | 0.57 | 0.05 |
| **797** | K107D | 107 | K | D | 0.57 | 0.19 |
| **798** | L1091Y | 1091 | L | Y | 0.57 | 1.03 |
| **799** | E1044Y | 1044 | E | Y | 0.57 | 0.06 |
| **800** | E137L | 137 | E | L | 0.57 | 0.62 |
| **801** | F131P | 131 | F | P | 0.57 | 0.93 |
| **802** | L914H | 914 | L | H | 0.57 | 0.93 |
| **803** | L839T | 839 | L | T | 0.57 | 0.08 |
| **804** | T1019K | 1019 | T | K | 0.57 | 1.03 |
| **805** | T1019R | 1019 | T | R | 0.57 | 1.03 |
| **806** | L459D | 459 | L | D | 0.57 | 0.93 |
| **807** | F304N | 304 | F | N | 0.57 | 0.06 |
| **808** | N238L | 238 | N | L | 0.57 | 0.05 |
| **809** | Q1136M | 1136 | Q | M | 0.57 | 0.29 |
| **810** | I1021R | 1021 | I | R | 0.57 | 1.39 |
| **811** | K167T | 167 | K | T | 0.57 | 0.05 |
| **812** | T181H | 181 | T | H | 0.57 | 0.05 |
| **813** | K972R | 972 | K | R | 0.57 | 0.08 |
| **814** | N1186M | 1186 | N | M | 0.57 | 1.03 |
| **815** | K457I | 457 | K | I | 0.57 | 0.05 |
| **816** | K457M | 457 | K | M | 0.57 | 0.93 |
| **817** | S445E | 445 | S | E | 0.57 | 0.19 |
| **818** | L324I | 324 | L | I | 0.57 | 0.16 |
| **819** | D871A | 871 | D | A | 0.57 | 0.05 |
| **820** | D450C | 450 | D | C | 0.57 | 1.03 |
| **821** | Q231P | 231 | Q | P | 0.57 | 0.05 |
| **822** | I904C | 904 | I | C | 0.57 | 0.22 |
| **823** | K595Q | 595 | K | Q | 0.57 | 0.06 |
| **824** | A406C | 406 | A | C | 0.57 | 0.29 |
| **825** | W362Y | 362 | W | Y | 0.57 | 0.92 |
| **826** | H909P | 909 | H | P | 0.57 | 0.61 |
| **827** | D625M | 625 | D | M | 0.57 | 0.06 |
| **828** | P1176N | 1176 | P | N | 0.56 | 0.05 |
| **829** | A1173K | 1173 | A | K | 0.56 | 0.05 |
| **830** | L66C | 66 | L | C | 0.56 | 1.02 |
| **831** | Q529G | 529 | Q | G | 0.56 | 0.07 |
| **832** | K1121F | 1121 | K | F | 0.56 | 0.12 |
| **833** | G532P | 532 | G | P | 0.56 | 0.07 |
| **834** | R182T | 182 | R | T | 0.56 | 0.13 |
| **835** | F490Q | 490 | F | Q | 0.56 | 1.04 |
| **836** | K1121G | 1121 | K | G | 0.56 | 0.1 |
| **837** | K614P | 614 | K | P | 0.56 | 0.64 |
| **838** | E858T | 858 | E | T | 0.56 | 0.93 |
| **839** | K811F | 811 | K | F | 0.56 | 0.05 |
| **840** | D505T | 505 | D | T | 0.56 | 0.07 |
| **841** | S1132V | 1132 | S | V | 0.56 | 0.84 |
| **842** | K522I | 522 | K | I | 0.56 | 0.31 |
| **843** | N309G | 309 | N | G | 0.56 | 0.06 |
| **844** | V1083T | 1083 | V | T | 0.56 | 2.08 |
| **845** | S780Q | 780 | S | Q | 0.56 | 0.08 |
| **846** | K427D | 427 | K | D | 0.56 | 0.05 |
| **847** | F474N | 474 | F | N | 0.56 | 0.06 |
| **848** | H1228C | 1228 | H | C | 0.56 | 0.05 |
| **849** | F289M | 289 | F | M | 0.56 | 1.16 |
| **850** | Y606L | 606 | Y | L | 0.55 | 0.06 |
| **851** | I1111A | 1111 | I | A | 0.55 | 0.26 |
| **852** | K274R | 274 | K | R | 0.55 | 0.18 |
| **853** | T230H | 230 | T | H | 0.55 | 0.06 |
| **854** | E579F | 579 | E | F | 0.55 | 0.06 |
| **855** | T16G | 16 | T | G | 0.55 | 0.95 |
| **856** | K269G | 269 | K | G | 0.55 | 0.07 |
| **857** | G353K | 353 | G | K | 0.55 | 0.3 |
| **858** | I417H | 417 | I | H | 0.55 | 0.47 |
| **859** | Y781S | 781 | Y | S | 0.55 | 0.13 |
| **860** | I860N | 860 | I | N | 0.55 | 0.04 |
| **861** | R737D | 737 | R | D | 0.55 | 0.19 |
| **862** | K1015W | 1015 | K | W | 0.55 | 0.07 |
| **863** | K361Y | 361 | K | Y | 0.55 | 0.06 |
| **864** | N582R | 582 | N | R | 0.55 | 0.06 |
| **865** | F884R | 884 | F | R | 0.55 | 0.39 |
| **866** | L210T | 210 | L | T | 0.55 | 0.31 |
| **867** | K110F | 110 | K | F | 0.55 | 0.11 |
| **868** | K1121S | 1121 | K | S | 0.55 | 0.11 |
| **869** | K917V | 917 | K | V | 0.55 | 0.31 |
| **870** | A685Y | 685 | A | Y | 0.55 | 0.23 |
| **871** | A364C | 364 | A | C | 0.55 | 0.03 |
| **872** | M949Q | 949 | M | Q | 0.54 | 0.24 |
| **873** | D1207C | 1207 | D | C | 0.54 | 0.13 |
| **874** | I1111C | 1111 | I | C | 0.54 | 0.31 |
| **875** | K444Y | 444 | K | Y | 0.54 | 0.41 |
| **876** | M1128E | 1128 | M | E | 0.54 | 0.05 |
| **877** | L301Y | 301 | L | Y | 0.54 | 0.22 |
| **878** | N1081P | 1081 | N | P | 0.54 | 0.31 |
| **879** | Y549W | 549 | Y | W | 0.54 | 0.23 |
| **880** | V280M | 280 | V | M | 0.54 | 0.26 |
| **881** | G222L | 222 | G | L | 0.54 | 0.31 |
| **882** | S1119M | 1119 | S | M | 0.54 | 0.01 |
| **883** | A166T | 166 | A | T | 0.54 | 0.31 |
| **884** | A166K | 166 | A | K | 0.54 | 0.23 |
| **885** | T1019I | 1019 | T | I | 0.54 | 0.42 |
| **886** | K1121W | 1121 | K | W | 0.54 | 0.04 |
| **887** | R747W | 747 | R | W | 0.54 | 0.54 |
| **888** | K787F | 787 | K | F | 0.54 | 0.83 |
| **889** | D122A | 122 | D | A | 0.54 | 0.03 |
| **890** | K361L | 361 | K | L | 0.54 | 0.68 |
| **891** | T181E | 181 | T | E | 0.54 | 0.05 |
| **892** | K269H | 269 | K | H | 0.53 | 0.12 |
| **893** | A966E | 966 | A | E | 0.53 | 0.12 |
| **894** | T27P | 27 | T | P | 0.53 | 0.04 |
| **895** | D1207G | 1207 | D | G | 0.53 | 0.07 |
| **896** | H502V | 502 | H | V | 0.53 | 0.48 |
| **897** | N306Q | 306 | N | Q | 0.53 | 0.04 |
| **898** | V757R | 757 | V | R | 0.53 | 0.15 |
| **899** | R482Y | 482 | R | Y | 0.53 | 0.05 |
| **900** | Y918M | 918 | Y | M | 0.53 | 0.16 |
| **901** | L1098M | 1098 | L | M | 0.53 | 0.01 |
| **902** | Y605M | 605 | Y | M | 0.53 | 0.05 |
| **903** | L914E | 914 | L | E | 0.53 | 0.03 |
| **904** | G475D | 475 | G | D | 0.53 | 0.08 |
| **905** | R747D | 747 | R | D | 0.53 | 0.04 |
| **906** | K591L | 591 | K | L | 0.53 | 0.36 |
| **907** | M722S | 722 | M | S | 0.53 | 0.18 |
| **908** | D133C | 133 | D | C | 0.53 | 0.12 |
| **909** | V758T | 758 | V | T | 0.53 | 0.19 |
| **910** | E285C | 285 | E | C | 0.53 | 0.41 |
| **911** | N933R | 933 | N | R | 0.52 | 0.39 |
| **912** | S763V | 763 | S | V | 0.52 | 0.11 |
| **913** | D1207N | 1207 | D | N | 0.52 | 0.15 |
| **914** | N590H | 590 | N | H | 0.52 | 0.24 |
| **915** | S394E | 394 | S | E | 0.52 | 0.03 |
| **916** | K272C | 272 | K | C | 0.52 | 0.03 |
| **917** | E683L | 683 | E | L | 0.52 | 0.11 |
| **918** | G341L | 341 | G | L | 0.52 | 0.28 |
| **919** | P271G | 271 | P | G | 0.52 | 0.11 |
| **920** | L1192C | 1192 | L | C | 0.52 | 0.01 |
| **921** | S1093I | 1093 | S | I | 0.52 | 0.18 |
| **922** | M722C | 722 | M | C | 0.52 | 0.32 |
| **923** | T1019N | 1019 | T | N | 0.52 | 0.13 |
| **924** | E885T | 885 | E | T | 0.52 | 0.27 |
| **925** | K415P | 415 | K | P | 0.52 | 0.32 |
| **926** | G475M | 475 | G | M | 0.52 | 0.06 |
| **927** | Y583T | 583 | Y | T | 0.52 | 0.43 |
| **928** | D729G | 729 | D | G | 0.52 | 0.54 |
| **929** | E1044A | 1044 | E | A | 0.52 | 0.09 |
| **930** | E221N | 221 | E | N | 0.52 | 0.44 |
| **931** | I850H | 850 | I | H | 0.51 | 0.1 |
| **932** | K568S | 568 | K | S | 0.51 | 0.23 |
| **933** | K269T | 269 | K | T | 0.51 | 0.17 |
| **934** | E1044T | 1044 | E | T | 0.51 | 0.38 |
| **935** | K269C | 269 | K | C | 0.51 | 0.03 |
| **936** | K514S | 514 | K | S | 0.51 | 0.03 |
| **937** | D1207A | 1207 | D | A | 0.51 | 0.02 |
| **938** | D559C | 559 | D | C | 0.51 | 0.43 |
| **939** | S14Q | 14 | S | Q | 0.51 | 0.05 |
| **940** | D792R | 792 | D | R | 0.51 | 0.04 |
| **941** | K387V | 387 | K | V | 0.51 | 0.07 |
| **942** | Y854G | 854 | Y | G | 0.51 | 0.12 |
| **943** | K499A | 499 | K | A | 0.51 | 0.14 |
| **944** | R482W | 482 | R | W | 0.51 | 0.15 |
| **945** | D919I | 919 | D | I | 0.51 | 0.83 |
| **946** | R1165Q | 1165 | R | Q | 0.5 | 0.27 |
| **947** | A1194W | 1194 | A | W | 0.5 | 0.93 |
| **948** | Y1066C | 1066 | Y | C | 0.5 | 0.15 |
| **949** | S763K | 763 | S | K | 0.5 | 0.02 |
| **950** | K1121T | 1121 | K | T | 0.5 | 0.14 |
| **951** | I859P | 859 | I | P | 0.5 | 0.94 |
| **952** | G576N | 576 | G | N | 0.5 | 0.43 |
| **953** | K72G | 72 | K | G | 0.5 | 0.14 |
| **954** | V228C | 228 | V | C | 0.5 | 0.22 |
| **955** | T1016F | 1016 | T | F | 0.5 | 0.15 |
| **956** | Y781R | 781 | Y | R | 0.5 | 0.1 |
| **957** | E285P | 285 | E | P | 0.5 | 0.03 |
| **958** | S1024F | 1024 | S | F | 0.5 | 0.12 |
| **959** | S1053V | 1053 | S | V | 0.5 | 0.15 |
| **960** | I22R | 22 | I | R | 0.5 | 0.53 |
| **961** | I184E | 184 | I | E | 0.5 | 0.36 |
| **962** | N179L | 179 | N | L | 0.5 | 0.07 |
| **963** | S1024V | 1024 | S | V | 0.5 | 0.05 |
| **964** | E416Q | 416 | E | Q | 0.5 | 0.11 |
| **965** | R182M | 182 | R | M | 0.5 | 0.27 |
| **966** | R645T | 645 | R | T | 0.49 | 0.61 |
| **967** | R182A | 182 | R | A | 0.49 | 0.07 |
| **968** | E674L | 674 | E | L | 0.49 | 0.02 |
| **969** | D283I | 283 | D | I | 0.49 | 0.06 |
| **970** | S394T | 394 | S | T | 0.49 | 0 |
| **971** | N889T | 889 | N | T | 0.49 | 0.08 |
| **972** | K1155S | 1155 | K | S | 0.49 | 0.42 |
| **973** | S248E | 248 | S | E | 0.49 | 0.05 |
| **974** | A970G | 970 | A | G | 0.49 | 0.16 |
| **975** | K1015G | 1015 | K | G | 0.49 | 0.04 |
| **976** | F197R | 197 | F | R | 0.49 | 0.07 |
| **977** | K116Y | 116 | K | Y | 0.49 | 0.06 |
| **978** | E88P | 88 | E | P | 0.49 | 0.33 |
| **979** | E913R | 913 | E | R | 0.49 | 0.03 |
| **980** | V783I | 783 | V | I | 0.49 | 0.5 |
| **981** | A685F | 685 | A | F | 0.49 | 0.26 |
| **982** | V428Q | 428 | V | Q | 0.49 | 0.05 |
| **983** | D213K | 213 | D | K | 0.49 | 0.18 |
| **984** | E88R | 88 | E | R | 0.49 | 0.21 |
| **985** | G353V | 353 | G | V | 0.49 | 0.13 |
| **986** | D871C | 871 | D | C | 0.49 | 0.57 |
| **987** | E835Q | 835 | E | Q | 0.48 | 0.19 |
| **988** | L629N | 629 | L | N | 0.48 | 0.32 |
| **989** | K84A | 84 | K | A | 0.48 | 0.88 |
| **990** | T266Q | 266 | T | Q | 0.48 | 0.68 |
| **991** | F1045T | 1045 | F | T | 0.48 | 0.18 |
| **992** | K272S | 272 | K | S | 0.48 | 0.11 |
| **993** | N656W | 656 | N | W | 0.48 | 0.16 |
| **994** | I922L | 922 | I | L | 0.48 | 0.07 |
| **995** | K568A | 568 | K | A | 0.48 | 0.16 |
| **996** | K167M | 167 | K | M | 0.48 | 0.41 |
| **997** | A1022D | 1022 | A | D | 0.48 | 0.22 |
| **998** | F709N | 709 | F | N | 0.48 | 0.5 |
| **999** | Y854E | 854 | Y | E | 0.48 | 0.11 |
| **1000** | R737M | 737 | R | M | 0.48 | 0.03 |
| **1001** | F131N | 131 | F | N | 0.48 | 1.69 |
| **1002** | I417L | 417 | I | L | 0.48 | 0.27 |
| **1003** | K478H | 478 | K | H | 0.48 | 0.05 |
| **1004** | V690R | 690 | V | R | 0.48 | 0.12 |
| **1005** | K1025V | 1025 | K | V | 0.48 | 0.02 |
| **1006** | E981R | 981 | E | R | 0.47 | 0.5 |
| **1007** | S681F | 681 | S | F | 0.47 | 0.1 |
| **1008** | K807L | 807 | K | L | 0.47 | 0.13 |
| **1009** | K110V | 110 | K | V | 0.47 | 0.11 |
| **1010** | E683V | 683 | E | V | 0.47 | 0.01 |
| **1011** | T1142W | 1142 | T | W | 0.47 | 0.13 |
| **1012** | S143C | 143 | S | C | 0.47 | 0.09 |
| **1013** | K1050R | 1050 | K | R | 0.47 | 0.02 |
| **1014** | D156R | 156 | D | R | 0.47 | 0.04 |
| **1015** | K1015L | 1015 | K | L | 0.47 | 0.08 |
| **1016** | E835R | 835 | E | R | 0.47 | 0.19 |
| **1017** | N577G | 577 | N | G | 0.47 | 0.08 |
| **1018** | T776I | 776 | T | I | 0.47 | 1.44 |
| **1019** | T776R | 776 | T | R | 0.47 | 1.43 |
| **1020** | I850M | 850 | I | M | 0.47 | 0.16 |
| **1021** | R836Q | 836 | R | Q | 0.47 | 0.19 |
| **1022** | S394M | 394 | S | M | 0.47 | 0.14 |
| **1023** | E217W | 217 | E | W | 0.47 | 0.11 |
| **1024** | V441P | 441 | V | P | 0.47 | 0.05 |
| **1025** | K1121Q | 1121 | K | Q | 0.47 | 0.08 |
| **1026** | D156P | 156 | D | P | 0.47 | 0.07 |
| **1027** | G477E | 477 | G | E | 0.47 | 0.07 |
| **1028** | E398K | 398 | E | K | 0.46 | 0.45 |
| **1029** | N590M | 590 | N | M | 0.46 | 0.33 |
| **1030** | K536M | 536 | K | M | 0.46 | 1.29 |
| **1031** | P997K | 997 | P | K | 0.46 | 0.25 |
| **1032** | Y781D | 781 | Y | D | 0.46 | 0 |
| **1033** | F1127A | 1127 | F | A | 0.46 | 0.6 |
| **1034** | K1096M | 1096 | K | M | 0.46 | 0.05 |
| **1035** | S929K | 929 | S | K | 0.46 | 0.12 |
| **1036** | K662C | 662 | K | C | 0.46 | 0.03 |
| **1037** | S1225A | 1225 | S | A | 0.46 | 0.01 |
| **1038** | K937H | 937 | K | H | 0.46 | 0.31 |
| **1039** | K1061F | 1061 | K | F | 0.46 | 0.07 |
| **1040** | N630W | 630 | N | W | 0.46 | 0.11 |
| **1041** | V816K | 816 | V | K | 0.46 | 0.1 |
| **1042** | T1145H | 1145 | T | H | 0.46 | 0.82 |
| **1043** | E412Y | 412 | E | Y | 0.46 | 0.07 |
| **1044** | L585R | 585 | L | R | 0.46 | 0.21 |
| **1045** | K520T | 520 | K | T | 0.46 | 0.08 |
| **1046** | V491Q | 491 | V | Q | 0.46 | 0.14 |
| **1047** | A1129C | 1129 | A | C | 0.46 | 0.15 |
| **1048** | D1207P | 1207 | D | P | 0.46 | 0.03 |
| **1049** | K269Q | 269 | K | Q | 0.46 | 0 |
| **1050** | K984F | 984 | K | F | 0.46 | 0.28 |
| **1051** | L839V | 839 | L | V | 0.46 | 0.1 |
| **1052** | H759E | 759 | H | E | 0.46 | 0.73 |
| **1053** | E885Y | 885 | E | Y | 0.46 | 0.29 |
| **1054** | K1061A | 1061 | K | A | 0.46 | 0.09 |
| **1055** | G973K | 973 | G | K | 0.45 | 0.24 |
| **1056** | S1140F | 1140 | S | F | 0.45 | 0.08 |
| **1057** | K272A | 272 | K | A | 0.45 | 0.02 |
| **1058** | D1207R | 1207 | D | R | 0.45 | 0.11 |
| **1059** | E125L | 125 | E | L | 0.45 | 0.09 |
| **1060** | D283S | 283 | D | S | 0.45 | 0.02 |
| **1061** | N481W | 481 | N | W | 0.45 | 0.21 |
| **1062** | D1207M | 1207 | D | M | 0.45 | 0.07 |
| **1063** | P799T | 799 | P | T | 0.45 | 0.07 |
| **1064** | Y781G | 781 | Y | G | 0.45 | 0.05 |
| **1065** | S763N | 763 | S | N | 0.45 | 0.03 |
| **1066** | A1046Y | 1046 | A | Y | 0.45 | 0.34 |
| **1067** | I860C | 860 | I | C | 0.45 | 0.54 |
| **1068** | S445Q | 445 | S | Q | 0.45 | 0.05 |
| **1069** | E95L | 95 | E | L | 0.45 | 0.16 |
| **1070** | F597T | 597 | F | T | 0.45 | 0.99 |
| **1071** | F224A | 224 | F | A | 0.45 | 0.34 |
| **1072** | R867S | 867 | R | S | 0.45 | 0.6 |
| **1073** | E1039S | 1039 | E | S | 0.45 | 0.15 |
| **1074** | A749E | 749 | A | E | 0.45 | 0.73 |
| **1075** | I765R | 765 | I | R | 0.45 | 0.26 |
| **1076** | M746R | 746 | M | R | 0.45 | 0.34 |
| **1077** | K1177L | 1177 | K | L | 0.44 | 0.1 |
| **1078** | F197A | 197 | F | A | 0.44 | 0.07 |
| **1079** | I425G | 425 | I | G | 0.44 | 0.73 |
| **1080** | M1128D | 1128 | M | D | 0.44 | 0.06 |
| **1081** | V216C | 216 | V | C | 0.44 | 0.05 |
| **1082** | K614Y | 614 | K | Y | 0.44 | 0.26 |
| **1083** | H200F | 200 | H | F | 0.44 | 0.16 |
| **1084** | G991C | 991 | G | C | 0.44 | 0.72 |
| **1085** | K413Y | 413 | K | Y | 0.44 | 0.05 |
| **1086** | K269E | 269 | K | E | 0.44 | 0.04 |
| **1087** | N311G | 311 | N | G | 0.44 | 0.84 |
| **1088** | T307M | 307 | T | M | 0.44 | 0.84 |
| **1089** | I138A | 138 | I | A | 0.44 | 0.15 |
| **1090** | F728C | 728 | F | C | 0.44 | 0.72 |
| **1091** | L779A | 779 | L | A | 0.44 | 0.01 |
| **1092** | A493N | 493 | A | N | 0.44 | 0.06 |
| **1093** | N327H | 327 | N | H | 0.44 | 0.26 |
| **1094** | S644K | 644 | S | K | 0.44 | 0.12 |
| **1095** | S212T | 212 | S | T | 0.44 | 0.83 |
| **1096** | E313T | 313 | E | T | 0.44 | 1.14 |
| **1097** | K272Y | 272 | K | Y | 0.44 | 0.02 |
| **1098** | E330K | 330 | E | K | 0.44 | 0.1 |
| **1099** | I138G | 138 | I | G | 0.44 | 0.83 |
| **1100** | G1009H | 1009 | G | H | 0.44 | 0.72 |
| **1101** | T1092G | 1092 | T | G | 0.44 | 0.03 |
| **1102** | E852Q | 852 | E | Q | 0.44 | 0.42 |
| **1103** | E1097Q | 1097 | E | Q | 0.43 | 0.83 |
| **1104** | F810R | 810 | F | R | 0.43 | 0.45 |
| **1105** | N861H | 861 | N | H | 0.43 | 0.73 |
| **1106** | E913I | 913 | E | I | 0.43 | 0.08 |
| **1107** | F109K | 109 | F | K | 0.43 | 0.73 |
| **1108** | K89M | 89 | K | M | 0.43 | 0.05 |
| **1109** | K1015Q | 1015 | K | Q | 0.43 | 0.02 |
| **1110** | R102E | 102 | R | E | 0.43 | 0.83 |
| **1111** | T1142A | 1142 | T | A | 0.43 | 0.13 |
| **1112** | S710W | 710 | S | W | 0.43 | 0.72 |
| **1113** | D122R | 122 | D | R | 0.43 | 0.09 |
| **1114** | K269V | 269 | K | V | 0.43 | 0.03 |
| **1115** | D611Q | 611 | D | Q | 0.43 | 0.03 |
| **1116** | S609F | 609 | S | F | 0.43 | 0.84 |
| **1117** | T152G | 152 | T | G | 0.43 | 0.05 |
| **1118** | Y605I | 605 | Y | I | 0.43 | 0.08 |
| **1119** | I951Q | 951 | I | Q | 0.43 | 0.73 |
| **1120** | M626P | 626 | M | P | 0.43 | 0.02 |
| **1121** | N76L | 76 | N | L | 0.43 | 0.73 |
| **1122** | F1010P | 1010 | F | P | 0.43 | 0.72 |
| **1123** | D136L | 136 | D | L | 0.43 | 0.83 |
| **1124** | S394K | 394 | S | K | 0.43 | 0.08 |
| **1125** | N837V | 837 | N | V | 0.43 | 0.25 |
| **1126** | K278C | 278 | K | C | 0.43 | 0.26 |
| **1127** | N862C | 862 | N | C | 0.43 | 0.26 |
| **1128** | A172E | 172 | A | E | 0.43 | 0.83 |
| **1129** | G291K | 291 | G | K | 0.43 | 0.23 |
| **1130** | F81N | 81 | F | N | 0.43 | 0.14 |
| **1131** | I259G | 259 | I | G | 0.43 | 0.03 |
| **1132** | A901K | 901 | A | K | 0.43 | 0.83 |
| **1133** | V216A | 216 | V | A | 0.43 | 0 |
| **1134** | A1201I | 1201 | A | I | 0.43 | 0.25 |
| **1135** | F436R | 436 | F | R | 0.43 | 0.58 |
| **1136** | F109C | 109 | F | C | 0.43 | 0.17 |
| **1137** | A923E | 923 | A | E | 0.43 | 0.83 |
| **1138** | N75P | 75 | N | P | 0.43 | 0.16 |
| **1139** | I911G | 911 | I | G | 0.43 | 0.83 |
| **1140** | G477S | 477 | G | S | 0.43 | 0.08 |
| **1141** | D156Y | 156 | D | Y | 0.43 | 0.12 |
| **1142** | K269W | 269 | K | W | 0.43 | 0.16 |
| **1143** | Y956Q | 956 | Y | Q | 0.43 | 0.73 |
| **1144** | V236H | 236 | V | H | 0.43 | 0.83 |
| **1145** | L1115Y | 1115 | L | Y | 0.43 | 0.83 |
| **1146** | V816H | 816 | V | H | 0.43 | 0.83 |
| **1147** | K272F | 272 | K | F | 0.43 | 0.07 |
| **1148** | D461W | 461 | D | W | 0.43 | 0.26 |
| **1149** | N1186Y | 1186 | N | Y | 0.43 | 0.82 |
| **1150** | K265H | 265 | K | H | 0.43 | 0.83 |
| **1151** | K707H | 707 | K | H | 0.43 | 0.72 |
| **1152** | G335T | 335 | G | T | 0.43 | 0.14 |
| **1153** | L738K | 738 | L | K | 0.43 | 0.83 |
| **1154** | D122G | 122 | D | G | 0.43 | 0.26 |
| **1155** | I581V | 581 | I | V | 0.43 | 0.14 |
| **1156** | K1121H | 1121 | K | H | 0.43 | 0.16 |
| **1157** | I78S | 78 | I | S | 0.43 | 0.82 |
| **1158** | I1021S | 1021 | I | S | 0.43 | 0.26 |
| **1159** | Y705C | 705 | Y | C | 0.43 | 0.02 |
| **1160** | F473R | 473 | F | R | 0.42 | 0.14 |
| **1161** | A451E | 451 | A | E | 0.42 | 0.22 |
| **1162** | L927K | 927 | L | K | 0.42 | 0.25 |
| **1163** | V216H | 216 | V | H | 0.42 | 0.51 |
| **1164** | S163I | 163 | S | I | 0.42 | 0.72 |
| **1165** | S332L | 332 | S | L | 0.42 | 0.72 |
| **1166** | Y1102N | 1102 | Y | N | 0.42 | 0.26 |
| **1167** | M1035F | 1035 | M | F | 0.42 | 0.06 |
| **1168** | I612E | 612 | I | E | 0.42 | 0.72 |
| **1169** | A1184W | 1184 | A | W | 0.42 | 0.82 |
| **1170** | E98W | 98 | E | W | 0.42 | 0.06 |
| **1171** | D1041V | 1041 | D | V | 0.42 | 0.4 |
| **1172** | S1024W | 1024 | S | W | 0.42 | 0.24 |
| **1173** | I369H | 369 | I | H | 0.42 | 0.06 |
| **1174** | N157P | 157 | N | P | 0.42 | 0.72 |
| **1175** | K752H | 752 | K | H | 0.42 | 0.73 |
| **1176** | A1057D | 1057 | A | D | 0.42 | 0.26 |
| **1177** | I841C | 841 | I | C | 0.42 | 0.13 |
| **1178** | R359K | 359 | R | K | 0.42 | 0.72 |
| **1179** | K1017F | 1017 | K | F | 0.42 | 0.83 |
| **1180** | F530R | 530 | F | R | 0.42 | 0.85 |
| **1181** | T777P | 777 | T | P | 0.42 | 0.24 |
| **1182** | G242I | 242 | G | I | 0.42 | 0.26 |
| **1183** | N889M | 889 | N | M | 0.42 | 0.06 |
| **1184** | K1121L | 1121 | K | L | 0.42 | 0 |
| **1185** | K1015S | 1015 | K | S | 0.42 | 0.05 |
| **1186** | I736H | 736 | I | H | 0.42 | 0.83 |
| **1187** | Y700G | 700 | Y | G | 0.42 | 0.72 |
| **1188** | D665H | 665 | D | H | 0.42 | 0.04 |
| **1189** | Q203D | 203 | Q | D | 0.42 | 0.17 |
| **1190** | G477V | 477 | G | V | 0.42 | 0.08 |
| **1191** | E479W | 479 | E | W | 0.42 | 0.07 |
| **1192** | T246C | 246 | T | C | 0.42 | 0.15 |
| **1193** | I470L | 470 | I | L | 0.42 | 0.26 |
| **1194** | S282N | 282 | S | N | 0.42 | 0.72 |
| **1195** | K391Y | 391 | K | Y | 0.42 | 0.26 |
| **1196** | K897A | 897 | K | A | 0.42 | 0.02 |
| **1197** | Y1221M | 1221 | Y | M | 0.42 | 0.02 |
| **1198** | Y903P | 903 | Y | P | 0.42 | 0.83 |
| **1199** | V828Y | 828 | V | Y | 0.42 | 0.72 |
| **1200** | Y903G | 903 | Y | G | 0.42 | 0.72 |
| **1201** | Y554M | 554 | Y | M | 0.42 | 0.14 |
| **1202** | K1015C | 1015 | K | C | 0.42 | 0.13 |
| **1203** | L497D | 497 | L | D | 0.42 | 0.71 |
| **1204** | I860F | 860 | I | F | 0.42 | 0.05 |
| **1205** | I569D | 569 | I | D | 0.42 | 0.27 |
| **1206** | L101C | 101 | L | C | 0.42 | 0.18 |
| **1207** | F227H | 227 | F | H | 0.42 | 0.83 |
| **1208** | I234D | 234 | I | D | 0.42 | 0.83 |
| **1209** | F1027M | 1027 | F | M | 0.42 | 0.19 |
| **1210** | K107M | 107 | K | M | 0.41 | 0.08 |
| **1211** | Y781W | 781 | Y | W | 0.41 | 0.18 |
| **1212** | E98M | 98 | E | M | 0.41 | 0.96 |
| **1213** | D384T | 384 | D | T | 0.41 | 0.03 |
| **1214** | L693D | 693 | L | D | 0.41 | 0.48 |
| **1215** | K349G | 349 | K | G | 0.41 | 0.26 |
| **1216** | L281Y | 281 | L | Y | 0.41 | 0.36 |
| **1217** | D572W | 572 | D | W | 0.41 | 0.12 |
| **1218** | L566H | 566 | L | H | 0.41 | 0.84 |
| **1219** | E913K | 913 | E | K | 0.41 | 0.02 |
| **1220** | I557V | 557 | I | V | 0.41 | 0.27 |
| **1221** | N731E | 731 | N | E | 0.41 | 0.17 |
| **1222** | T716Q | 716 | T | Q | 0.41 | 1.78 |
| **1223** | I831L | 831 | I | L | 0.41 | 0.1 |
| **1224** | Y1018E | 1018 | Y | E | 0.41 | 0 |
| **1225** | L1065R | 1065 | L | R | 0.41 | 0.16 |
| **1226** | I419R | 419 | I | R | 0.41 | 0.09 |
| **1227** | I314Q | 314 | I | Q | 0.41 | 0.14 |
| **1228** | M1134R | 1134 | M | R | 0.41 | 0.02 |
| **1229** | A1188L | 1188 | A | L | 0.41 | 0.79 |
| **1230** | S168V | 168 | S | V | 0.41 | 0.14 |
| **1231** | P799R | 799 | P | R | 0.41 | 0.03 |
| **1232** | C805L | 805 | C | L | 0.41 | 0.17 |
| **1233** | T16A | 16 | T | A | 0.41 | 0.74 |
| **1234** | D782Q | 782 | D | Q | 0.41 | 0.08 |
| **1235** | D122M | 122 | D | M | 0.41 | 0.25 |
| **1236** | Y1162A | 1162 | Y | A | 0.41 | 0.73 |
| **1237** | S143G | 143 | S | G | 0.41 | 0.05 |
| **1238** | S1020L | 1020 | S | L | 0.41 | 0.01 |
| **1239** | K1205P | 1205 | K | P | 0.41 | 0.46 |
| **1240** | G254D | 254 | G | D | 0.4 | 0.43 |
| **1241** | R182G | 182 | R | G | 0.4 | 0 |
| **1242** | R1112W | 1112 | R | W | 0.4 | 0.31 |
| **1243** | T480I | 480 | T | I | 0.4 | 0.35 |
| **1244** | L492H | 492 | L | H | 0.4 | 0.06 |
| **1245** | K52P | 52 | K | P | 0.4 | 0.74 |
| **1246** | L744M | 744 | L | M | 0.4 | 0.17 |
| **1247** | L210N | 210 | L | N | 0.4 | 0.07 |
| **1248** | I1072S | 1072 | I | S | 0.4 | 0.21 |
| **1249** | G734Q | 734 | G | Q | 0.4 | 0.05 |
| **1250** | I205C | 205 | I | C | 0.4 | 0.07 |
| **1251** | Y115P | 115 | Y | P | 0.4 | 0.29 |
| **1252** | T1224D | 1224 | T | D | 0.4 | 0.69 |
| **1253** | L699D | 699 | L | D | 0.4 | 0.09 |
| **1254** | G1196E | 1196 | G | E | 0.4 | 0.11 |
| **1255** | K3M | 3 | K | M | 0.4 | 0.75 |
| **1256** | K272V | 272 | K | V | 0.4 | 0.09 |
| **1257** | L876A | 876 | L | A | 0.4 | 0.1 |
| **1258** | R482H | 482 | R | H | 0.4 | 0.11 |
| **1259** | Y678E | 678 | Y | E | 0.4 | 0.72 |
| **1260** | E292M | 292 | E | M | 0.4 | 0.03 |
| **1261** | Y77W | 77 | Y | W | 0.4 | 0.25 |
| **1262** | S1024G | 1024 | S | G | 0.4 | 0.04 |
| **1263** | E95A | 95 | E | A | 0.4 | 0.02 |
| **1264** | E981F | 981 | E | F | 0.4 | 0.11 |
| **1265** | A1173N | 1173 | A | N | 0.4 | 0.13 |
| **1266** | D495E | 495 | D | E | 0.4 | 0.08 |
| **1267** | K272P | 272 | K | P | 0.4 | 0.14 |
| **1268** | A404R | 404 | A | R | 0.4 | 0.21 |
| **1269** | V921G | 921 | V | G | 0.4 | 0.04 |
| **1270** | Y1221K | 1221 | Y | K | 0.4 | 0.12 |
| **1271** | K373F | 373 | K | F | 0.4 | 0.2 |
| **1272** | F728S | 728 | F | S | 0.4 | 0.45 |
| **1273** | I418K | 418 | I | K | 0.4 | 0.02 |
| **1274** | E1217F | 1217 | E | F | 0.4 | 0.83 |
| **1275** | F224G | 224 | F | G | 0.4 | 0.23 |
| **1276** | S143M | 143 | S | M | 0.4 | 0.05 |
| **1277** | D156G | 156 | D | G | 0.4 | 0 |
| **1278** | A1022E | 1022 | A | E | 0.4 | 0.17 |
| **1279** | N481F | 481 | N | F | 0.4 | 0.13 |
| **1280** | I69P | 69 | I | P | 0.4 | 0.09 |
| **1281** | K361V | 361 | K | V | 0.4 | 0.06 |
| **1282** | T776E | 776 | T | E | 0.4 | 1.72 |
| **1283** | K1064M | 1064 | K | M | 0.39 | 0.59 |
| **1284** | G475K | 475 | G | K | 0.39 | 0.11 |
| **1285** | K1026P | 1026 | K | P | 0.39 | 0.11 |
| **1286** | S409D | 409 | S | D | 0.39 | 0.21 |
| **1287** | N577A | 577 | N | A | 0.39 | 0.18 |
| **1288** | Y646S | 646 | Y | S | 0.39 | 0.21 |
| **1289** | I951L | 951 | I | L | 0.39 | 0.14 |
| **1290** | D1163C | 1163 | D | C | 0.39 | 0.11 |
| **1291** | G114Q | 114 | G | Q | 0.39 | 0.24 |
| **1292** | N862L | 862 | N | L | 0.39 | 0.25 |
| **1293** | N656I | 656 | N | I | 0.39 | 0.37 |
| **1294** | K1177E | 1177 | K | E | 0.39 | 0.22 |
| **1295** | L94D | 94 | L | D | 0.39 | 0.08 |
| **1296** | S2H | 2 | S | H | 0.39 | 0.75 |
| **1297** | K478E | 478 | K | E | 0.39 | 0.11 |
| **1298** | L324E | 324 | L | E | 0.39 | 0.06 |
| **1299** | L779V | 779 | L | V | 0.39 | 0.25 |
| **1300** | I419Q | 419 | I | Q | 0.39 | 0.02 |
| **1301** | A563G | 563 | A | G | 0.39 | 0.13 |
| **1302** | T1019W | 1019 | T | W | 0.39 | 0.03 |
| **1303** | F1052I | 1052 | F | I | 0.39 | 0.14 |
| **1304** | K932F | 932 | K | F | 0.39 | 0.05 |
| **1305** | K910I | 910 | K | I | 0.39 | 0.05 |
| **1306** | Q567P | 567 | Q | P | 0.39 | 0.04 |
| **1307** | V38D | 38 | V | D | 0.39 | 0.09 |
| **1308** | A801D | 801 | A | D | 0.39 | 0.05 |
| **1309** | G49I | 49 | G | I | 0.39 | 0.04 |
| **1310** | V411D | 411 | V | D | 0.39 | 0.02 |
| **1311** | F440E | 440 | F | E | 0.39 | 0.05 |
| **1312** | Y827R | 827 | Y | R | 0.38 | 0.14 |
| **1313** | E443Y | 443 | E | Y | 0.38 | 0.34 |
| **1314** | Y57M | 57 | Y | M | 0.38 | 0.03 |
| **1315** | Y700V | 700 | Y | V | 0.38 | 0.09 |
| **1316** | N468E | 468 | N | E | 0.38 | 0.2 |
| **1317** | M187R | 187 | M | R | 0.38 | 0.03 |
| **1318** | G968P | 968 | G | P | 0.38 | 0.34 |
| **1319** | M603S | 603 | M | S | 0.38 | 0.01 |
| **1320** | N311E | 311 | N | E | 0.38 | 0.09 |
| **1321** | N91G | 91 | N | G | 0.38 | 0.48 |
| **1322** | K274Q | 274 | K | Q | 0.38 | 0.07 |
| **1323** | T181D | 181 | T | D | 0.38 | 0.12 |
| **1324** | R305V | 305 | R | V | 0.38 | 0.05 |
| **1325** | E1204R | 1204 | E | R | 0.38 | 0.17 |
| **1326** | K448C | 448 | K | C | 0.38 | 0.01 |
| **1327** | L446W | 446 | L | W | 0.38 | 0.09 |
| **1328** | A1122K | 1122 | A | K | 0.38 | 0.16 |
| **1329** | R35N | 35 | R | N | 0.38 | 0.74 |
| **1330** | L727I | 727 | L | I | 0.38 | 0.34 |
| **1331** | I196W | 196 | I | W | 0.38 | 0.31 |
| **1332** | N732I | 732 | N | I | 0.38 | 0.75 |
| **1333** | Y58S | 58 | Y | S | 0.38 | 0.08 |
| **1334** | I78V | 78 | I | V | 0.38 | 0.32 |
| **1335** | K413V | 413 | K | V | 0.38 | 0.21 |
| **1336** | K26V | 26 | K | V | 0.38 | 0.74 |
| **1337** | K878T | 878 | K | T | 0.38 | 0.04 |
| **1338** | E913L | 913 | E | L | 0.37 | 0.06 |
| **1339** | A667R | 667 | A | R | 0.37 | 0.18 |
| **1340** | N857S | 857 | N | S | 0.37 | 0.34 |
| **1341** | S763T | 763 | S | T | 0.37 | 0.04 |
| **1342** | K1017A | 1017 | K | A | 0.37 | 0.08 |
| **1343** | F530G | 530 | F | G | 0.37 | 0.56 |
| **1344** | V491E | 491 | V | E | 0.37 | 0.05 |
| **1345** | G846W | 846 | G | W | 0.37 | 0 |
| **1346** | K1210L | 1210 | K | L | 0.37 | 0.82 |
| **1347** | D665R | 665 | D | R | 0.37 | 0.06 |
| **1348** | I1072Y | 1072 | I | Y | 0.37 | 0.33 |
| **1349** | S296V | 296 | S | V | 0.37 | 0.01 |
| **1350** | N955L | 955 | N | L | 0.37 | 0.06 |
| **1351** | I911Q | 911 | I | Q | 0.37 | 0.64 |
| **1352** | I189E | 189 | I | E | 0.37 | 0.17 |
| **1353** | D156V | 156 | D | V | 0.37 | 0.05 |
| **1354** | T1016E | 1016 | T | E | 0.37 | 0.18 |
| **1355** | Y646R | 646 | Y | R | 0.37 | 0.61 |
| **1356** | N179A | 179 | N | A | 0.37 | 0.36 |
| **1357** | F436M | 436 | F | M | 0.37 | 0.03 |
| **1358** | T814A | 814 | T | A | 0.37 | 0.16 |
| **1359** | V783R | 783 | V | R | 0.37 | 0.08 |
| **1360** | E285T | 285 | E | T | 0.37 | 0.24 |
| **1361** | E791T | 791 | E | T | 0.37 | 0.05 |
| **1362** | G848R | 848 | G | R | 0.37 | 0.08 |
| **1363** | T891Y | 891 | T | Y | 0.37 | 0.04 |
| **1364** | E479L | 479 | E | L | 0.37 | 0.11 |
| **1365** | D877T | 877 | D | T | 0.37 | 0.73 |
| **1366** | Q975Y | 975 | Q | Y | 0.36 | 0.13 |
| **1367** | T152R | 152 | T | R | 0.36 | 0.04 |
| **1368** | Y294K | 294 | Y | K | 0.36 | 0.34 |
| **1369** | E88I | 88 | E | I | 0.36 | 0 |
| **1370** | V24M | 24 | V | M | 0.36 | 0.29 |
| **1371** | P589V | 589 | P | V | 0.36 | 0.1 |
| **1372** | A685N | 685 | A | N | 0.36 | 0.35 |
| **1373** | S710K | 710 | S | K | 0.36 | 0.31 |
| **1374** | K421I | 421 | K | I | 0.36 | 0.26 |
| **1375** | N582W | 582 | N | W | 0.36 | 0.2 |
| **1376** | D1005S | 1005 | D | S | 0.36 | 1.11 |
| **1377** | M1131P | 1131 | M | P | 0.36 | 0.28 |
| **1378** | K1025F | 1025 | K | F | 0.36 | 0.05 |
| **1379** | Y872D | 872 | Y | D | 0.36 | 0.02 |
| **1380** | K940R | 940 | K | R | 0.36 | 0.48 |
| **1381** | E913G | 913 | E | G | 0.36 | 0.11 |
| **1382** | S143R | 143 | S | R | 0.36 | 0.08 |
| **1383** | M558Y | 558 | M | Y | 0.36 | 0.1 |
| **1384** | N889D | 889 | N | D | 0.36 | 0.04 |
| **1385** | D1056E | 1056 | D | E | 0.36 | 0.15 |
| **1386** | V422Y | 422 | V | Y | 0.36 | 0.28 |
| **1387** | A1046H | 1046 | A | H | 0.36 | 0.62 |
| **1388** | V441N | 441 | V | N | 0.36 | 0.02 |
| **1389** | I636L | 636 | I | L | 0.36 | 1.32 |
| **1390** | R887A | 887 | R | A | 0.36 | 0.04 |
| **1391** | E88T | 88 | E | T | 0.36 | 0.12 |
| **1392** | F1123Q | 1123 | F | Q | 0.36 | 0.38 |
| **1393** | I22A | 22 | I | A | 0.36 | 0.2 |
| **1394** | I557S | 557 | I | S | 0.36 | 0.08 |
| **1395** | G740E | 740 | G | E | 0.36 | 0.64 |
| **1396** | H733Q | 733 | H | Q | 0.36 | 0.08 |
| **1397** | G624L | 624 | G | L | 0.36 | 0.22 |
| **1398** | E104K | 104 | E | K | 0.36 | 0.12 |
| **1399** | I829S | 829 | I | S | 0.36 | 0.23 |
| **1400** | F669C | 669 | F | C | 0.36 | 0.07 |
| **1401** | W890V | 890 | W | V | 0.36 | 0.04 |
| **1402** | L13I | 13 | L | I | 0.36 | 0.12 |
| **1403** | K937F | 937 | K | F | 0.36 | 0 |
| **1404** | A761P | 761 | A | P | 0.36 | 0.13 |
| **1405** | K1015E | 1015 | K | E | 0.36 | 0.09 |
| **1406** | K499Y | 499 | K | Y | 0.36 | 0.22 |
| **1407** | Y903D | 903 | Y | D | 0.36 | 0.11 |
| **1408** | A1181G | 1181 | A | G | 0.36 | 0.01 |
| **1409** | Y606M | 606 | Y | M | 0.36 | 0.09 |
| **1410** | N145S | 145 | N | S | 0.36 | 0.02 |
| **1411** | V38Q | 38 | V | Q | 0.36 | 0 |
| **1412** | Y705V | 705 | Y | V | 0.36 | 0.01 |
| **1413** | S987M | 987 | S | M | 0.35 | 0.31 |
| **1414** | A1046V | 1046 | A | V | 0.35 | 0.26 |
| **1415** | E913M | 913 | E | M | 0.35 | 0.02 |
| **1416** | D156N | 156 | D | N | 0.35 | 0.09 |
| **1417** | K1015P | 1015 | K | P | 0.35 | 0.05 |
| **1418** | S985Q | 985 | S | Q | 0.35 | 0.08 |
| **1419** | F884V | 884 | F | V | 0.35 | 0.16 |
| **1420** | E292V | 292 | E | V | 0.35 | 0.05 |
| **1421** | N1070L | 1070 | N | L | 0.35 | 0.76 |
| **1422** | S658F | 658 | S | F | 0.35 | 0.34 |
| **1423** | A920M | 920 | A | M | 0.35 | 0.1 |
| **1424** | S317F | 317 | S | F | 0.35 | 0.11 |
| **1425** | Y974V | 974 | Y | V | 0.35 | 0.1 |
| **1426** | Y487W | 487 | Y | W | 0.35 | 0.02 |
| **1427** | Y918L | 918 | Y | L | 0.35 | 0.31 |
| **1428** | I418N | 418 | I | N | 0.35 | 0.7 |
| **1429** | K72V | 72 | K | V | 0.35 | 0.1 |
| **1430** | D350M | 350 | D | M | 0.35 | 0.06 |
| **1431** | D188C | 188 | D | C | 0.35 | 0.01 |
| **1432** | V236C | 236 | V | C | 0.35 | 0.05 |
| **1433** | N509L | 509 | N | L | 0.35 | 0.02 |
| **1434** | Q613T | 613 | Q | T | 0.35 | 0.13 |
| **1435** | Q279D | 279 | Q | D | 0.35 | 0.44 |
| **1436** | S1140W | 1140 | S | W | 0.35 | 0.13 |
| **1437** | K272M | 272 | K | M | 0.35 | 0.06 |
| **1438** | G848T | 848 | G | T | 0.35 | 0.49 |
| **1439** | E90K | 90 | E | K | 0.35 | 0.08 |
| **1440** | N1082T | 1082 | N | T | 0.35 | 0.09 |
| **1441** | E88W | 88 | E | W | 0.35 | 0 |
| **1442** | N481V | 481 | N | V | 0.35 | 0.02 |
| **1443** | D329E | 329 | D | E | 0.35 | 0.06 |
| **1444** | I318Q | 318 | I | Q | 0.35 | 0.13 |
| **1445** | L94K | 94 | L | K | 0.35 | 0.38 |
| **1446** | Y469K | 469 | Y | K | 0.35 | 0.06 |
| **1447** | N706Y | 706 | N | Y | 0.35 | 0.01 |
| **1448** | F992G | 992 | F | G | 0.35 | 0.13 |
| **1449** | M592R | 592 | M | R | 0.35 | 0.03 |
| **1450** | N145R | 145 | N | R | 0.35 | 0.01 |
| **1451** | A685R | 685 | A | R | 0.35 | 0.45 |
| **1452** | V1089H | 1089 | V | H | 0.35 | 0.06 |
| **1453** | D823A | 823 | D | A | 0.35 | 0.05 |
| **1454** | Y827P | 827 | Y | P | 0.35 | 0.64 |
| **1455** | N718R | 718 | N | R | 0.35 | 0.2 |
| **1456** | V783Q | 783 | V | Q | 0.35 | 0.04 |
| **1457** | K698D | 698 | K | D | 0.35 | 0.02 |
| **1458** | Y1018D | 1018 | Y | D | 0.34 | 0.05 |
| **1459** | K415E | 415 | K | E | 0.34 | 0.7 |
| **1460** | K1015V | 1015 | K | V | 0.34 | 0.06 |
| **1461** | V303Y | 303 | V | Y | 0.34 | 0.59 |
| **1462** | D405F | 405 | D | F | 0.34 | 0.38 |
| **1463** | K267V | 267 | K | V | 0.34 | 0.12 |
| **1464** | Y469C | 469 | Y | C | 0.34 | 0.29 |
| **1465** | N732M | 732 | N | M | 0.34 | 0.39 |
| **1466** | Y670L | 670 | Y | L | 0.34 | 0.13 |
| **1467** | H909C | 909 | H | C | 0.34 | 0.13 |
| **1468** | W1086I | 1086 | W | I | 0.34 | 0.05 |
| **1469** | K107A | 107 | K | A | 0.34 | 0.06 |
| **1470** | F197N | 197 | F | N | 0.34 | 0.03 |
| **1471** | E178R | 178 | E | R | 0.34 | 0.05 |
| **1472** | P964N | 964 | P | N | 0.34 | 0.27 |
| **1473** | W355E | 355 | W | E | 0.34 | 0.04 |
| **1474** | F219H | 219 | F | H | 0.34 | 0.13 |
| **1475** | K274S | 274 | K | S | 0.34 | 0.05 |
| **1476** | R1033D | 1033 | R | D | 0.34 | 0.12 |
| **1477** | Q613M | 613 | Q | M | 0.34 | 0.7 |
| **1478** | E201Y | 201 | E | Y | 0.34 | 0.02 |
| **1479** | R482V | 482 | R | V | 0.34 | 0.1 |
| **1480** | D729W | 729 | D | W | 0.34 | 0.59 |
| **1481** | E285L | 285 | E | L | 0.34 | 0.47 |
| **1482** | F810C | 810 | F | C | 0.34 | 0.13 |
| **1483** | S14I | 14 | S | I | 0.34 | 0 |
| **1484** | K807N | 807 | K | N | 0.34 | 0.29 |
| **1485** | S333M | 333 | S | M | 0.34 | 0.19 |
| **1486** | K272L | 272 | K | L | 0.34 | 0.05 |
| **1487** | Y974D | 974 | Y | D | 0.34 | 0.14 |
| **1488** | H909Q | 909 | H | Q | 0.34 | 0.05 |
| **1489** | S485P | 485 | S | P | 0.34 | 0.01 |
| **1490** | E913P | 913 | E | P | 0.34 | 0.7 |
| **1491** | D1207I | 1207 | D | I | 0.34 | 0.21 |
| **1492** | M1035N | 1035 | M | N | 0.34 | 0.38 |
| **1493** | Q613C | 613 | Q | C | 0.34 | 0.01 |
| **1494** | F490A | 490 | F | A | 0.34 | 0.02 |
| **1495** | G114M | 114 | G | M | 0.34 | 0.09 |
| **1496** | I841P | 841 | I | P | 0.34 | 0.01 |
| **1497** | E39Y | 39 | E | Y | 0.34 | 0.14 |
| **1498** | F655M | 655 | F | M | 0.34 | 0.27 |
| **1499** | N263S | 263 | N | S | 0.34 | 0.15 |
| **1500** | K945I | 945 | K | I | 0.34 | 0.39 |
| **1501** | E247V | 247 | E | V | 0.34 | 0.15 |
| **1502** | R82Q | 82 | R | Q | 0.34 | 0.04 |
| **1503** | K253A | 253 | K | A | 0.34 | 0.59 |
| **1504** | Y290A | 290 | Y | A | 0.34 | 0.05 |
| **1505** | L1115N | 1115 | L | N | 0.34 | 0.59 |
| **1506** | D501K | 501 | D | K | 0.34 | 0.58 |
| **1507** | S79P | 79 | S | P | 0.34 | 0.21 |
| **1508** | E815P | 815 | E | P | 0.34 | 0.26 |
| **1509** | R482N | 482 | R | N | 0.34 | 0.59 |
| **1510** | F162C | 162 | F | C | 0.33 | 0.6 |
| **1511** | L1047N | 1047 | L | N | 0.33 | 0.02 |
| **1512** | F597I | 597 | F | I | 0.33 | 0.07 |
| **1513** | I841S | 841 | I | S | 0.33 | 0.14 |
| **1514** | N732R | 732 | N | R | 0.33 | 0.18 |
| **1515** | K1015T | 1015 | K | T | 0.33 | 0.05 |
| **1516** | K413D | 413 | K | D | 0.33 | 0.15 |
| **1517** | V376Y | 376 | V | Y | 0.33 | 0.17 |
| **1518** | N656E | 656 | N | E | 0.33 | 0.25 |
| **1519** | V303S | 303 | V | S | 0.33 | 0.22 |
| **1520** | S982V | 982 | S | V | 0.33 | 0.41 |
| **1521** | K1017L | 1017 | K | L | 0.33 | 0 |
| **1522** | K83R | 83 | K | R | 0.33 | 0.02 |
| **1523** | K1101Y | 1101 | K | Y | 0.33 | 0.5 |
| **1524** | I209T | 209 | I | T | 0.33 | 0.57 |
| **1525** | G973M | 973 | G | M | 0.33 | 0.25 |
| **1526** | E247L | 247 | E | L | 0.33 | 0.38 |
| **1527** | T776F | 776 | T | F | 0.33 | 0.13 |
| **1528** | N861S | 861 | N | S | 0.33 | 0.47 |
| **1529** | L492N | 492 | L | N | 0.33 | 0.11 |
| **1530** | K448A | 448 | K | A | 0.33 | 0.18 |
| **1531** | E90W | 90 | E | W | 0.33 | 0.18 |
| **1532** | E98T | 98 | E | T | 0.33 | 0.18 |
| **1533** | T988K | 988 | T | K | 0.33 | 0.06 |
| **1534** | K499R | 499 | K | R | 0.33 | 0.11 |
| **1535** | E743K | 743 | E | K | 0.33 | 0.23 |
| **1536** | I503S | 503 | I | S | 0.33 | 0.02 |
| **1537** | H714R | 714 | H | R | 0.33 | 0.02 |
| **1538** | Y1018S | 1018 | Y | S | 0.33 | 0.19 |
| **1539** | L261A | 261 | L | A | 0.33 | 0.7 |
| **1540** | T776W | 776 | T | W | 0.33 | 0.26 |
| **1541** | Y1068P | 1068 | Y | P | 0.33 | 0.59 |
| **1542** | P342H | 342 | P | H | 0.33 | 0.18 |
| **1543** | S780W | 780 | S | W | 0.33 | 0.13 |
| **1544** | F1099K | 1099 | F | K | 0.33 | 0.6 |
| **1545** | V758Y | 758 | V | Y | 0.33 | 0.11 |
| **1546** | V280C | 280 | V | C | 0.33 | 0.1 |
| **1547** | F1198R | 1198 | F | R | 0.33 | 0.6 |
| **1548** | D489E | 489 | D | E | 0.33 | 0.21 |
| **1549** | A334F | 334 | A | F | 0.33 | 0.41 |
| **1550** | A970C | 970 | A | C | 0.33 | 0.02 |
| **1551** | D1180T | 1180 | D | T | 0.33 | 0.69 |
| **1552** | K1017E | 1017 | K | E | 0.33 | 0.28 |
| **1553** | N718H | 718 | N | H | 0.33 | 0.15 |
| **1554** | L914Y | 914 | L | Y | 0.32 | 0.08 |
| **1555** | I557Y | 557 | I | Y | 0.32 | 0.5 |
| **1556** | M592T | 592 | M | T | 0.32 | 0.29 |
| **1557** | K274A | 274 | K | A | 0.32 | 0.01 |
| **1558** | L954R | 954 | L | R | 0.32 | 0.06 |
| **1559** | M603W | 603 | M | W | 0.32 | 0.1 |
| **1560** | D631N | 631 | D | N | 0.32 | 0.14 |
| **1561** | Y678A | 678 | Y | A | 0.32 | 0.73 |
| **1562** | K1121N | 1121 | K | N | 0.32 | 0.15 |
| **1563** | E88V | 88 | E | V | 0.32 | 0.19 |
| **1564** | K648P | 648 | K | P | 0.32 | 0.7 |
| **1565** | T776Y | 776 | T | Y | 0.32 | 0.03 |
| **1566** | K1026G | 1026 | K | G | 0.32 | 0.09 |
| **1567** | R284V | 284 | R | V | 0.32 | 1.2 |
| **1568** | Y827D | 827 | Y | D | 0.32 | 0.07 |
| **1569** | D877G | 877 | D | G | 0.32 | 0.12 |
| **1570** | E674C | 674 | E | C | 0.32 | 0.15 |
| **1571** | S431L | 431 | S | L | 0.32 | 0.88 |
| **1572** | S394H | 394 | S | H | 0.32 | 0.01 |
| **1573** | Y781Q | 781 | Y | Q | 0.32 | 0.01 |
| **1574** | K1121Y | 1121 | K | Y | 0.32 | 0.15 |
| **1575** | I205V | 205 | I | V | 0.32 | 0.26 |
| **1576** | R86A | 86 | R | A | 0.32 | 0.06 |
| **1577** | L914A | 914 | L | A | 0.32 | 0.05 |
| **1578** | L779S | 779 | L | S | 0.32 | 0.09 |
| **1579** | Y872Q | 872 | Y | Q | 0.32 | 0.03 |
| **1580** | T480E | 480 | T | E | 0.32 | 0.43 |
| **1581** | K135V | 135 | K | V | 0.32 | 0.02 |
| **1582** | A1113M | 1113 | A | M | 0.32 | 0.09 |
| **1583** | R482A | 482 | R | A | 0.32 | 0.04 |
| **1584** | D450A | 450 | D | A | 0.32 | 0.1 |
| **1585** | Q703P | 703 | Q | P | 0.32 | 0.06 |
| **1586** | A1181Q | 1181 | A | Q | 0.32 | 0.7 |
| **1587** | V907A | 907 | V | A | 0.32 | 0.03 |
| **1588** | D535E | 535 | D | E | 0.32 | 0.35 |
| **1589** | E858M | 858 | E | M | 0.32 | 0.23 |
| **1590** | S929Q | 929 | S | Q | 0.32 | 0.01 |
| **1591** | G697R | 697 | G | R | 0.32 | 0.14 |
| **1592** | N449C | 449 | N | C | 0.32 | 0.57 |
| **1593** | M558H | 558 | M | H | 0.32 | 0.04 |
| **1594** | Y294S | 294 | Y | S | 0.32 | 0.06 |
| **1595** | E539L | 539 | E | L | 0.32 | 0.06 |
| **1596** | Y294D | 294 | Y | D | 0.31 | 0.09 |
| **1597** | Q941T | 941 | Q | T | 0.31 | 0.23 |
| **1598** | F151L | 151 | F | L | 0.31 | 0.03 |
| **1599** | N186Y | 186 | N | Y | 0.31 | 0.32 |
| **1600** | P587S | 587 | P | S | 0.31 | 0.23 |
| **1601** | D46F | 46 | D | F | 0.31 | 0.15 |
| **1602** | E221W | 221 | E | W | 0.31 | 0.01 |
| **1603** | I841Y | 841 | I | Y | 0.31 | 0.02 |
| **1604** | S286K | 286 | S | K | 0.31 | 0.13 |
| **1605** | D1207Y | 1207 | D | Y | 0.31 | 0.03 |
| **1606** | K278Y | 278 | K | Y | 0.31 | 0.08 |
| **1607** | L321K | 321 | L | K | 0.31 | 0.1 |
| **1608** | F474V | 474 | F | V | 0.31 | 0.02 |
| **1609** | N1077H | 1077 | N | H | 0.31 | 0.13 |
| **1610** | E178L | 178 | E | L | 0.31 | 0.2 |
| **1611** | L1065E | 1065 | L | E | 0.31 | 0.08 |
| **1612** | Y77S | 77 | Y | S | 0.31 | 0.16 |
| **1613** | D1207T | 1207 | D | T | 0.31 | 0.07 |
| **1614** | N1166K | 1166 | N | K | 0.31 | 0.03 |
| **1615** | Y670D | 670 | Y | D | 0.31 | 0.03 |
| **1616** | N861Y | 861 | N | Y | 0.31 | 0.21 |
| **1617** | E412P | 412 | E | P | 0.31 | 1.32 |
| **1618** | E95W | 95 | E | W | 0.31 | 0.41 |
| **1619** | D708A | 708 | D | A | 0.31 | 0.19 |
| **1620** | S60T | 60 | S | T | 0.31 | 0.29 |
| **1621** | K787Y | 787 | K | Y | 0.31 | 0.21 |
| **1622** | Y700D | 700 | Y | D | 0.31 | 0.06 |
| **1623** | A1201R | 1201 | A | R | 0.31 | 0.08 |
| **1624** | A766V | 766 | A | V | 0.31 | 0.01 |
| **1625** | D559Y | 559 | D | Y | 0.31 | 0.01 |
| **1626** | E95T | 95 | E | T | 0.31 | 0.11 |
| **1627** | A139R | 139 | A | R | 0.31 | 0.33 |
| **1628** | D458T | 458 | D | T | 0.31 | 0.04 |
| **1629** | K804L | 804 | K | L | 0.31 | 0.11 |
| **1630** | Q941D | 941 | Q | D | 0.31 | 0.34 |
| **1631** | A920G | 920 | A | G | 0.31 | 0.18 |
| **1632** | Y381T | 381 | Y | T | 0.31 | 0.16 |
| **1633** | K692Y | 692 | K | Y | 0.31 | 0.01 |
| **1634** | D832E | 832 | D | E | 0.31 | 0.16 |
| **1635** | T246A | 246 | T | A | 0.31 | 0.11 |
| **1636** | Y237F | 237 | Y | F | 0.3 | 0.02 |
| **1637** | V842W | 842 | V | W | 0.3 | 0.32 |
| **1638** | G668L | 668 | G | L | 0.3 | 0.17 |
| **1639** | E695L | 695 | E | L | 0.3 | 0.11 |
| **1640** | L132R | 132 | L | R | 0.3 | 0.12 |
| **1641** | K775M | 775 | K | M | 0.3 | 0.24 |
| **1642** | R158K | 158 | R | K | 0.3 | 0.65 |
| **1643** | Y840L | 840 | Y | L | 0.3 | 0.3 |
| **1644** | W890G | 890 | W | G | 0.3 | 0.05 |
| **1645** | D437S | 437 | D | S | 0.3 | 0.47 |
| **1646** | E858N | 858 | E | N | 0.3 | 0.19 |
| **1647** | A1201S | 1201 | A | S | 0.3 | 0.64 |
| **1648** | L1192G | 1192 | L | G | 0.3 | 0.25 |
| **1649** | G576C | 576 | G | C | 0.3 | 0.2 |
| **1650** | D877A | 877 | D | A | 0.3 | 0.04 |
| **1651** | K1121M | 1121 | K | M | 0.3 | 0.1 |
| **1652** | K1079Q | 1079 | K | Q | 0.3 | 0.13 |
| **1653** | N157C | 157 | N | C | 0.3 | 0.34 |
| **1654** | K979M | 979 | K | M | 0.3 | 0.65 |
| **1655** | K199P | 199 | K | P | 0.3 | 0.14 |
| **1656** | L414K | 414 | L | K | 0.3 | 0.01 |
| **1657** | D297A | 297 | D | A | 0.3 | 0.07 |
| **1658** | L738C | 738 | L | C | 0.3 | 0.26 |
| **1659** | G475L | 475 | G | L | 0.3 | 0.21 |
| **1660** | V411S | 411 | V | S | 0.3 | 0.02 |
| **1661** | I318A | 318 | I | A | 0.3 | 1.18 |
| **1662** | G335L | 335 | G | L | 0.3 | 0.08 |
| **1663** | Y903S | 903 | Y | S | 0.3 | 0.2 |
| **1664** | K1026H | 1026 | K | H | 0.3 | 0.01 |
| **1665** | L914I | 914 | L | I | 0.3 | 0.01 |
| **1666** | N955C | 955 | N | C | 0.3 | 1.51 |
| **1667** | L1091R | 1091 | L | R | 0.3 | 0.07 |
| **1668** | N63Q | 63 | N | Q | 0.3 | 0.15 |
| **1669** | K1026D | 1026 | K | D | 0.3 | 0.19 |
| **1670** | S1020P | 1020 | S | P | 0.3 | 0.08 |
| **1671** | S1225W | 1225 | S | W | 0.3 | 0.05 |
| **1672** | M949R | 949 | M | R | 0.3 | 0.1 |
| **1673** | Y700R | 700 | Y | R | 0.3 | 0.54 |
| **1674** | N327L | 327 | N | L | 0.3 | 0.12 |
| **1675** | S982M | 982 | S | M | 0.3 | 0.09 |
| **1676** | F19C | 19 | F | C | 0.3 | 0 |
| **1677** | S780A | 780 | S | A | 0.3 | 0.01 |
| **1678** | L839A | 839 | L | A | 0.29 | 0.01 |
| **1679** | E981T | 981 | E | T | 0.29 | 0.21 |
| **1680** | D283L | 283 | D | L | 0.29 | 0.05 |
| **1681** | K269P | 269 | K | P | 0.29 | 0.46 |
| **1682** | E981S | 981 | E | S | 0.29 | 0.02 |
| **1683** | E88K | 88 | E | K | 0.29 | 0.02 |
| **1684** | R86V | 86 | R | V | 0.29 | 0.14 |
| **1685** | F81P | 81 | F | P | 0.29 | 0.17 |
| **1686** | V193Q | 193 | V | Q | 0.29 | 0.05 |
| **1687** | S1132C | 1132 | S | C | 0.29 | 0.7 |
| **1688** | K1003D | 1003 | K | D | 0.29 | 0.18 |
| **1689** | T480Y | 480 | T | Y | 0.29 | 0.06 |
| **1690** | T1016Y | 1016 | T | Y | 0.29 | 0.12 |
| **1691** | D283Q | 283 | D | Q | 0.29 | 0.34 |
| **1692** | N356F | 356 | N | F | 0.29 | 0.09 |
| **1693** | K372R | 372 | K | R | 0.29 | 0.44 |
| **1694** | T778I | 778 | T | I | 0.29 | 0.3 |
| **1695** | R82Y | 82 | R | Y | 0.29 | 0.05 |
| **1696** | F328A | 328 | F | A | 0.29 | 0.26 |
| **1697** | Y606E | 606 | Y | E | 0.29 | 0.09 |
| **1698** | L97A | 97 | L | A | 0.29 | 0.03 |
| **1699** | A1181R | 1181 | A | R | 0.29 | 0.32 |
| **1700** | K265Y | 265 | K | Y | 0.29 | 0 |
| **1701** | V574S | 574 | V | S | 0.29 | 0.54 |
| **1702** | R102N | 102 | R | N | 0.29 | 0.05 |
| **1703** | K536D | 536 | K | D | 0.29 | 0.39 |
| **1704** | D926A | 926 | D | A | 0.29 | 0.21 |
| **1705** | M1134Y | 1134 | M | Y | 0.29 | 0.11 |
| **1706** | L927R | 927 | L | R | 0.29 | 0.12 |
| **1707** | N1156T | 1156 | N | T | 0.29 | 0.24 |
| **1708** | L94Y | 94 | L | Y | 0.29 | 0.21 |
| **1709** | T480M | 480 | T | M | 0.29 | 0.18 |
| **1710** | F702R | 702 | F | R | 0.29 | 0.18 |
| **1711** | Y57D | 57 | Y | D | 0.29 | 0.14 |
| **1712** | L586I | 586 | L | I | 0.29 | 0.08 |
| **1713** | K120Q | 120 | K | Q | 0.29 | 0.06 |
| **1714** | Y183S | 183 | Y | S | 0.29 | 0.11 |
| **1715** | G697A | 697 | G | A | 0.29 | 0.14 |
| **1716** | N849H | 849 | N | H | 0.29 | 0.83 |
| **1717** | K120T | 120 | K | T | 0.29 | 0.09 |
| **1718** | K320Q | 320 | K | Q | 0.29 | 0.1 |
| **1719** | G588L | 588 | G | L | 0.29 | 0.01 |
| **1720** | L498K | 498 | L | K | 0.29 | 0.09 |
| **1721** | N772P | 772 | N | P | 0.29 | 0.54 |
| **1722** | Y1221L | 1221 | Y | L | 0.29 | 0.12 |
| **1723** | R1144P | 1144 | R | P | 0.29 | 0.11 |
| **1724** | I859L | 859 | I | L | 0.29 | 0.18 |
| **1725** | F227Q | 227 | F | Q | 0.29 | 0.01 |
| **1726** | L97H | 97 | L | H | 0.29 | 0.21 |
| **1727** | V491R | 491 | V | R | 0.29 | 0.07 |
| **1728** | Y1167L | 1167 | Y | L | 0.29 | 1.5 |
| **1729** | N582Q | 582 | N | Q | 0.29 | 0.03 |
| **1730** | Y956F | 956 | Y | F | 0.29 | 0.53 |
| **1731** | T891R | 891 | T | R | 0.29 | 0.18 |
| **1732** | N772C | 772 | N | C | 0.29 | 0.44 |
| **1733** | S432G | 432 | S | G | 0.29 | 0.04 |
| **1734** | F789V | 789 | F | V | 0.29 | 0.07 |
| **1735** | K272R | 272 | K | R | 0.29 | 0.06 |
| **1736** | E695Y | 695 | E | Y | 0.29 | 0.06 |
| **1737** | F473C | 473 | F | C | 0.29 | 0.03 |
| **1738** | K1050Y | 1050 | K | Y | 0.28 | 0.03 |
| **1739** | N582H | 582 | N | H | 0.28 | 0.11 |
| **1740** | G488A | 488 | G | A | 0.28 | 0.11 |
| **1741** | G291W | 291 | G | W | 0.28 | 0.11 |
| **1742** | D156A | 156 | D | A | 0.28 | 0.11 |
| **1743** | F131D | 131 | F | D | 0.28 | 0.05 |
| **1744** | K1015N | 1015 | K | N | 0.28 | 0.1 |
| **1745** | I911C | 911 | I | C | 0.28 | 0.05 |
| **1746** | F1198M | 1198 | F | M | 0.28 | 0.15 |
| **1747** | S929I | 929 | S | I | 0.28 | 0.14 |
| **1748** | N1081C | 1081 | N | C | 0.28 | 0.07 |
| **1749** | F144R | 144 | F | R | 0.28 | 0.04 |
| **1750** | K265E | 265 | K | E | 0.28 | 0.06 |
| **1751** | K265F | 265 | K | F | 0.28 | 0.05 |
| **1752** | E330H | 330 | E | H | 0.28 | 0.02 |
| **1753** | E217V | 217 | E | V | 0.28 | 0.19 |
| **1754** | I1111P | 1111 | I | P | 0.28 | 0.11 |
| **1755** | A998R | 998 | A | R | 0.28 | 1.08 |
| **1756** | N772T | 772 | N | T | 0.28 | 0.15 |
| **1757** | F151N | 151 | F | N | 0.28 | 0.06 |
| **1758** | N590K | 590 | N | K | 0.28 | 0.2 |
| **1759** | S185G | 185 | S | G | 0.28 | 0.21 |
| **1760** | T1019F | 1019 | T | F | 0.28 | 0.08 |
| **1761** | E815W | 815 | E | W | 0.28 | 0.25 |
| **1762** | F474D | 474 | F | D | 0.28 | 0.13 |
| **1763** | W1218F | 1218 | W | F | 0.28 | 0.65 |
| **1764** | N1081F | 1081 | N | F | 0.28 | 0.11 |
| **1765** | D1146E | 1146 | D | E | 0.28 | 0.2 |
| **1766** | Y294M | 294 | Y | M | 0.28 | 0.06 |
| **1767** | N1182Q | 1182 | N | Q | 0.28 | 0.28 |
| **1768** | I496M | 496 | I | M | 0.28 | 0.04 |
| **1769** | Y562H | 562 | Y | H | 0.28 | 0.11 |
| **1770** | Y616C | 616 | Y | C | 0.28 | 0.15 |
| **1771** | Y1124E | 1124 | Y | E | 0.28 | 0.12 |
| **1772** | S296D | 296 | S | D | 0.28 | 0.02 |
| **1773** | G830I | 830 | G | I | 0.28 | 1.5 |
| **1774** | S1132G | 1132 | S | G | 0.28 | 0.47 |
| **1775** | E130W | 130 | E | W | 0.28 | 0.14 |
| **1776** | T814H | 814 | T | H | 0.28 | 0.08 |
| **1777** | N1178S | 1178 | N | S | 0.28 | 0.1 |
| **1778** | D541E | 541 | D | E | 0.28 | 0.12 |
| **1779** | T85L | 85 | T | L | 0.28 | 0.05 |
| **1780** | G902L | 902 | G | L | 0.28 | 0.18 |
| **1781** | R182S | 182 | R | S | 0.28 | 0.08 |
| **1782** | D919A | 919 | D | A | 0.28 | 0.12 |
| **1783** | D1032E | 1032 | D | E | 0.28 | 0.06 |
| **1784** | Y494K | 494 | Y | K | 0.28 | 0.02 |
| **1785** | S14M | 14 | S | M | 0.28 | 0.11 |
| **1786** | E379Y | 379 | E | Y | 0.28 | 0.21 |
| **1787** | E302P | 302 | E | P | 0.28 | 0.21 |
| **1788** | N651D | 651 | N | D | 0.28 | 0.19 |
| **1789** | N577L | 577 | N | L | 0.28 | 0.01 |
| **1790** | D407P | 407 | D | P | 0.28 | 0.26 |
| **1791** | N481Q | 481 | N | Q | 0.28 | 0.22 |
| **1792** | E90R | 90 | E | R | 0.28 | 0.09 |
| **1793** | I643S | 643 | I | S | 0.28 | 0.09 |
| **1794** | I736K | 736 | I | K | 0.28 | 1.5 |
| **1795** | E207A | 207 | E | A | 0.28 | 0.07 |
| **1796** | I831M | 831 | I | M | 0.28 | 0.2 |
| **1797** | Y524G | 524 | Y | G | 0.28 | 0.4 |
| **1798** | K1017V | 1017 | K | V | 0.28 | 0.1 |
| **1799** | A150I | 150 | A | I | 0.28 | 0.29 |
| **1800** | K110R | 110 | K | R | 0.28 | 0.09 |
| **1801** | R867T | 867 | R | T | 0.28 | 1.19 |
| **1802** | I189Q | 189 | I | Q | 0.28 | 0.03 |
| **1803** | L1192N | 1192 | L | N | 0.28 | 0.05 |
| **1804** | I196G | 196 | I | G | 0.28 | 0.11 |
| **1805** | K1017P | 1017 | K | P | 0.28 | 0.33 |
| **1806** | I831R | 831 | I | R | 0.28 | 1.19 |
| **1807** | T1019L | 1019 | T | L | 0.28 | 0.1 |
| **1808** | S212R | 212 | S | R | 0.28 | 0.2 |
| **1809** | K538Y | 538 | K | Y | 0.28 | 0.41 |
| **1810** | A556S | 556 | A | S | 0.28 | 0.1 |
| **1811** | P528N | 528 | P | N | 0.28 | 0.07 |
| **1812** | G254K | 254 | G | K | 0.28 | 0.21 |
| **1813** | T230Y | 230 | T | Y | 0.28 | 0.18 |
| **1814** | K1015A | 1015 | K | A | 0.28 | 0.03 |
| **1815** | G475P | 475 | G | P | 0.28 | 0.06 |
| **1816** | S987Q | 987 | S | Q | 0.27 | 0.5 |
| **1817** | E467I | 467 | E | I | 0.27 | 0.52 |
| **1818** | A108C | 108 | A | C | 0.27 | 0.3 |
| **1819** | I184K | 184 | I | K | 0.27 | 0.13 |
| **1820** | E579G | 579 | E | G | 0.27 | 0 |
| **1821** | W1063Y | 1063 | W | Y | 0.27 | 0.21 |
| **1822** | L140R | 140 | L | R | 0.27 | 0.03 |
| **1823** | K595I | 595 | K | I | 0.27 | 0.22 |
| **1824** | F81K | 81 | F | K | 0.27 | 0.14 |
| **1825** | E299M | 299 | E | M | 0.27 | 0.21 |
| **1826** | D1023R | 1023 | D | R | 0.27 | 0.21 |
| **1827** | P997I | 997 | P | I | 0.27 | 0.21 |
| **1828** | F669V | 669 | F | V | 0.27 | 0.11 |
| **1829** | L744D | 744 | L | D | 0.27 | 0.21 |
| **1830** | L498F | 498 | L | F | 0.27 | 0.09 |
| **1831** | K1079I | 1079 | K | I | 0.27 | 0.05 |
| **1832** | E539K | 539 | E | K | 0.27 | 0.38 |
| **1833** | F328V | 328 | F | V | 0.27 | 0.05 |
| **1834** | I557H | 557 | I | H | 0.27 | 0.05 |
| **1835** | L261D | 261 | L | D | 0.27 | 0.06 |
| **1836** | I62S | 62 | I | S | 0.27 | 0.01 |
| **1837** | N837Q | 837 | N | Q | 0.27 | 0.19 |
| **1838** | T778A | 778 | T | A | 0.27 | 0.08 |
| **1839** | E479V | 479 | E | V | 0.27 | 0.02 |
| **1840** | K1025H | 1025 | K | H | 0.27 | 0.05 |
| **1841** | I234R | 234 | I | R | 0.27 | 0.04 |
| **1842** | S609T | 609 | S | T | 0.27 | 0.23 |
| **1843** | M1035H | 1035 | M | H | 0.27 | 0.05 |
| **1844** | Y494R | 494 | Y | R | 0.27 | 0.07 |
| **1845** | D122P | 122 | D | P | 0.27 | 0.05 |
| **1846** | F530C | 530 | F | C | 0.27 | 0.19 |
| **1847** | F627H | 627 | F | H | 0.27 | 0.21 |
| **1848** | Y262V | 262 | Y | V | 0.27 | 0.07 |
| **1849** | I392R | 392 | I | R | 0.27 | 0.29 |
| **1850** | V1037M | 1037 | V | M | 0.27 | 0.34 |
| **1851** | C805N | 805 | C | N | 0.27 | 0.01 |
| **1852** | Y214T | 214 | Y | T | 0.27 | 0 |
| **1853** | E683Y | 683 | E | Y | 0.27 | 0.01 |
| **1854** | I736T | 736 | I | T | 0.27 | 0.65 |
| **1855** | D297E | 297 | D | E | 0.27 | 0.1 |
| **1856** | I418T | 418 | I | T | 0.27 | 0.2 |
| **1857** | T814R | 814 | T | R | 0.27 | 0.13 |
| **1858** | L856S | 856 | L | S | 0.27 | 0.19 |
| **1859** | K427C | 427 | K | C | 0.27 | 0.09 |
| **1860** | A901D | 901 | A | D | 0.27 | 0.49 |
| **1861** | K391Q | 391 | K | Q | 0.27 | 0.26 |
| **1862** | V783F | 783 | V | F | 0.27 | 0.18 |
| **1863** | G335E | 335 | G | E | 0.27 | 0.25 |
| **1864** | K917Q | 917 | K | Q | 0.27 | 0.11 |
| **1865** | E795C | 795 | E | C | 0.27 | 0.17 |
| **1866** | F197E | 197 | F | E | 0.27 | 0.27 |
| **1867** | F1027D | 1027 | F | D | 0.27 | 0.16 |
| **1868** | L1150F | 1150 | L | F | 0.27 | 0.27 |
| **1869** | S686Q | 686 | S | Q | 0.26 | 0.44 |
| **1870** | L37D | 37 | L | D | 0.26 | 0.32 |
| **1871** | T480H | 480 | T | H | 0.26 | 0.14 |
| **1872** | E743Q | 743 | E | Q | 0.26 | 0.14 |
| **1873** | K107L | 107 | K | L | 0.26 | 0.01 |
| **1874** | N808P | 808 | N | P | 0.26 | 0 |
| **1875** | K564S | 564 | K | S | 0.26 | 0.09 |
| **1876** | N803H | 803 | N | H | 0.26 | 0.11 |
| **1877** | S1020F | 1020 | S | F | 0.26 | 0.01 |
| **1878** | I1104T | 1104 | I | T | 0.26 | 0.49 |
| **1879** | N306W | 306 | N | W | 0.26 | 0.05 |
| **1880** | D1120E | 1120 | D | E | 0.26 | 0.03 |
| **1881** | D1146Q | 1146 | D | Q | 0.26 | 0.08 |
| **1882** | R1073Y | 1073 | R | Y | 0.26 | 0.34 |
| **1883** | Y872S | 872 | Y | S | 0.26 | 0.01 |
| **1884** | Y429H | 429 | Y | H | 0.26 | 0.33 |
| **1885** | D423R | 423 | D | R | 0.26 | 0.2 |
| **1886** | K785I | 785 | K | I | 0.26 | 0.54 |
| **1887** | E299A | 299 | E | A | 0.26 | 0.14 |
| **1888** | W1218Q | 1218 | W | Q | 0.26 | 0.13 |
| **1889** | G846Y | 846 | G | Y | 0.26 | 0.39 |
| **1890** | A761R | 761 | A | R | 0.26 | 0.88 |
| **1891** | K940S | 940 | K | S | 0.26 | 0.01 |
| **1892** | N1081L | 1081 | N | L | 0.26 | 0.28 |
| **1893** | S1225F | 1225 | S | F | 0.26 | 0.06 |
| **1894** | I318L | 318 | I | L | 0.26 | 0.1 |
| **1895** | F980V | 980 | F | V | 0.26 | 0.11 |
| **1896** | P826D | 826 | P | D | 0.26 | 0.15 |
| **1897** | E981L | 981 | E | L | 0.26 | 0.27 |
| **1898** | S1132L | 1132 | S | L | 0.26 | 0.01 |
| **1899** | T777W | 777 | T | W | 0.26 | 0.1 |
| **1900** | A604Q | 604 | A | Q | 0.26 | 0.13 |
| **1901** | D283K | 283 | D | K | 0.26 | 0.54 |
| **1902** | D637K | 637 | D | K | 0.26 | 0.1 |
| **1903** | K72Y | 72 | K | Y | 0.26 | 0.02 |
| **1904** | E130A | 130 | E | A | 0.26 | 1 |
| **1905** | T1145E | 1145 | T | E | 0.26 | 0.44 |
| **1906** | N803T | 803 | N | T | 0.26 | 0.01 |
| **1907** | F225C | 225 | F | C | 0.26 | 0.19 |
| **1908** | E925S | 925 | E | S | 0.26 | 0.14 |
| **1909** | N356H | 356 | N | H | 0.26 | 0.11 |
| **1910** | S79F | 79 | S | F | 0.26 | 0.39 |
| **1911** | N1156H | 1156 | N | H | 0.26 | 0 |
| **1912** | I831S | 831 | I | S | 0.26 | 0.19 |
| **1913** | Q853M | 853 | Q | M | 0.26 | 0.26 |
| **1914** | T540Q | 540 | T | Q | 0.26 | 0 |
| **1915** | A1094D | 1094 | A | D | 0.26 | 0.27 |
| **1916** | E232R | 232 | E | R | 0.26 | 0.16 |
| **1917** | G242L | 242 | G | L | 0.26 | 0.34 |
| **1918** | K1079L | 1079 | K | L | 0.26 | 0.29 |
| **1919** | M1137R | 1137 | M | R | 0.26 | 0.09 |
| **1920** | I996G | 996 | I | G | 0.26 | 0.11 |
| **1921** | M603A | 603 | M | A | 0.26 | 0.02 |
| **1922** | S1132F | 1132 | S | F | 0.26 | 0.57 |
| **1923** | V38L | 38 | V | L | 0.26 | 0.24 |
| **1924** | V908M | 908 | V | M | 0.26 | 0.16 |
| **1925** | D122V | 122 | D | V | 0.26 | 0.13 |
| **1926** | L856V | 856 | L | V | 0.26 | 0.07 |
| **1927** | I904L | 904 | I | L | 0.26 | 0.04 |
| **1928** | W1063D | 1063 | W | D | 0.26 | 0.16 |
| **1929** | F1149I | 1149 | F | I | 0.26 | 0.1 |
| **1930** | R883T | 883 | R | T | 0.26 | 0 |
| **1931** | C1116E | 1116 | C | E | 0.26 | 0.07 |
| **1932** | Y494M | 494 | Y | M | 0.26 | 0 |
| **1933** | D407R | 407 | D | R | 0.26 | 0.07 |
| **1934** | K89V | 89 | K | V | 0.26 | 0.02 |
| **1935** | Q735P | 735 | Q | P | 0.26 | 0.69 |
| **1936** | K1017M | 1017 | K | M | 0.26 | 0.06 |
| **1937** | A1022H | 1022 | A | H | 0.26 | 0.26 |
| **1938** | S739C | 739 | S | C | 0.26 | 0.04 |
| **1939** | H759K | 759 | H | K | 0.26 | 0.54 |
| **1940** | Y11D | 11 | Y | D | 0.26 | 0.06 |
| **1941** | I138K | 138 | I | K | 0.25 | 0.11 |
| **1942** | I1111Q | 1111 | I | Q | 0.25 | 0.12 |
| **1943** | R1144G | 1144 | R | G | 0.25 | 0.18 |
| **1944** | I800L | 800 | I | L | 0.25 | 0.15 |
| **1945** | D1148E | 1148 | D | E | 0.25 | 0.17 |
| **1946** | D40N | 40 | D | N | 0.25 | 0.06 |
| **1947** | G1196F | 1196 | G | F | 0.25 | 0.01 |
| **1948** | V844R | 844 | V | R | 0.25 | 0.32 |
| **1949** | L459Q | 459 | L | Q | 0.25 | 0.01 |
| **1950** | D32E | 32 | D | E | 0.25 | 0.02 |
| **1951** | E382F | 382 | E | F | 0.25 | 0.16 |
| **1952** | S394P | 394 | S | P | 0.25 | 0.08 |
| **1953** | N928C | 928 | N | C | 0.25 | 0.24 |
| **1954** | H759P | 759 | H | P | 0.25 | 0.14 |
| **1955** | Y854D | 854 | Y | D | 0.25 | 0.03 |
| **1956** | D423Q | 423 | D | Q | 0.25 | 0.25 |
| **1957** | N356T | 356 | N | T | 0.25 | 0.1 |
| **1958** | D708E | 708 | D | E | 0.25 | 0.12 |
| **1959** | N864F | 864 | N | F | 0.25 | 0.1 |
| **1960** | T1019Y | 1019 | T | Y | 0.25 | 0.09 |
| **1961** | R86M | 86 | R | M | 0.25 | 0.04 |
| **1962** | F131C | 131 | F | C | 0.25 | 0.15 |
| **1963** | N1082H | 1082 | N | H | 0.25 | 0.09 |
| **1964** | D40S | 40 | D | S | 0.25 | 2.12 |
| **1965** | D461A | 461 | D | A | 0.25 | 0.22 |
| **1966** | E88H | 88 | E | H | 0.25 | 0.11 |
| **1967** | A766N | 766 | A | N | 0.25 | 0.44 |
| **1968** | V491S | 491 | V | S | 0.25 | 0.07 |
| **1969** | E330R | 330 | E | R | 0.25 | 0.16 |
| **1970** | F173V | 173 | F | V | 0.25 | 0.11 |
| **1971** | Q567S | 567 | Q | S | 0.25 | 0.49 |
| **1972** | V574P | 574 | V | P | 0.25 | 0 |
| **1973** | K413C | 413 | K | C | 0.25 | 0.54 |
| **1974** | D535A | 535 | D | A | 0.25 | 0.11 |
| **1975** | P964Q | 964 | P | Q | 0.25 | 0.05 |
| **1976** | S143K | 143 | S | K | 0.25 | 0.01 |
| **1977** | L751H | 751 | L | H | 0.25 | 0.44 |

### Additional file 2: Table S2. T-DNA vectors used in this study

| **Vector #** | **Target site** | **crRNA clone** | **Cas12a** | **Expression vector** |
| --- | --- | --- | --- | --- |
| **1134** | L9 | pYPQ141-ZmUbi-RZ-Lb-L9 | LbCas12a | pYPQ203 |
| **3873** | GG1 | pYPQ141-ZmUbi-RZ-Lb-GG1 | LbCas12a | pYPQ203 |
| **3874** | AA1 | pYPQ141-ZmUbi-RZ-Lb-AA1 | LbCas12a | pYPQ203 |
| **3875** | GC2 | pYPQ141-ZmUbi-RZ-Lb-GC2 | LbCas12a | pYPQ203 |
| **3876** | GA1 | pYPQ141-ZmUbi-RZ-Lb-GA1 | LbCas12a | pYPQ203 |
| **3877** | GC1 | pYPQ141-ZmUbi-RZ-Lb-GC1 | LbCas12a | pYPQ203 |
| **2689** | L9 | pYPQ141-ZmUbi-RZ-Lb-L9 | Lb-D156RCas12a | pYPQ203 |
| **3879** | GG1 | pYPQ141-ZmUbi-RZ-Lb-GG1 | Lb-D156RCas12a | pYPQ203 |
| **3880** | AA1 | pYPQ141-ZmUbi-RZ-Lb-AA1 | Lb-D156RCas12a | pYPQ203 |
| **3881** | GC2 | pYPQ141-ZmUbi-RZ-Lb-GC2 | Lb-D156RCas12a | pYPQ203 |
| **3882** | GA1 | pYPQ141-ZmUbi-RZ-Lb-GA1 | Lb-D156RCas12a | pYPQ203 |
| **3883** | GC1 | pYPQ141-ZmUbi-RZ-Lb-GC1 | Lb-D156RCas12a | pYPQ203 |
| **3903** | L9 | pYPQ141-ZmUbi-RZ-Lb-L9 | LbCas12a-RVQ | pYPQ203 |
| **3905** | GG1 | pYPQ141-ZmUbi-RZ-Lb-GG1 | LbCas12a-RVQ | pYPQ203 |
| **3906** | AA1 | pYPQ141-ZmUbi-RZ-Lb-AA1 | LbCas12a-RVQ | pYPQ203 |
| **3907** | GC2 | pYPQ141-ZmUbi-RZ-Lb-GC2 | LbCas12a-RVQ | pYPQ203 |
| **3908** | GA1 | pYPQ141-ZmUbi-RZ-Lb-GA1 | LbCas12a-RVQ | pYPQ203 |
| **3909** | GC1 | pYPQ141-ZmUbi-RZ-Lb-GC1 | LbCas12a-RVQ | pYPQ203 |
| **3910** | L9 | pYPQ141-ZmUbi-RZ-Lb-L9 | LbCas12a-RRVQ | pYPQ203 |
| **3912** | GG1 | pYPQ141-ZmUbi-RZ-Lb-GG1 | LbCas12a-RRVQ | pYPQ203 |
| **3913** | AA1 | pYPQ141-ZmUbi-RZ-Lb-AA1 | LbCas12a-RRVQ | pYPQ203 |
| **3914** | GC2 | pYPQ141-ZmUbi-RZ-Lb-GC2 | LbCas12a-RRVQ | pYPQ203 |
| **3915** | GA1 | pYPQ141-ZmUbi-RZ-Lb-GA1 | LbCas12a-RRVQ | pYPQ203 |
| **3916** | GC1 | pYPQ141-ZmUbi-RZ-Lb-GC1 | LbCas12a-RRVQ | pYPQ203 |
| **4317** | AA1, GC2,GA1,GC1 | pYPQ144-ZmUbi-LowAct-Lb | LbCas12a | pYPQ203 |
| **4318** | AA1, GC2,GA1,GC1 | pYPQ144-ZmUbi-LowAct-Lb | Lb-D156RCas12a | pYPQ203 |
| **4319** | AA1, GC2,GA1,GC1 | pYPQ144-ZmUbi-LowAct-Lb | LbCas12a-RVQ | pYPQ203 |
| **4320** | AA1, GC2,GA1,GC1 | pYPQ144-ZmUbi-LowAct-Lb | LbCas12a-RRVQ | pYPQ203 |
| **4351** | 4CL1-1,2,Pll1,2, SVP1,2 | pYPQ146-AtUBQ10-Pt-6crRNA-Lb | LbCas12a | pYPQ202 |
| **4352** | 4CL1-1,2,Pll1,2, SVP1,2 | pYPQ146-AtUBQ10-Pt-6crRNA-Lb | Lb-D156RCas12a | pYPQ202 |
| **4353** | 4CL1-1,2,Pll1,2, SVP1,2 | pYPQ146-AtUBQ10-Pt-6crRNA-Lb | LbCas12a-RVQ | pYPQ202 |
| **4354** | 4CL1-1,2,Pll1,2, SVP1,2 | pYPQ146-AtUBQ10-Pt-6crRNA-Lb | LbCas12a-RRVQ | pYPQ202 |

### Additional file 2: Table S3. Oligos used in this study

| Name | 5'-3' sequence | Usage |
| --- | --- | --- |
| 4CL1-crRNA1-F | TAGATGCAACCCTGTAGTCCCTGATGAA | crRNA cloning |
| 4CL1-crRNA1-R | GGCCTTCATCAGGGACTACAGGGTTGCA | crRNA cloning |
| 4CL1-crRNA2-F | TAGATCCAAGGAACCATTCGACATAAAA | crRNA cloning |
| 4CL1-crRNA2-R | GGCCTTTTATGTCGAATGGTTCCTTGGA | crRNA cloning |
| Pll-crRNA1-F | TAGATAAACCTCTCAAGTTGAAGAATTC | crRNA cloning |
| Pll-crRNA1-R | GGCCGAATTCTTCAACTTGAGAGGTTTA | crRNA cloning |
| Pll-crRNA2-F | TAGATAACCACCTTGAGCCCCAAAGCCT | crRNA cloning |
| 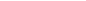Pll-crRNA2-R | GGCCAGGCTTTGGGGCTCAAGGTGGTTA | crRNA cloning |
| SVP-crRNA1-F | TAGATCACTCGAAGAATCTTGAGAAGCT | crRNA cloning |
| SVP-crRNA1-R | GGCCAGCTTCTCAAGATTCTTCGAGTGA | crRNA cloning |
| SVP-crRNA2-F | TAGATTCCGCAACTTCCTTACTCAACCT | crRNA cloning |
| SVP-crRNA2-R | GGCCAGGTTGAGTAAGGAAGTTGCGGAA | crRNA cloning |
| Hi-TOM-F-1 | ACACTCTTTCCCTACACGACGCTCTTCCGATCTgcttGCGTtggagtgagtacggtgtgc | Hi-TOM barcoding primer |
| Hi-TOM-F-2 | ACACTCTTTCCCTACACGACGCTCTTCCGATCTgcttGTAGtggagtgagtacggtgtgc | Hi-TOM barcoding primer |
| Hi-TOM-F-3 | ACACTCTTTCCCTACACGACGCTCTTCCGATCTgcttACGCtggagtgagtacggtgtgc | Hi-TOM barcoding primer |
| Hi-TOM-F-4 | ACACTCTTTCCCTACACGACGCTCTTCCGATCTgcttCTCGtggagtgagtacggtgtgc | Hi-TOM barcoding primer |
| Hi-TOM-F-5 | ACACTCTTTCCCTACACGACGCTCTTCCGATCTgcttGCTCtggagtgagtacggtgtgc | Hi-TOM barcoding primer |
| Hi-TOM-F-6 | ACACTCTTTCCCTACACGACGCTCTTCCGATCTgcttAGTCtggagtgagtacggtgtgc | Hi-TOM barcoding primer |
| Hi-TOM-F-7 | ACACTCTTTCCCTACACGACGCTCTTCCGATCTgcttCGACtggagtgagtacggtgtgc | Hi-TOM barcoding primer |
| 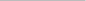Hi-TOM-F-8 | ACACTCTTTCCCTACACGACGCTCTTCCGATCTgcttGATGtggagtgagtacggtgtgc | Hi-TOM barcoding primer |
| Hi-TOM-F-9 | ACACTCTTTCCCTACACGACGCTCTTCCGATCTgcttATACtggagtgagtacggtgtgc | Hi-TOM barcoding primer |
| Hi-TOM-F-10 | ACACTCTTTCCCTACACGACGCTCTTCCGATCTgcttCACAtggagtgagtacggtgtgc | Hi-TOM barcoding primer |
| Hi-TOM-F-11 | ACACTCTTTCCCTACACGACGCTCTTCCGATCTgcttGTGCtggagtgagtacggtgtgc | Hi-TOM barcoding primer |
| Hi-TOM-F-1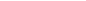2 | ACACTCTTTCCCTACACGACGCTCTTCCGATCTgcttACTAtggagtgagtacggtgtgc | Hi-TOM barcoding primer |
| Hi-TOM-R-A | GACTGGAGTTCAGACGTGTGCTCTTCCGATCTctgtGCGTtgagttggatgctggatgg | Hi-TOM barcoding primer |
| Hi-TOM-R-B | GACTGGAGTTCAGACGTGTGCTCTTCCGATCTctgtGTAGtgagttggatgctggatgg | Hi-TOM barcoding primer |
| Hi-TOM-R-C | GACTGGAGTTCAGACGTGTGCTCTTCCGATCTctgtACGCtgagttggatgctggatgg | Hi-TOM barcoding primer |
| Hi-TOM-R-D | GACTGGAGTTCAGACGTGTGCTCTTCCGATCTctgtCTCGtgagttggatgctggatgg | Hi-TOM barcoding primer |
| Hi-TOM-R-E | GACTGGAGTTCAGACGTGTGCTCTTCCGATCTctgtGCTCtgagttggatgctggatgg | Hi-TOM barcoding primer |
| Hi-TOM-R-F | GACTGGAGTTCAGACGTGTGCTCTTCCGATCTctgtAGTCtgagttggatgctggatgg | Hi-TOM barcoding primer |
| Hi-TOM-R-G | GACTGGAGTTCAGACGTGTGCTCTTCCGATCTctgtCGACtgagttggatgctggatgg | Hi-TOM barcoding primer |
| Hi-TOM-R-H | GACTGGAGTTCAGACGTGTGCTCTTCCGATCTctgtGATGtgagttggatgctggatgg | Hi-TOM barcoding primer |
| 4CL1-Hitom-F3 | ggagtgagtacggtgtgcGGGTGCTTGCACTTTTCAGA | Hi-TOM barcoding primer |
| 4CL1-Hitom-R3 | gagttggatgctggatggCTCCATCTACCTGTTGAGCCA | Hi-TOM barcoding primer |
| 4CL1-Hitom-F4 | ggagtgagtacggtgtgcGGGATATGGAATGACCGAGG | Hi-TOM barcoding primer |
| 4CL1-Hitom-R4 | gagttggatgctggatggTCATGATCTGATCACCCCGG | Hi-TOM barcoding primer |
| PII-Hitom-F1 | ggagtgagtacggtgtgcAAACCAGGCTCCCTCACTTC | Hi-TOM barcoding primer |
| PII-Hitom-R1 | gagttggatgctggatggCGCATACCAGGAACAGAGGA | Hi-TOM barcoding primer |
| PII-Hitom-F2 | ggagtgagtacggtgtgcGGCTCTGTTGAAAATTGGTATTCG | Hi-TOM barcoding primer |
| 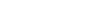PII-Hitom-R2 | gagttggatgctggatggGCGAAAGGAGACGAAACAGAA | Hi-TOM barcoding primer |
| SVP-Hitom-F1 | ggagtgagtacggtgtgcCTTCCAGGCCATCTTAGGAAATGA | Hi-TOM barcoding primer |
| SVP-Hitom-R1 | gagttggatgctggatggGCACTGTCTTAAACACTCTCCAC | Hi-TOM barcoding primer |
| SVP-Hitom-F2 | ggagtgagtacggtgtgcCCTGACTTCTTTTACATTAGCTGTGG | Hi-TOM barcoding primer |
| SVP-Hitom-R2 | gagttggatgctggatggAGGCATAGAGCTGCTAGAAGC | Hi-TOM barcoding primer |
| Hi-TOM-L9-F | ggagtgagtacggtgtgcGCACCAGCCTCCGATTTAT | Hi-TOM barcoding primer |
| Hi-TOM-L9-R | gagttggatgctggatggGCAAAGGGGTACCTGAGATG | Hi-TOM barcoding primer |
| Hi-TOM-GG1-F | ggagtgagtacggtgtgcTATAAGCCACCGATCCCACA | Hi-TOM barcoding primer |
| Hi-TOM-GG1-R | gagttggatgctggatggGCATTATTGAAGGCTCGTTCC | Hi-TOM barcoding primer |
| GG1-TTV-HiTom-F1 | ggagtgagtacggtgtgcCTGACCCAACAACAGCCAAA | Hi-TOM barcoding primer |
| GG1-TTV-HiTom-R2 | gagttggatgctggatggGCATCATTTAGCCAGGGATC | Hi-TOM barcoding primer |
| AA1-TTV-Hitom-F1 | ggagtgagtacggtgtgcCCACATTTGTCATGGTTCTGA | Hi-TOM barcoding primer |
| AA1-TTV-Hitom-R1 | gagttggatgctggatggGGTTGCTGGTATTGCTGGAT | Hi-TOM barcoding primer |
| AA1-TTTV-Hitom-F1 | ggagtgagtacggtgtgcCGCGTTTACCATGGTTCTG | Hi-TOM barcoding primer |
| AA1-TTTV-Hitom-R1 | gagttggatgctggatggGGCTGCTGGTATTGTTGGAT | Hi-TOM barcoding primer |
| GC2-TTV-Hitom-F1 | ggagtgagtacggtgtgcCAAAACAAAAATCGACAGATTTCA | Hi-TOM barcoding primer |
| GC2-TTV-Hitom-R1 | gagttggatgctggatggAAATCTTGACAAATCAAACTATGCT | Hi-TOM barcoding primer |
| GC2-TTTV-Hitom-F1 | ggagtgagtacggtgtgctctgcaaggatcagttaccttt | Hi-TOM barcoding primer |
| GC2-TTTV-Hitom-R1 | gagttggatgctggatggacgccccttatcatcacttg | Hi-TOM barcoding primer |
| GA1-TTV-Hitom-F1 | ggagtgagtacggtgtgcAAGGGAACGGAAGGAGTAGC | Hi-TOM barcoding primer |
| GA1-TTV-Hitom-R1 | gagttggatgctggatggTCCTCAAAACGCTCTCAAGC | Hi-TOM barcoding primer |
| GA1-TTTV-Hitom-F1 | ggagtgagtacggtgtgccaagctccttgaacgaaagc | Hi-TOM barcoding primer |
| GA1-TTTV-Hitom-R1 | gagttggatgctggatgggccacatggcgctctaataa | Hi-TOM barcoding primer |
| GC1-TTV-Hitom-F1 | ggagtgagtacggtgtgcTATTTGGGAGCACTGCTTGG | Hi-TOM barcoding primer |
| GC1-TTV-Hitom-R1 | gagttggatgctggatggGCCCAACTCCTCATCAATGT | Hi-TOM barcoding primer |
| GC1-TTTV-Hitom-F1 | ggagtgagtacggtgtgcCACGTCCATTGCGCATATTA | Hi-TOM barcoding primer |
| GC1-TTTV-Hitom-R1 | gagttggatgctggatggGATGGACATGCTCATGATGG | Hi-TOM barcoding primer |
| HPRT-1 | auagucuuuccuugggugugu | HPRT Target site 1 |
| HPRT-2 | cuuggguguguuaaaagugac | HPRT Target site 2 |
| HPRT-3 | acacacccaaggaaagacuau | HPRT Target site 3 |
| HPRT-4 | uaauuaacagcuugcugguga | HPRT Target site 4 |
| HPRT-5 | uaaacacuguuucauuucauc | HPRT Target site 5 |
| HPRT-6 | gaaacgucagucuucucuuuu | HPRT Target site 6 |
| HPRT-7 | uaaugcccuguagucucucug | HPRT Target site 7 |
| HPRT-8 | auccgugcugaguguaccaug | HPRT Target site 8 |
| HPRT-9 | gguuaaagaugguuaaaugau | HPRT Target site 9 |
| HPRT-10 | ugugaaauggcuuauaauugc | HPRT Target site 10 |
| HPRT-11 | guuguuggauuugaaauucca | HPRT Target site 11 |
| HPRT-FWD | AAGAATGTTGTGATAAAAGGTGATGCT | T7EI primer for HPRT region |
| HPRT-RWD | ACACATCCATGGGACTTCTGCCTC | T7EI primer for HPRT region |
| Lib_1 | GCTATCTAGTGCTCAGTACGC(N:25252525)(N)(N)(N)TTTAAGGAAACTTCTGGCAGGACCAGGTCATGagatcggaagagcacacgtctgaactccagtca | Spec-seq library |
| Lib_2 | GCTATCTAGTGCTCAGTACGCACTG(N:25252525)(N)(N)(N)AGGAAACTTCTGGCAGGACCAGGTCATGagatcggaagagcacacgtctgaactccagtca | Spec-seq library |
| Lib_3 | GCTATCTAGTGCTCAGTACGCACTGTTTA(N:25252525)(N)(N)(N)AACTTCTGGCAGGACCAGGTCATGagatcggaagagcacacgtctgaactccagtca | Spec-seq library |
| Lib_4 | GCTATCTAGTGCTCAGTACGCACTGTTTAAGGA(N:25252525)(N)(N)(N)TCTGGCAGGACCAGGTCATGagatcggaagagcacacgtctgaactccagtca | Spec-seq library |
| Lib_5 | GCTATCTAGTGCTCAGTACGCACTGTTTAAGGAAACT(N:25252525)(N)(N)(N)GCAGGACCAGGTCATGagatcggaagagcacacgtctgaactccagtca | Spec-seq library |
| Lib_6 | GCTATCTAGTGCTCAGTACGCACTGTTTAAGGAAACTTCTG(N:25252525)(N)(N)(N)GACCAGGTCATGagatcggaagagcacacgtctgaactccagtca | Spec-seq library |
| Lib_7 | GCTATCTAGTGCTCAGTACGCACTGTTTAAGGAAACTTCTGGCAG(N:25252525)(N)(N)(N)AGGTCATGagatcggaagagcacacgtctgaactccagtca | Spec-seq library |
| Lib_8 | GCTATCTAGTGCTCAGTACGCACTGTTTAAGGAAACTTCTGGCAGGACC(N:25252525)(N)(N)(N)CATGagatcggaagagcacacgtctgaactccagtca | Spec-seq library |
| Lib_9 | GCTATCTAGTGCTCAGTACGCACTGTTTAAGGAAACTTCTGGCAGGACCAGGT(N:25252525)(N)(N)(N)agatcggaagagcacacgtctgaactccagtca | Spec-seq library |
| Spec-Rev | TGACTGGAGTTCAGACGTGT | Reverse primer for Klenow extension of Spec-seq library |
| GA1-TTTV-OT1-HiTom-F | ggagtgagtacggtgtgcTGCGTACCGAAGGTGGAGAG | Off-target analysis |
| GA1-TTTV-OT1-HiTom-R | gagttggatgctggatggTTCCCCTAAGCTACCACGTG | Off-target analysis |
| GA1-TTTV-OT2-HiTom-F | ggagtgagtacggtgtgcTGCCACGTGGTGCTCTAATA | Off-target analysis |
| GA1-TTTV-OT2-HiTom-R | gagttggatgctggatggAATGTGGAGGGGAAGCGTAG | Off-target analysis |
| GA1-TTTV-OT3-HiTom-F | ggagtgagtacggtgtgcctgccacgtggtgctctaat | Off-target analysis |
| GA1-TTTV-OT3-HiTom-R | gagttggatgctggatggtacgtaggtgcgtaccgaag | Off-target analysis |
| GA1-TTTV-OT4-HiTom-F | ggagtgagtacggtgtgcctgacacgtggcgctttaat | Off-target analysis |
| GA1-TTTV-OT4-HiTom-R | gagttggatgctggatggaccgaagttgtagtgtccgg | Off-target analysis |
| GA1-TTTV-OT5-HiTom-F | ggagtgagtacggtgtgcctgccacgtggtgctataat | Off-target analysis |
| GA1-TTTV-OT5-HiTom-R | gagttggatgctggatggacgtaggtgcgtacctatgt | Off-target analysis |
| GC1-TTTV-OT1-HiTom-F | GGAGTGAGTACGGTGTGCGGATGTGGCTATGATGGTGG | Off-target analysis |
| GC1-TTTV-OT1-HiTom-R | GAGTTGGATGCTGGATGGATCACATCCTCCACAGAGGG | Off-target analysis |
| GC1-TTTV-OT2-HiTom-F | GGAGTGAGTACGGTGTGCATCCAAGGGCCACTTCTACG | Off-target analysis |
| GC1-TTTV-OT2-HiTom-R | GAGTTGGATGCTGGATGGATTCAGGGCCAATGCTGGAA | Off-target analysis |
| GC1-TTTV-OT3-HiTom-F | GGAGTGAGTACGGTGTGCATCACATCCTCCACAGAGGG | Off-target analysis |
| GC1-TTTV-OT3-HiTom-R | GAGTTGGATGCTGGATGGGGATGTGGCTATGATGGTGG | Off-target analysis |
| GC1-TTTV-OT4-HiTom-F | GGAGTGAGTACGGTGTGCCAGGTAATGGATCTCCAGCCTT | Off-target analysis |
| GC1-TTTV-OT4-HiTom-R | GAGTTGGATGCTGGATGGCTTTTGAGTTTTGGTGGCACTG | Off-target analysis |
| GC1-TTTV-OT5-HiTom-F | GGAGTGAGTACGGTGTGCGGACATGGCCATGATGGTGA | Off-target analysis |
| GC1-TTTV-OT5-HiTom-R | GAGTTGGATGCTGGATGGGAAATTCCTTCACGGGTAGACC | Off-target analysis |
| GA1-TTV-OT1-HiTom-F | GGAGTGAGTACGGTGTGCCACGGGAAGGAGTAACCATTG | Off-target analysis |
| GA1-TTV-OT1-HiTom-R | GAGTTGGATGCTGGATGGCCGACACGTGGTGCTCTAATA | Off-target analysis |
| GA1-TTV-OT2-HiTom-F | GGAGTGAGTACGGTGTGCTAAGGGAGGAGGAGGTTGGT | Off-target analysis |
| GA1-TTV-OT2-HiTom-R | GAGTTGGATGCTGGATGGCTTCCTCAAAACGCTCTCAAGC | Off-target analysis |
| GA1-TTV-OT3-HiTom-F | GGAGTGAGTACGGTGTGCACCCTCAAGCTAGCACTTGG | Off-target analysis |
| GA1-TTV-OT3-HiTom-R | GAGTTGGATGCTGGATGGGTGGAGATGTGGGACCTTGT | Off-target analysis |
| GA1-TTV-OT4-HiTom-F | GGAGTGAGTACGGTGTGCGACACGTGGCGCTGTAATAAA | Off-target analysis |
| GA1-TTV-OT4-HiTom-R | GAGTTGGATGCTGGATGGCGGAAGGATCGTAACCACTG | Off-target analysis |
| GA1-TTV-OT5-HiTom-F | GGAGTGAGTACGGTGTGCGTACCGAAGGTGGAGAGGTG | Off-target analysis |
| GA1-TTV-OT5-HiTom-R | GAGTTGGATGCTGGATGGTCACATCGCAGTAATCTCGGT | Off-target analysis |
| GC1-TTV-OT1-HiTom-F | GGAGTGAGTACGGTGTGCGGGAGATGCTGCATTTTTGC | Off-target analysis |
| GC1-TTV-OT1-HiTom-R | GAGTTGGATGCTGGATGGCTCAAATTCATCATGGAGCTTCTGG | Off-target analysis |
| GC1-TTV-OT2-HiTom-F | GGAGTGAGTACGGTGTGCAGGGCCACTTCTACGGATAG | Off-target analysis |
| GC1-TTV-OT2-HiTom-R | GAGTTGGATGCTGGATGGCTGATTCAGGGCCAATGCTG | Off-target analysis |
| GC1-TTV-OT3-HiTom-F | GGAGTGAGTACGGTGTGCTGGATGTGGGCATGATACTGA | Off-target analysis |
| GC1-TTV-OT3-HiTom-R | GAGTTGGATGCTGGATGGACACGATCCATACAGGGCAT | Off-target analysis |
| GC1-TTV-OT4-HiTom-F | GGAGTGAGTACGGTGTGCTCGAAACCCAGCTGCTGATG | Off-target analysis |
| GC1-TTV-OT4-HiTom-R | GAGTTGGATGCTGGATGGCCATTTTTCAGGAGCTAGCTCG | Off-target analysis |
| GC1-TTV-OT5-HiTom-F | GGAGTGAGTACGGTGTGCCCTACCTTCCAAAGGAACCCT | Off-target analysis |
| GC1-TTV-OT5-HiTom-R | GAGTTGGATGCTGGATGGAAAACTAAAGGCCCTGCGTG | Off-target analysis |
| SISGR-crRNA1-rh-R | gtgactggagttcagacgtgtgctcttccgatctACGCAACCTTAGTCCTACCTT | rhAmp-seq primer for tomato |
| SISGR-crRNA1-rh-F | acactctttccctacacgacgctcttccgatctCCAGGAAAGTTGCCAAGAAC | rhAmp-seq primer for tomato |
| SISGR-crRNA2-rh-F | acactctttccctacacgacgctcttccgatctTCCATTGCCACATTAGTGGA | rhAmp-seq primer for tomato |
| SISGR-crRNA2-rh-R | gtgactggagttcagacgtgtgctcttccgatctGAAGGATCTGACACAGGACCA | rhAmp-seq primer for tomato |
| SlBlc-rh-F | acactctttccctacacgacgctcttccgatctAGCCTCTGAAGCTTGGTTTTA | rhAmp-seq primer for tomato |
| SlBlc-rh-R | gtgactggagttcagacgtgtgctcttccgatctCACCACCAACCACAGCTAGA | rhAmp-seq primer for tomato |
| SlLCY-B1-rh-F | acactctttccctacacgacgctcttccgatctCCACATCATGGTTTTGCTGT | rhAmp-seq primer for tomato |
| SlLCY-B1-rh-R | gtgactggagttcagacgtgtgctcttccgatctACCCCTTTTGAAGGGTCATA | rhAmp-seq primer for tomato |
| SlLCY-B2-rh-F | acactctttccctacacgacgctcttccgatctCCGATAATGATCACGTCGAA | rhAmp-seq primer for tomato |
| SlLCY-B2-rh-R | gtgactggagttcagacgtgtgctcttccgatctTGTCCAACAAAATCCTTCTTTTC | rhAmp-seq primer for tomato |
| SlLCY-E-rh-F | acactctttccctacacgacgctcttccgatctTTTTCAGATCTTGGTCTTCAAGC | rhAmp-seq primer for tomato |
| SlLCY-E-rh-R | gtgactggagttcagacgtgtgctcttccgatctCGATTCGTATGACCTAAGGAACA | rhAmp-seq primer for tomato |
