# Supplementary material for: Boosting genome editing efficiency in human cells and plants with novel LbCas12a variants
Source: Genome Biol. 2023 Apr 30;24:102. doi: 10.1186/s13059-023-02929-6 (PMC10150537; doi:10.1186/s13059-023-02929-6)
Supplement: Supplementary file 3 — Additional file 3. Review history. [file 13059_2023_2929_MOESM3_ESM.docx]

Review History

**First round of review**

**Reviewer 1**

**Are you able to assess all statistics in the manuscript, including the appropriateness of statistical tests used?** Yes, and I have assessed the statistics in my report.

**Comments to author:**

Cas12a homologues are one of the most widely used tools for genome editing in various eukaryotes. A very prominent example is LbCas12a, which is very popular in plant biology and breeding. Over the last years a row of papers was published for several Cas12a homologues showing that specific point mutations can change PAM recognition or improve enzyme activity. The manuscript of Zhang et al. now to my knowledge for the first time used an unbiased high throughput screen in E. coli spanning the complete LbCas12a ORF to obtain activity enhancing mutations. After the validation in human cells and combination of the individual mutants the variant RVQ (G146R/R182V/E796Q) turned out to be more than twice as efficient as the wild type enzyme in mutation induction.

The RVQ triple mutant also shows enhance editing efficiencies in rice and tomato protoplasts: Surprisingly the effect is much stronger for rice and minor (and only at 25C) for tomato. It has been shown by others before, that the mutation D156R is also resulting in higher editing efficiency especially at lower temperatures, so the authors constructed a quadruple mutant RRVQ.

The different variants were then compared in stably transformed rice. Under these conditions using the canonical PAM TTTV RRVQ did not perform better than D156R. In contrast with the non-canonical TTV RRVQ resulted in much higher editing efficiencies than all other LbCas12a variants. On the other side in popular the quadruple mutant RRVQ performed much better than D156R.

I think this paper supplies the community with interesting new results. The LbCas12a variant RRVQ is a novel and attractive tool that will be very helpful for editing. I guess much further improvement of LbCas12a will hardly be possible. Nevertheless, reading the paper I was a bit dissatisfied as two obvious questions were not addressed by the authors:

Why there are no data on editing efficiency of the quadruple mutant RRVQ in mammalian cells presented?

Why no SEAM/Spec for a quadruple mutant RRVQ was performed?

I also would include the D156R mutation in the structure shown in figure 2B

**Reviewer 2**

**Are you able to assess all statistics in the manuscript, including the appropriateness of statistical tests used?** Yes, and I have assessed the statistics in my report.

**Comments to author:**

In this manuscript, the authors conducted saturation mutagenesis of LbCas12a in E. coli and identified 1977 positive point mutations, an optimal LbCas12a variant (RVQ: G146R/R182V/E795Q) and LbCas12a-RRVQ has been further identified. RRVQ recognizes a simplified TTV PAM and showed stable and robust genome editing activity in both human cells and plants. Their findings will be of high interest for people in CRISPR community. Generally, the quality of the reported work is high, the data is strong and complete.

Line 35, page 7, LbCas12a-RVQ is temperature-sensitive in rice, please make a revision here.

Line 54, page 13, Plasmid encoding LbCas12a under the control of T7 promoter? Or Lac promoter?

Please check the references carefully and revise them.

**Reviewer 3**

**Are you able to assess all statistics in the manuscript, including the appropriateness of statistical tests used?** Yes, and I have assessed the statistics in my report.

**Comments to author:**

The manuscript describes the protein engineering of LbCas12a through saturation mutagenesis of the full CDS in E. coli and high-throughput screening. A total of 1,977 active sites were identified, from which 24 sites were selected for further testing and combination screening in animal cells, ultimately resulting in the identification of the RVQ combination with the highest activity. In addition, the D156R site was incorporated to improve low-temperature tolerance and increase editing activity in plants. The manuscript presents a novel approach for CRISPR-Cas protein engineering, consistent with the scope of this journal, and is recommended for acceptance.

A minor comment: You should specify the criteria used to select the 24 active sites from the 1,977 identified, as well as provide a comparison of their activity in E. coli and animal cells.

**Authors Response**

**Point-by-point responses to the reviewers’ comments:**

We would like to express our gratitude for the valuable comments provided on our manuscript titled 'Boosting genome editing efficiency in human cells and plants with novel LbCas12a variants'. We appreciate your interest in publishing it. In this revision, we have corrected an error. In the previous submission, we had mistakenly mentioned LbCas12a-RVQ and LbCas12a-RRVQ, which should have been LbCas12-RV and LbCas12a-RRV, respectively. Subsequently, we have included a comprehensive comparison of four LbCas12a variants (RV, RRV, RVQ, and RRVQ) in rice protoplast (Fig. 3B), which is more detailed than Fig. S4 in the previous submission. Therefore, we have removed Fig. S4 from the manuscript. Similarly, we expanded the comparison of more variants in stable transgenic poplar plants and our data show that LbCas12a-RRV is a better variant in poplar (Fig. 5), consistent with the editing data from rice stable transgenic plants (Fig. 4). By contract, we were able to show that the real Lb-Cas12a-RRVQ variant is less efficient than the RVQ variant for genome editing in human cells (Fig. 1C), rice cells (Fig. 3B), and poplar plants (Fig. 5). Moreover, this correction backed with more experimental data didn’t change our conclusion that LbCas12a-RVQ and LbCas12a-RRV enable robust genome editing in human cells and plants, respectively. All changes are highlighted in red in the revised manuscript. Here, we provide a point-to-point response to address reviewers’ questions.

Reviewer #1

Cas12a homologues are one of the most widely used tools for genome editing in various eukaryotes. A very prominent example is LbCas12a, which is very popular in plant biology and breeding. Over the last years a row of papers was published for several Cas12a homologues showing that specific point mutations can change PAM recognition or improve enzyme activity. The manuscript of Zhang et al. now to my knowledge for the first time used an unbiased high throughput screen in E. coli spanning the complete LbCas12a ORF to obtain activity enhancing mutations. After the validation in human cells and combination of the individual mutants the variant RVQ (G146R/R182V/E796Q) turned out to be more than twice as efficient as the wild type enzyme in mutation induction.

The RVQ triple mutant also shows enhance editing efficiencies in rice and tomato protoplasts: Surprisingly the effect is much stronger for rice and minor (and only at 25C) for tomato. It has been shown by others before, that the mutation D156R is also resulting in higher editing efficiency especially at lower temperatures, so the authors constructed a quadruple mutant RRVQ.

The different variants were then compared in stably transformed rice. Under these conditions using the canonical PAM TTTV RRVQ did not perform better than D156R. In contrast with the non-canonical TTV RRVQ resulted in much higher editing efficiencies than all other LbCas12a variants. On the other side in popular the quadruple mutant RRVQ performed much better than D156R.

I think this paper supplies the community with interesting new results. The LbCas12a variant RRVQ is a novel and attractive tool that will be very helpful for editing. I guess much further improvement of LbCas12a will hardly be possible.

Authors’ response: We thank Review 1 for the positive feedback. Please refer to our general response that addressed an error on mislabeling some of the LbCas12a variants. After correction, our data provided better consistency of genome editing data with these reagents in human cells and plant cells. We showed the RVQ variant is better than RRVQ. Furthermore, our more extensive research in plants demonstrated that the RRV variant is most robust among all compared.

Also, we agree it may be hard to significantly improve LbCas12a as we have shown here that stacking more beneficial mutations does not necessarily improve LbCas12a editing activity. Given that we have identified 1977 potentially beneficial mutations, it is impossible to try all the potential combination of double, triple or higher order mutations. On the other hand, these mutations provide a valuable resource for further testing. We probably have to rely on AI to come up with some predictions, along with some intelligent guess based on RNP:DNA structure, before we can efficiently screen more variants to further improve LbCas12a in eukaryotic cells.

In addition, our approach should be widely applicable to engineering other Cas nucleases for improvement.

Nevertheless, reading the paper I was a bit dissatisfied as two obvious questions were not addressed by the authors:

Q1: Why there are no data on editing efficiency of the quadruple mutant RRVQ in mammalian cells presented?

Authors’ response: Thanks for your question. We included the editing efficiency of Lb-RRVQ in human cells in Figure 2C. Lb-RRVQ showed reduced editing efficiency compared with Lb-RVQ, which is consistent with the results in plants (Figure 3B and 5B).

Q2: Why no SEAM/Spec for a quadruple mutant RRVQ was performed?

Authors’ response: Thanks for bringing up this question. Since the quadruple mutation RRVQ compromised the robust editing efficiency of LbCas12a-RVQ (Figure 2C), we didn’t perform the SEAM/Spec analysis on the LbCas12a-RRVQ.

Q3: I also would include the D156R mutation in the structure shown in figure 2B.

Authors’ response: Thanks for your suggestion. We have added D156R mutation in the protein structure of LbCas12a in figure 2B. As we can see, both G146R and D156R mutation are close to each other and appear to interact with dsDNA close to the PAM site.

Reviewer #2

In this manuscript, the authors conducted saturation mutagenesis of LbCas12a in E. coli and identified 1977 positive point mutations, an optimal LbCas12a variant (RVQ: G146R/R182V/E795Q) and LbCas12a-RRVQ has been further identified. RRVQ recognizes a simplified TTV PAM and showed stable and robust genome editing activity in both human cells and plants. Their findings will be of high interest for people in CRISPR community. Generally, the quality of the reported work is high, the data is strong and complete.

Authors’ response: We thank Review 2 for the positive feedback. Please refer to our general response that addressed an error on mislabeling some of the LbCas12a variants. After correction, our data provided better consistency of genome editing data with these reagents in human cells and plant cells. We showed the RVQ variant is better than RRVQ. Furthermore, our more extensive research in plants demonstrated that the RRV variant is most robust among all compared. We have made corrections to our Addgene deposition as well. We cannot wait to disseminate these reagents (upon publishing of this paper), which would be of interest to those who conduct genome editing in any organism. This means our work will potentially benefit a large scientific community.

Q1: Line 35, page 7, LbCas12a-RVQ is temperature-sensitive in rice, please make a revision here.

Authors’ response: Thanks for your comment on this. Based on our results, LbCas1a-RV showed higher editing efficiency in low temperature than WT LbCas12a at all target sites of rice and tomato at higher RNP concentration (Figure 3D and 3F). At lower RNP concentration, LbCas12a-RV showed higher editing efficiency than WT LbCas12a at 3 out of 4 target sites in rice and 5 out of 6 targets in tomato (Additional Fig S3). Thus, LbCas12a-RV is more tolerant to low temperature compared to WT LbCas12a. However, we agree our previous sentence is too strong and could be misleading. We have changed it in the revised manuscript to “Thus, the LbCas12a-RV variant is robust for plant genome editing at variable temperatures.”

Q2: Line 54, page 13, Plasmid encoding LbCas12a under the control of T7 promoter? Or Lac promoter? IDT

Authors’ response: Thanks for your question. The expression of LbCas12a is under the control of T7 promoter with Lac operator. This is the same as the standard pET28 vector for recombinant protein expression in E.coli BL21DE3.

Q3: Please check the references carefully and revise them.

Authors’ response: Thanks for your suggestion. We have carefully checked each cited reference and corrected the format inconsistence.

Reviewer #3

The manuscript describes the protein engineering of LbCas12a through saturation mutagenesis of the full CDS in E. coli and high-throughput screening. A total of 1,977 active sites were identified, from which 24 sites were selected for further testing and combination screening in animal cells, ultimately resulting in the identification of the RVQ combination with the highest activity. In addition, the D156R site was incorporated to improve low-temperature tolerance and increase editing activity in plants. The manuscript presents a novel approach for CRISPR-Cas protein engineering, consistent with the scope of this journal, and is recommended for acceptance.

Authors’ response: We thank Reviewer 3 for the positive comments. Please refer to our general response that addressed an error on mislabeling some of the LbCas12a variants. After correction, our data provided better consistency of genome editing data with these reagents in human cells and plant cells. We showed the RVQ variant is better than RRVQ. Furthermore, our more extensive research in plants demonstrated that the RRV variant is most robust among all compared. In addition to these novel LbCas12a nucleases, our success demonstrates this protein engineering approach can be applied to improve other Cas12a nuclease as well, making our work appealing to anyone who has interest in improving genome editing tools or using such tools.

Q1: You should specify the criteria used to select the 24 active sites from the 1,977 identified, as well as provide a comparison of their activity in E. coli and animal cells.

Authors’ response: Thanks for your comments. We have added the selection criteria in the manuscript. Specifically, the 1977 mutations were first ranked by the phenotype (i.e. enrichment score), and then prioritized over residues on the DNA / R-Loop binding interface, as improving the DNA / R-loop binding affinity has been shown to improve the activity of many nucleases throughout all systems. To rule out false-positives from the screen, we first determined the activity of individual mutants in E.coli (i.e. measuring the depletion of toxin-expression plasmids), and then purified 24 individual LbCas12a point mutations for human cell evaluation. We acknowledge the fact that not all positive hits in the context of E.coli translate to human cell for a variety of possibilities (i.e. reducing toxicity in E.coli without affecting activity, modulating expression level in E.coli specifically, and perturbing stability of LbCas12a as recombinant protein), such as S1020E and K1121D. However, most point mutations localized on the interface of DNA binding translated to the human system, indicating that the major rate-limiting step of gene editing in human/plant cells is the DNA binding affinity.

**Second round of review**

**Reviewer 1**

The authors addressed all my concerns. This is a very valuable paper and it should be published without further ado.
